# Supplementary material for: Racism and access to maternal health care among garo indigenous women in Bangladesh: A qualitative descriptive study
Source: PLoS One. 2023 Nov 30;18(11):e0294710. doi: 10.1371/journal.pone.0294710 (PMC10688635; doi:10.1371/journal.pone.0294710)
Supplement: S1 Checklist — (DOCX) [file pone.0294710.s001.docx]

**S1. Interview checklists**

1. What do you think about the possible health illness during pregnancy, delivery, and after the delivery?
2. Do you have any experience with health illnesses? What did you do to be cured?
3. What are your community practices related to health care during pregnancy, delivery, and after the delivery? Do you follow these? If yes/no, why?
4. How would you consider the effectiveness of your community practices to avoid any health illness during pregnancy, delivery, and after the delivery?
5. Which types of care (hospital care/community practices of care during pregnancy, delivery, and after the delivery) are important to you for ensuring your good health? Why?
6. Do you think seeking care from a hospital is important during pregnancy, delivery, and after the delivery? If yes/No, why?
7. How often have you visited the hospital for pregnancy, delivery, and post-delivery care? If not, why?
8. What do you know about seeking care from the hospital during pregnancy, delivery, and after the delivery? How do you know this?
9. Did you comfortable seeking care from the hospital? If yes/No, why?
10. Do you have any experience seeking care from the hospital? If yes, how were the services?
11. Do you have anything further to add?

Thank for your participation.
