# Supplementary material for: Racism and access to maternal health care among garo indigenous women in Bangladesh: A qualitative descriptive study
Source: PLoS One. 2023 Nov 30;18(11):e0294710. doi: 10.1371/journal.pone.0294710 (PMC10688635; doi:10.1371/journal.pone.0294710)
Supplement: S1 Dataset — (DOCX) [file pone.0294710.s002.docx]

**S1. Data Set**

1. During my last pregnancy, I saw a big difference in how doctors treated non-Garo women compared to me at the hospital. They got good care with all they needed, but the doctor said they didn't have enough for me. I was surprised when a non-Garo woman got proper care for the same problem. In our community, it's hard to access good medical facilities, so getting the care we need during pregnancy is tough. Doctors often use shortages of machines and staffs as excuses, making things even harder for us (N4, Middle-class, Age, 20).
2. due to the gynecologists' preference for non-Garo women, pregnancy care from hospital becomes impossible for us. But we don't expect this (L8, Low-class, Age-26).
3. "Yes... Honestly, I can't recall a time when I went to a doctor's office for maternal care after facing discrimination during my first visit (L3, Middle-class, Age-22)"
4. I felt deeply troubled by the lack of alignment between the available healthcare at the hospital and our community's pregnancy healthcare practices. Following our cultural traditions is essential to us, and it was challenging for me to comprehend and accept healthcare practices that didn't respect our customs. It left me feeling confused and uneasy. (L6, Low-class, Age-20).
5. I don't understand why the doctors won't let us meet the midwife before giving birth. It's essential for us, and they don't seem to care about our cultural needs. They mistreat us, saying they can't do anything because of the hospital's rules (N6, Low-class, Age-20).
6. we can't go inside the delivery room without wearing the hospital's dress, given by the doctors. It's unfair and against our norms related to birth delivery because many women already wear their clothes during delivery (L1, Low-class, Age-21).
7. I think the doctors don't understand our ways and beliefs. They don't involve our community in decisions. Our traditions are ignored, making us feel disconnected. They don't know much about our healing practices, so they prefer their own ways (L12, High-class, Age-26).
8. I am deeply concerned about absence Garo female doctors. The non-Garo doctors does not have any care and respect for us and seeking care from non-Garo male doctor is against out our culture because we can’t share our health problem to a male doctor (L5, Middle class, Age-19).
9. Distance cost isn't all. Hospitals want money. Despite their claim, they don't charge 5 BDT. They demand more. Traveling makes you hungry, but food is expensive. We must spend all we earn if we go. She also adds, we don't control travel and treatment costs. They ignore our issue. They said "you have to follow the hospital rules" whenever we told them our problem. What else can we do but avoid it? (L2, Middle-class, Age-30).
10. They (the clinicians) don’t understand what we're saying. We don't want to be treated by him because he's aggressive and doesn't know how. Our Garo women went there with health issues like jak jachak dalya (swelling of feet and hands), wakkalna ha'sika (vomiting), matha betha (dazziness), Hapani (shortness of breath), and bikma chikki sadika (abdominal pain), but the doctor didn't listen. They give us medicine even though they don’t understand (N5, Low-class, Age-24).
11. Though I can't pronounce Bengali words well, I can explain my health issues to doctors. But, I'm hesitant to go again because the doctors laughed at me when I talked about my health issues a few months ago. The doctors replied with medical jargon. He laughed again when I asked him to explain these. I felt insulted (L7, Low-class, Age-27).
12. Our culture forbids delivering babies by cutting the belly. They touch our private organ during mid-pregnancy visits. Permission was never sought. They forcefully violate our culture (N2, Middle-class, Age-20).
13. The doctors don't listen to our cultural needs, instead blaming us for our customs, such as not cutting the belly and reciting the delivery mantra. They said we are responsible for our health complications, especially birthing complications (N5, Low-class, Age-19).
14. Whenever to visit the doctor, they stigmatize us for our belief in our community norms related to pregnancy health care. Disrespecting our community practices of not cutting the belly for delivery, they forced us to cut the belly. In fact, the doctor decides how to deliver, not we (N9, Low-class, Age-20).
15. Now, I do things my way when it comes to taking care of myself during pregnancy. I don't go to the hospital because they don't understand us. They make me feel small and don't listen to what I say. (N5, Low-class, Age-19).
16. No, it's not just because of doctors’ blaming and discrimination. Also, because our community women who are supportive to each other. Our elders know so much, and I trust them. All these gave me courage to decide to avoid going hospital (N5, Low-class, Age-19).
17. Hospital-level care is value-less to me because they disrespect us. When I need help with my health, I go to our traditional healers. They know our beliefs and take care of us with respect. I want to keep our traditions alive and stay connected to who we are (L7, Low class, Age-27).
18. I knew from my neighbor women and mother, who had their previous pregnancy experiences that if I follow our cultural practices, then there will be no pregnancy health illness. So, why should I visit hospital to be humiliated and encounter discrimination? I don't know. Probably would have sent me to a free hospital maybe (L7, Low-class, Age-27).
19. We have several norms for a safe pregnancy, including avoiding houses where someone has died, not using mouth to fan fire smoke while cooking, eating locally sourced and culturally specific foods, spending time outdoors, participating in traditional activities, and seeking solace in natural environments. We also follow rituals and ceremonies related to pregnancy and rely on medicinal herbs and plants for managing pregnancy discomforts, promoting healthy labor, and aiding postpartum recovery. Additionally, we seek care from traditional healers and birth attendants within our own community (L8, Middle-Class, Age-20).
20. I was able to avoid getting stigmatized by the doctors when I started following our community norms of pregnancy healthcare, I had good health care without making further visit to the hospital. ....I went to traditional healer to all my prenatal visits...I had it good (N11, Middle-class, Age-20).
