# Supplementary material for: Racism and access to maternal health care among garo indigenous women in Bangladesh: A qualitative descriptive study
Source: PLoS One. 2023 Nov 30;18(11):e0294710. doi: 10.1371/journal.pone.0294710 (PMC10688635; doi:10.1371/journal.pone.0294710)
Supplement: Attachment — Submitted filename: PONE-D-22-34953_R1.pdf [file pone.0294710.s005.pdf]

PLOS ONE

“They don’t even listen to our voices”: Ethnocentrism and Biomedical Maternity Care Access Among Garo Indigenous Women in Bangladesh

--Manuscript Draft--

**Manuscript Number:**	PONE-D-22-34953R1
**Article Type:**	Research Article
**Full Title:**	“They don’t even listen to our voices”: Ethnocentrism and Biomedical Maternity Care Access Among Garo Indigenous Women in Bangladesh
**Short Title:**	Ethnocentrism and Biomedical Maternity Care Access Among Garo Indigenous Women in Bangladesh
**Corresponding Author:**	Suban Kumar Chowdhury, MSc in Gender and Development Studies<br>University of Rajshahi<br>Rajshahi, BANGLADESH
**Keywords:**	Garo Women; Ethnocentrism; Discrimination; pregnancy; Childbirth
**Abstract:**	Garo, a matriarchal ethnic group in Bangladesh, faces discriminatory health care treatment during pregnancy and childbirth. This study examined how ethnocentrism affects this group of women's experience accessing biomedical maternity care. It did this by using Janevic's conceptual framework from the analytical lens of intersectionality, which helped create the analytical categories. The study followed a qualitative research approach. 24 semi-structured interviews were conducted with Garo expectant mothers with the help of three female research assistants appointed from the Garo community. The interview sessions were mostly focused on knowing about biomedical care seeking during pregnancy and childbirth, the previous or shared experience of biomedical care, and the perception of ethnocentric discrimination in biomedical maternity care. The audio-recorded data were transcribed into the Bengali language first with the help of the research assistant and then transcribed into English. The emerging themes were grouped under institutional, personally-mediated, and internalized ethnocentrism. The emergent themes include inadequate material conditions, absence of culturally competent care, and power dynamics at the institutional level of ethnocentrism, interpersonal hierarchy, language barrier, use of non-sensical medical jargon, and community practices of maternity care and miscommunication at the level of personally-mediated ethnocentrism, and the themes emerged under the domain of internalized ethnocentrism includes less agency, self-devaluation, and unworthiness of biomedical care. These themes originated from a shared understanding and experience of hospital-level treatment. The three levels of ethnocentrism intersect and produce gendered power structures, masculinist attitudes, prejudice, and stigmatization, making it difficult for Garo mothers to access maternity care. The author proposes a clinician intercultural competency training program (CICTP), and institutional-level initiatives are needed to improve hospital staffing and provide culturally competent care.
**Order of Authors:**	Suban Kumar Chowdhury, MSc in Gender and Development Studies
**Response to Reviewers:**	Dear respected reviewers,<br>Thank you very much for the valuable comments and guidelines. I tried my best to address all the review comments. I have attached a file 'Response to the Reviewers' listing all of the review comments and my responses to these.<br><br>Thank you again for the kind supports.<br>Kind Regards.<br>The Author
**Additional Information:**	
**Question**	**Response**
**Financial Disclosure**<br><br>Enter a financial disclosure statement that	NO

Powered by Editorial Manager® and ProduXion Manager® from Aries Systems Corporation

describes the sources of funding for the work included in this submission. Review the submission guidelines for detailed requirements. View published research articles from *PLOS ONE* for specific examples.<br><br>This statement is required for submission and **will appear in the published article** if the submission is accepted. Please make sure it is accurate.<br><br>**Unfunded studies**<br>Enter: *The author(s) received no specific funding for this work.*<br><br>**Funded studies**<br>Enter a statement with the following details:<br>• Initials of the authors who received each award<br>• Grant numbers awarded to each author<br>• The full name of each funder<br>• URL of each funder website<br>• Did the sponsors or funders play any role in the study design, data collection and analysis, decision to publish, or preparation of the manuscript?<br>• **NO** - Include this sentence at the end of your statement: *The funders had no role in study design, data collection and analysis, decision to publish, or preparation of the manuscript.*<br>• **YES** - Specify the role(s) played.<br><br>* typeset	
**Competing Interests**<br><br>Use the instructions below to enter a competing interest statement for this submission. On behalf of all authors, disclose any competing interests that could be perceived to bias this work—acknowledging all financial support and any other relevant financial or non-financial competing interests.<br><br>This statement is required for submission and **will appear in the published article** if the submission is accepted. Please make sure it is accurate and that any funding	The authors have declared that no competing interests exist.

sources listed in your Funding Information later in the submission form are also declared in your Financial Disclosure statement.<br><br>View published research articles from *PLOS ONE* for specific examples.<br><br>**NO authors have competing interests**<br><br>Enter: *The authors have declared that no competing interests exist.*<br><br>**Authors with competing interests**<br><br>Enter competing interest details beginning with this statement:<br><br>*I have read the journal's policy and the authors of this manuscript have the following competing interests: [insert competing interests here]*<br><br>* typeset	
**Ethics Statement**<br><br>Enter an ethics statement for this submission. This statement is required if the study involved:<br><br>• Human participants<br>• Human specimens or tissue<br>• Vertebrate animals or cephalopods<br>• Vertebrate embryos or tissues<br>• Field research<br><br>Write "N/A" if the submission does not require an ethics statement.<br><br>General guidance is provided below. Consult the submission guidelines for detailed instructions. **Make sure that all information entered here is included in the Methods section of the manuscript.**	As this research is the partial part of the author's MSc thesis project which has done during pursuing MSc degree from the Asian Institute of Technology (AIT), ethics approval has received from AIT ethics committee.

**Format for specific study types**<br><br>**Human Subject Research (involving human participants and/or tissue)**<br>• Give the name of the institutional review board or ethics committee that approved the study<br>• Include the approval number and/or a statement indicating approval of this research<br>• Indicate the form of consent obtained (written/oral) or the reason that consent was not obtained (e.g. the data were analyzed anonymously)<br><br>**Animal Research (involving vertebrate animals, embryos or tissues)**<br>• Provide the name of the Institutional Animal Care and Use Committee (IACUC) or other relevant ethics board that reviewed the study protocol, and indicate whether they approved this research or granted a formal waiver of ethical approval<br>• Include an approval number if one was obtained<br>• If the study involved *non-human primates*, add *additional details* about animal welfare and steps taken to ameliorate suffering<br>• If anesthesia, euthanasia, or any kind of animal sacrifice is part of the study, include briefly which substances and/or methods were applied<br><br>**Field Research**<br><br>Include the following details if this study involves the collection of plant, animal, or other materials from a natural setting:<br>• Field permit number<br>• Name of the institution or relevant body that granted permission	
**Data Availability**<br><br>Authors are required to make all data underlying the findings described fully available, without restriction, and from the time of publication. PLOS allows rare exceptions to address legal and ethical concerns. See the PLOS Data Policy and FAQ for detailed information.	Yes - all data are fully available without restriction

Powered by Editorial Manager® and ProduXion Manager® from Aries Systems Corporation

A Data Availability Statement describing where the data can be found is required at submission. Your answers to this question constitute the Data Availability Statement and **will be published in the article**, if accepted.<br><br>**Important:** Stating 'data available on request from the author' is not sufficient. If your data are only available upon request, select 'No' for the first question and explain your exceptional situation in the text box.<br><br>Do the authors confirm that all data underlying the findings described in their manuscript are fully available without restriction?	
**Describe where the data may be found in full sentences. If you are copying our sample text, replace any instances of XXX with the appropriate details.**<br><br>• If the data are **held or will be held in a public repository**, include URLs, accession numbers or DOIs. If this information will only be available after acceptance, indicate this by ticking the box below. For example: *All XXX files are available from the XXX database (accession number(s) XXX, XXX.*).<br>• If the data are all contained **within the manuscript and/or Supporting Information files**, enter the following: *All relevant data are within the manuscript and its Supporting Information files.*<br>• If neither of these applies but you are able to provide **details of access elsewhere**, with or without limitations, please do so. For example:<br><br>*Data cannot be shared publicly because of [XXX]. Data are available from the XXX Institutional Data Access / Ethics Committee (contact via XXX) for researchers who meet the criteria for access to confidential data.*<br><br>*The data underlying the results presented in the study are available from (include the name of the third party*	All the data are available

and contact information or URL).<br>• This text is appropriate if the data are owned by a third party and authors do not have permission to share the data.<br><br>* typeset	
Additional data availability information:	Tick here if your circumstances are not covered by the questions above and you need the journal's help to make your data available.

Powered by Editorial Manager® and ProduXion Manager® from Aries Systems Corporation

Manuscript Click here to access/download;Manuscript;Manuscript.docx

"They don't even listen to our voices": Ethnocentrism and biomedical maternity care access among Garo indigenous women in Bangladesh

Suban Kumar Chowdhury[1]

[1] Department of International Relations, University of Rajshahi, Bangladesh.

[*]Suban Kumar Chowdhury

E-mail: skc_ruir@ru.ac.bd

Author's contributions

The author developed the idea of conducting this research from his interest in indigenous women's maternal health care matters. The author reviewed the relevant literature and designed the research instruments to complete this study. The author wrote the initial and final drafts of the manuscript as the principal researcher. For data collection, the author sought help from four female research assistants appointed from the Garo community.

Acknowledgement

The author is thankful to the anonymous reviewers of this manuscript for their kind review and valuable comments. This article is part of the author's 'MSc thesis work' submitted to AIT under Dr Joyee S Chatterjee (program Chair-Gender and Development Studies program- and Assistant Professor at the Department of Development and Sustainability, AIT). Therefore, the author would like to thank Dr Joyee S Chatterjee. Also, the author would like to thank the research participants and the three research assistants (who helped throughout the data collection process).

Abstract

Garo, a matriarchal ethnic group in Bangladesh, faces discriminatory health care treatment during pregnancy and childbirth. This study examined how ethnocentrism affects this group of women's experience accessing biomedical maternity care. It did this by using Janevic's conceptual framework from the analytical lens of intersectionality, which helped create the analytical categories. The study followed a qualitative research approach. 24 semi-structured interviews were conducted with Garo expectant mothers with the help of three female research assistants appointed from the Garo community. The interview sessions were mostly focused on knowing about biomedical care seeking during pregnancy and childbirth, the previous or shared experience of biomedical care, and the perception of ethnocentric discrimination in biomedical maternity care. The audio-recorded data were transcribed into the Bengali language first with the help of the research assistant and then transcribed into English. The emerging themes were grouped under institutional, personally-mediated, and internalized ethnocentrism. The emergent themes include inadequate material conditions, absence of culturally competent care, and power dynamics at the institutional level of ethnocentrism, interpersonal hierarchy, language barrier, use of non-sensical medical jargon, and community practices of maternity care and miscommunication at the level of personally-mediated ethnocentrism, and the themes emerged under the domain of internalized ethnocentrism includes less agency, self-devaluation, and unworthiness of biomedical care. These themes originated from a shared understanding and experience of hospital-level treatment. The three levels of ethnocentrism intersect and produce gendered power structures, masculinist attitudes, prejudice, and stigmatization, making it difficult for Garo mothers to access maternity care. The author proposes a clinician intercultural competency training program (CICTP), and

institutional-level initiatives are needed to improve hospital staffing and provide culturally competent care.

Introduction

Ethnocentrism-refers to cultural stereotypes, prejudice, discrimination, and an unwillingness to learn from other cultures (1)-is a major maternity health concern (2). However, how it impacts indigenous women's experience accessing maternity care services is often overlooked (3). This is crucial because research conducted in different countries has shown that indigenous women experience higher maternal and infant morbidity rates and mortality linked to ethnocentrism (4–7), (8). For example, healthcare providers' ethnocentric discrimination, which refers to the unjust treatment of individuals or groups based on their ethnic or cultural background (9), in maternity care against indigenous mothers is a well-documented issue in the United States of America and India (10–15). Recent research showed that ethnocentric discrimination and cultural insensitivity in the biomedical maternity care system could lead to feelings of disempowerment, mistrust, and trauma, leading to little interest in healthcare access. This is an important argument for understanding the significance of ethnocentrism as a social determinant of health (16).

Ethnocentrism, however, is a neglected health issue in Bangladesh and other South Asian nations (17). A comprehensive review of indigenous South Asian women's maternal health found that ethnicity affects maternity healthcare access, including varied health conditions and life expectancy, limited biomedical care services, unfriendly hospital staff, inequitable treatment and bias, dissatisfaction, and lack of practical and emotional support (18–20). However, ethnocentrism and maternity care access in Bangladesh are still not being studied, except for a few studies that have examined the experiences of indigenous mothers who receive maternity care (21–23) even though research is increasing worldwide, with studies from the United States (4), Mexico (8), and India (24). Given the evidence that a woman's social

setting caused variation in the experience of maternity healthcare experience (25), a context-specific understanding of ethnocentrism and maternity healthcare access is crucial.

Given the above landscapes, this study examined how ethnocentrism affects Garo indigenous women's experience of biomedical maternity care in Bangladesh. Since 2011, ethnic prejudice against the Garo, one of the world's last close-knit matriarchal communities (26–28), become more due to their recognition as tribal, meaning uncivilized people (29, 30). Such designation implied ethnic inferiority and continued colonial exclusionary tactics (31). Linking to discriminatory postcolonial policies concerning the case of the Garo, a group of scholars claim that the state has overlooked the Garo and other ethnic minority groups in Bangladesh their right to be recognized as indigenous communities and has reified and essentialized their culture and traditions (32). Institutional discourses have portrayed their culture as something to be kept in galleries, separating 'indigenous' people from 'civilization' and linking them to the past (33). In addition, statistics show 62.3% of Garo women did not receive skilled maternity care (34–36). Despite Garo women's limited access to biomedical maternity care, ethnic prejudice has not been studied in this context. The Bangladeshi government's commitment to indigenous-friendly health care requires disaggregated data on indigenous mothers' health, which is lacking (37). Thus, it adds another dimension to undertaking this study. Another rationality lies in previous research findings showing that women's vulnerability to maternal health owing to ethnicity is in the limelight, and maternity care is under question (38,39).

The current study used a framework adapted from Janevic's conceptual underpinning of racism and, thus, framed ethnocentrism under three levels: institutional, personally-mediated, and internalized (40). Institutional-level ethnocentrism is the routine and systemic enactment of ethnocentric discrimination by institutions, policies, and practices that deeply and extensively affect underprivileged indigenous women (41). Thus institutionalized ethnocentrism occurs via commission (activity, habit, social practice, legislation) and omission, manifesting in material condition and power dynamics (42). Personally-mediated ethnocentrism is related to individual action of treating others in a discriminatory way based on ethnicity

and manifests due to disrespectfulness, distrust, devaluation, blaming, and dehumanization (40,43,44). Internalized ethnocentrism refers to the acceptance of own abilities and intrinsic worth by stigmatized ethnic individuals, leading to resignation, helplessness, and hopelessness (40).

Janevic's conceptual framework has been widely used to study indigenous women's discriminatory maternal healthcare access. Most studies have focused on racism (45), ignoring ethnocentrism from an intersectional analytical approach examining how interlocking power systems affect underprivileged women (46). This is crucial because ethnocentrism, the root of racism (47), has distinct social representations (47) (48). Racism is a social construct of attitudes, views, opinions, behavior, practices, and actions (49)(50), while ethnocentrism is how someone sees the world through their meanings (51). Thus, understanding the indigenous mothers' experience of accessing biomedical maternity care requires a more holistic approach emphasizing the intersectionality of institutional, personally-mediated, and internalized ethnocentrism. This study used Janevic's conceptual framework to fill this gap.

Materials and methods

Settings

Kalmakanda is one of 10 administrative Upazila (sub-district) of Netrakona District in northern Bangladesh (52). Out of the 8 unions (village), Lengura and Nazirpur are the two unions that have long been home to most of the Garo people of Bangladesh (53)(54). The study was conducted at these two unions on the northeastern side of Kalmakanda Upazila, with scant maternal healthcare facilities (55,56). The Kalmakanda Hospital has two Union sub-centres and 31 community clinics, but these services are unavailable in either Union. The Upazila hospital has ten doctors, eleven nurses, two male medical assistants, and a cesarean section operated by a non-Garo man gynecologist. Most of the pregnant patients who needed care were not Garo mothers. It means mothers' health is a top concern in the hospital, but not many Garo women use these services.

Map 1: Study area

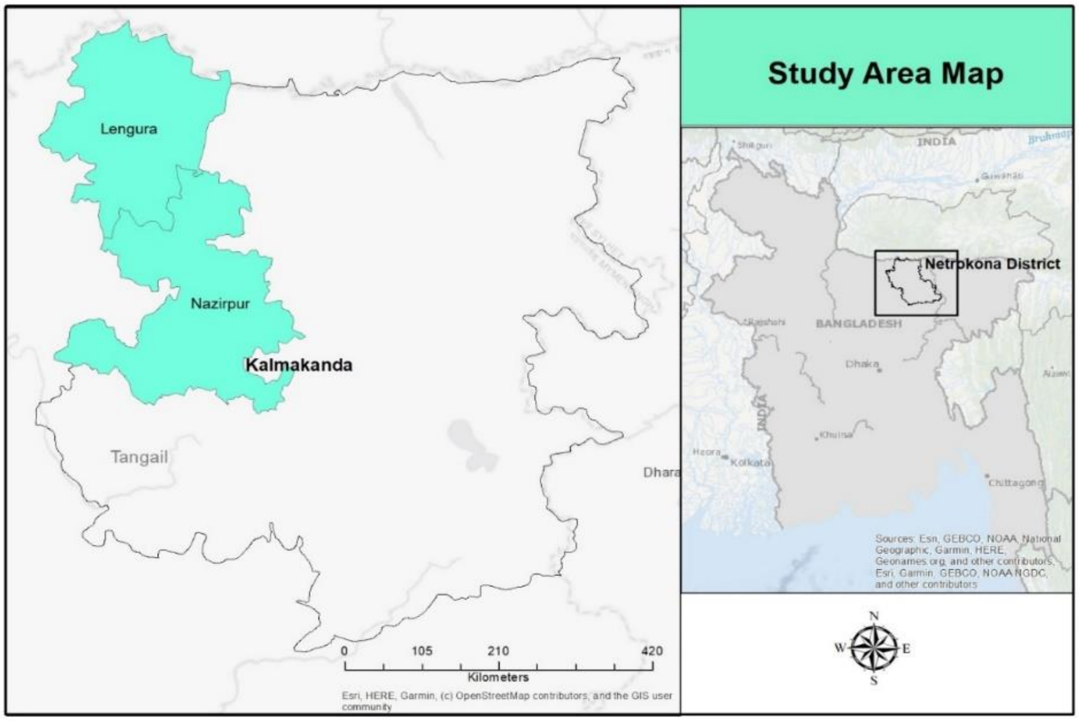

Study Procedures

This qualitative study conducted in-depth interviews with Garo women to understand their perspectives, emphasizing an effort to listen to their unheard voices. The interviews were conducted with assistance from three Garo female research assistants living in the research area.

Sampling and recruitment

Participants were selected using “intersectionality-informed methods”. Such techniques involved “thinking beyond categories like gender, age, education level, and economic class, as additives or alone, create space for the complexity of identities and experiences” (57). It helps to see identities and experiences as multidimensional rather than additive or distinct (46). Thus, the author interviewed 24 Garo moms using purposive sampling (58), specifically "snowball sampling" (59) as an intersectionality-informed method (60). Snowball sampling was utilized since identifying

pregnant and new mothers was difficult. A recruiting matrix was used to ensure age, literacy, and pregnancy diversity.

Data collection

The researcher conducted in-depth interviews with participants from February 2021 to March 2021, using semi-structured interviews and open-ended questionnaires. The in-depth interview questionnaire covered knowledge of biomedical maternal health care, maternal health beliefs, and pregnancy and childbirth health care experiences. Training sessions were organized for research assistants, and checklists were prepared. Each interview lasted at least 50 minutes and was audio-recorded and saved on a password-protected computer. Field notes were also taken.

Analysis

The research assistants first transcribed the audio-recorded data into Bengali because the data were taken in the Mandi language. Then, the author translates these into English. Next, the author analyzed the transcribed data, written in English, using constant comparison and displayed verbatim. After that, the transcripts were coded in four phases: initial codes, refinements, drawing a diagram to structure the codes, and categorizing the themes that indicate the three levels of ethnocentrism's effect on Garo women's access to biomedical maternal care. In this process, the researcher rechecked the codes and themes to ensure that these best reflect the Garo mothers' perspectives.

Ethics

This study is part of the author's MSc thesis at the Asian Institute of Technology (AIT), Thailand. The author received ethics approval from AIT Ethics Committee. Before conducting interviews, participants and their husbands were contacted and informed about the research. Also, verbal

consent was taken before conducting interviews, and the ethics committee approved it. The participants were informed that confidentiality would be maintained and their participation was voluntary and could be withdrawn without consequences. Pseudonyms were used to ensure data privacy.

Results

Participant characteristics

The in-depth interview (IDI) included 24 Garo mothers. **Table 1** lists the number of IDI participants and the inclusion criteria. After nineteen IDI saturation of themes was achieved. Five more IDI were conducted to confirm the saturation of themes and execute an in-depth exploration of some subthemes. The author noticed active engagement of all participants with good interaction with himself and the research assistants.

Table 1: Lists of IDI and KII respondents and their inclusion criteria.

<table>
<tr><td colspan="5">IDI respondents</td></tr>
<tr><td colspan="3">Area</td><td>Number</td><td>Selection criteria</td></tr>
<tr><td rowspan="2">Netrokona District</td><td rowspan="2">Kalmakanda Upazila</td><td>Lengura Union</td><td>n=12</td><td rowspan="2">The Garo women (aged 18 to 35) who were pregnant or had childbirth within a year on the interview date.</td></tr>
<tr><td>Nazirpur Union</td><td>n=12</td></tr>
</table>

Figure 1 visualizes the eight themes from coding. Following Janevic's conceptual framework, these are organized into three levels for clarity. Themes under the institutional level include

material conditions, the absence of culturally appropriate treatment, and power dynamics. Inter-personal hierarchy, language barrier, non-sensical medical terminologies, and community practices of maternity care and misunderstanding are grouped under personally-mediated ethnocentrism. The third level, internalized ethnocentrism, includes less agency and self-devaluation. Based on Figure 1, as visualized below, the following sub-section presents the themes in detail.

Figure 1: Three layered ethnocentrism and the emergent themes

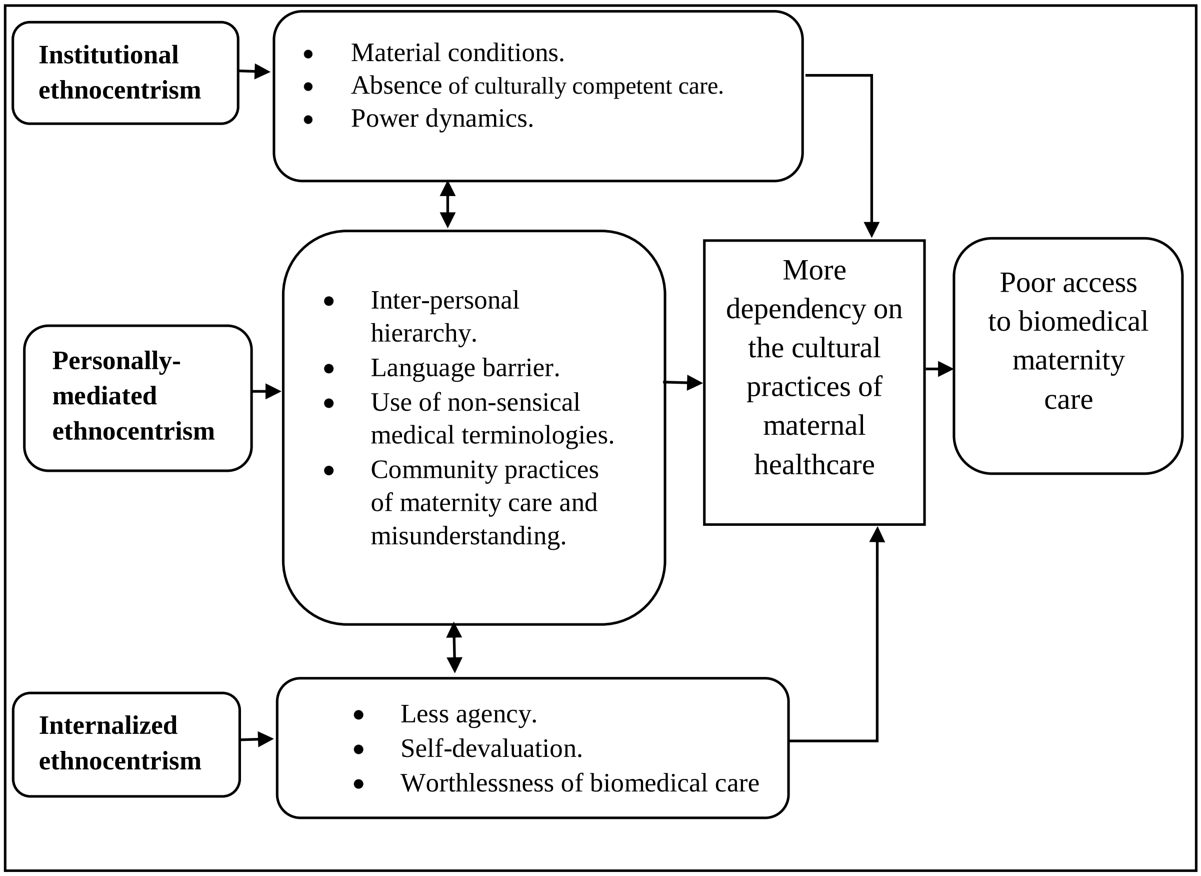

Institutional ethnocentrism

Material conditions and power dynamics represent institutional ethnocentrism. Indigenous women in Bangladesh are marginalized and lack institutional biomedical care (61). This study confirmed

this trend. Mothers often blame poor maternity care access on inadequate service facilities and institutional power dynamics. One mother reported:

> The hospital has no services, making pregnancy care impossible. We have trouble getting care because the only available doctor always found busy with non-Garo women. The man gaini doctor (gynecologist) always serves non-Garo women, ignoring us. What can I do there? I don't go to hospitals like my neighbors (L8, 26).

The interviewed women cited inadequate hospital staffing, discriminatory treatment, and clinicians' priority of caring for local women for not using institutional health facilities. The Garo mothers' accounts also suggest culturally inappropriate birthing care. Cultural issues were most apparent in the hospital's dressing rules and birthing room privacy and the absence of Garo female clinicians. Furthermore, they complained not meeting the midwife before hospital birth. She said:

> Doctors are not Garo women. No one is allowed to enter the delivery room without wearing the hospital's dress provided by them (the doctors). It's unfair because many women use these clothes, already, during their delivery (N6, age 28).

In addition, some mothers highlight the higher cost of seeking care from the Upazila hospital as another reason for not attending institutional care:

> Distance cost isn't all. Hospitals want money. Despite their claim, they don't charge 5 BDT. They demand more. Traveling makes you hungry, but food is expensive. We must spend all we earn if we go. She also adds, we don't control travel and treatment costs. They ignore our issue. They said "you

> have to follow the hospital rules" whenever we told them our problem. What else can we do but avoid it? (L2, age 30).

Due to expensive travel and treatment, Garo mothers avoid biomedical care. These factors must be taken into account when managing maternity health care. It is an example of typical financial cost-driven discrimination in biomedical maternity care. A study found that indigenous women in Bangladesh earn less due to ethnicity (62). Hence, the Garo mothers' denial of biomedical care due to economic cost-driven discrimination shows how institutionalized ethnocentrism affects biomedical care access. Overall, the mothers typically blamed the insufficient human resources, the power of the clinicians for poor access. Also, they present the obligation of following the institutional guidelines defined by the clinicians as a reason for not accessing these services. This illustrates a link to the personally-mediated ethnocentrism, as presented in the following sub-section.

Personally-mediated ethnocentrism

The Garo mothers' experiences with clinicians' personally-mediated ethnocentrism include disrespect, distrust, devaluation, and blaming indigenous cultural practices. Another issue that emerged is the clinicians' ethnocentrism related to language, which made them view Garo mothers' Mandi language use as problematic. These women generally described how a clinician miscommunicated with them, showing that indigenous language speakers are discriminated against because they do not communicate using mainstream language:

> He (the clinician) doesn't understand what we're saying. We don't want to be treated by him because he's aggressive and doesn't know how. Our Garo women went there with health issues like jak jachak dalya (swelling of feet and hands), wakkalna ha'sika (vomiting), matha betha (dazziness), Hapani

(shortness of breath), and bikma chikki sadika (abdominal pain), but the doctor didn't listen. He gives us medicine even though he doesn't understand (N5, age 24).

Few Garo mothers were bilingual; they speak Mandi in the community and Bengali elsewhere. Their accounts suggest they still face discrimination at the Upazila Hospital due to their difficulty pronouncing Bengali words. Data demonstrates that using Bengali in the hospital is emotionally and substantially harder than in daily life. The women also complained about clinicians' using nonsensical medical terminology they were unfamiliar with. As one woman typically reported:

> Though I can't pronounce Bengali words well, I can explain my health issues to doctors. But, I'm hesitant to go again because the doctor laughed at me when I talked about my health issues a few months ago. The doctor replied with medical jargon. He laughed again when I asked him to explain these. I felt insulted (L7, age 27).

The issue of power and domination over the Garo mothers' bodies was highlighted, with the use of force by clinicians, touching private organ without permission, and not giving the placenta to the family to perform cultural rituals as significant barriers. As one mother reported:

> Our culture forbids delivering babies by cutting the belly. They touch our private organ during mid-pregnancy visits. Permission was never sought. My Garo neighbors say that. I won't go. They forcefully violate our culture (N2, age-23).

The woman's quote suggests that clinicians used masculinist power to disregard the mothers' cultural norms for maternity care and forced them to give up their cultural practices to promote biomedical care as the embodiment of modernity. The women's accounts also showed that

clinicians' perception about community maternal health care practices stigmatized them, making it difficult for them to get to Upazila Hospital:

> The doctors don't listen to our cultural needs, instead blaming us for our customs, such as not cutting the belly and reciting the delivery mantra. They said we are responsible for our health complications, especially birthing complications (N5, age 24).

According to mothers, clinicians stigmatize them for respecting indigenous maternity care customs. Most women were afraid to go to a hospital because they thought the clinicians would force C-sections or vaginal incisions, which violate cultural norms and harm reproductive health. Thus, the clinicians’ personally-mediated ethnocentrism stigmatized Garo mothers by attributing serious maternal health complications to the community's maternity healthcare practices. As data suggests, the goal is to place mothers under biomedical supervision without regard to indigenous culture. Thus, the doctor-Garo mother hierarchy is revealed:

> We were forced to cut the belly for delivering the baby. That's why we won't go to the hospital. In fact, the doctor decides how to deliver, not we (N9, age 26).

As evident from the data, the clinicians’ suggestion for C-section to Garo mothers reflects institutionalized ethnocentrism, which ignores the culturally believed health safety of normal birthing. This suggests the interplay between institutional and personally-mediated ethnocentrism, making the Garo mother hesitant to access biomedical maternity care.

Internalized ethnocentrism

Three sub-themes emerged related to internalized ethnocentrism. There are less agency, self-devaluation, and worthlessness in biomedical care. While discussing the effect of the previous experience of being stigmatized, another mother typically reported her less agency in raising voices concerning the perceived and shared negative experiences of ethnocentric discrimination:

> I was embarrassed and disappointed when the doctor told me my cultural beliefs were to blame for my health issues. Since I'm Garo and there's no Garo doctor, I can't comment on the doctor's behavior (N5, age 24).

Self-devaluation comes from the fact that women often blame themselves for how health professionals treat them when they seek medical care:

> We are responsible for how the doctors treat us in the hospital, as we don't understand their language, speak Bengali with them, and dress like them. It's all our fault (N3, age 19).

Interestingly, the interviewed mothers linked their less agency and feeling of self-devaluation with the worthlessness of biomedical care compared to community maternity care. Evidence suggests that their encounters with discrimination and stigmatization at institutional ethnocentrism manifested in material conditions and power dynamics and at the level of personally-mediated ethnocentrism reflected in hierarchical relations. Some studies showed the hegemony of biomedical treatment, defined as the domination of medical care (63), over traditional cures (64)(65), while another found that biomedical care has replaced indigenous mothers' community cultural practices of maternity care (66). This study contradicts these findings, as institutionalized ethnocentrism and clinicians' personally mediated ethnocentrism manifested in hierarchical power

relations and stigmatization strengthen these mothers' dependence on community-based maternity care over biomedical care:

> It is better to seek care at home following our cultural practices, as there is no chance of being insulted by the doctors (L7, age 27).

Thus, it seems that due to the internalization of the intersectional mix of institutional and personally-mediated ethnocentrism which the health institutions imposed on them and which they reproduce by identifying with the dominant biomedical facilities.

Discussions

This study used Janevic's conceptual framing (40) to explore the Garo indigenous mothers' biomedical maternity care experiences. Results showed that Garo women were hesitant to get biomedical care due to inadequate material conditions, human resources, discrimination based on economic status, ethnicity, culturally inappropriate care, barriers related to language, and access to information. A previous study has focused on biomedical care service delivery institutions' neglect of ethnocentric discrimination (17). Data demonstrates that these factors are the dimensions of institutionalized ethnocentrism manifested in ethnocentric practices that create power imbalances between the hospital staff and the Garo mothers. These outcomes led to self-low-agency, self-devaluation, and dependency on community maternity care considering biomedical care as worthless.

The methodological limitations of the current study were driven by the participants' varied traits and emotional responses. By fostering trust, the author was able to get past this limitation. This trust was confirmed when the Garo indigenous mothers refused to talk about their experiences publicly but did so during individual interviews in their homes. The analysis is based on the data

found, but triangulation with health professionals and mainstream women's experiences may contribute much more to research outcomes if contrast is done among these three perspectives.

Ethnocentrism levels often reinforce each other, according to analysis. The hospital's non-indigenous staff's interactions with the Garo indigenous mothers demonstrate a post-colonial mindset and asymmetric power relations. Previous studies in different countries have shown the continuity and persistence of imbalanced power relations between biomedical caregiving institutions and indigenous mothers and their cultural healing related to maternity care (67, 68) (42). Such a pattern also becomes evident in the current study. Bangladesh's health policy segregated the indigenous people from the mainstream population by acknowledging them as a tribe, meaning uncivilized (29), but not as Adivasi (indigenous community) (69). It prevents health practitioners from understanding historical and contemporary contexts and identifying unequal power relations with Garo mothers. Thus, as data suggests, health practitioners following service guidelines reproduce colonial prejudices and stereotypes in an institutionalized context. Resultantly, Garo indigenous patients have found internalized a sense of low agency and self-dehumanization and considered biomedical care unworthy because it undermined cultural maternity care practices. The mothers' strong belief in their culture, close-knit community, and neighborhood settings explain the unworthiness. This is unlike a previous study conducted in the context of Mexico that found mothers abandoning indigenous cultural practices and assimilation with the biomedical care system in the name of modernization (42).

Concerning modernization, other studies have shown that segregationist colonialism and assimilationist nationalism have shaped public institutions' relations with indigenous people

(70)(71). Thus, it creates a racialized "grammar of diversity," an underlying cultural logic of intercultural relations between state representatives and communities (72). Adding to these findings, the present study shows that ethnocentrism interacts with other sources of discrimination (46). For example, as data suggests, two sources are the gendered power hierarchies and clinicians' masculinist attitudes toward Garo mothers, especially in community maternity care and interpersonal interactions. Thus, this finding provides another layer of contribution to a recent study (45).

In the research site, the hospital staff wielded gendered power and saw Garo mothers as responsible for their maternal health complications, wrongfully dependent on their community maternity care practices. Gendered power hierarchy is reflected at the level of personally-mediated ethnocentrism and reproduced masculinist behavior among clinicians towards Garo mothers, causing discrimination, language barriers, miscommunication, and misunderstanding between doctors and Garo mothers about community maternity care and biomedical care. However, institutionalized ethnocentrism underlies this power hierarchy and masculinist attitudes, suggesting its cross-cutting relationship with personally-mediated ethnocentrism. Thus, institutional and personally-mediated ethnocentrism revealed gendered power hierarchies and masculinist attitudes toward indigenous Garo mothers and their maternity care. Based on this finding, the current study suggests further research from the feminist analytical lens of power to understand how gender, ethnicity, and the three levels of ethnocentrism affect Indigenous mothers' access to biomedical maternity care. Because ethnicity and gender are mutually constitutive of ethnocentric discrimination (73), such a future study would be reasonable. However, the study showed that three levels of ethnocentrism intersected and cross-cut, creating a gendered power

hierarchy and masculinist attitudes among clinicians that discriminated against Garo mothers. The clinicians' personally mediated ethnocentrism intertwined with historical colonialism through the subalterization of Garo indigenous mothers, who were perceived as the "others" by doctors. Subalterization, which devalues women and their culture based on their social position across multiple identities, including ethnicity (74), drives colonization (75).

This study emphasized the importance of clinicians' intercultural competency training program (CICTP) that promotes ethnic relations and an institutional policy that increases hospital staffing and culturally sensitive maternity care. The CICTP would eliminate hierarchical relations between health practitioners and indigenous mothers, bias and discrimination against indigenous mothers, miscommunication, and misunderstanding about cultural birthing norms and placenta practices. Indigenous mothers may overcome low agency and self-doubt if clinicians recognize cultural competencies in maternity care and non-discrimination. Indigenous mothers must be convinced that biomedical and community maternity care can improve maternity health together to change their view of biomedical care as worthless.

Conclusion

Ethnocentrism is an important maternity health concern for Garo indigenous women, with institutional ethnocentrism and personally-mediated ethnocentrism manifesting in discrimination and stigmatization due to language and community practices. In this study, data demonstrates internalized ethnocentrism in two different directions. First, a feeling of low agency and self-devaluation, and second, a perception of the worthlessness of biomedical care. Therefore, to overcome ethnocentric discrimination and stigmatization, the study suggests that CICTP training programs and institutional initiatives related to increasing the number of hospital staffing and

providing culturally competent care are strikingly necessary. Such training does not to substitute any existing training program organized for the clinicians, but to inject a strong sense of ethnic-relations to ensuring the delivery of culturally appropriate care acknowledging the ethnic diversity.

References

1. Judd CM, Park B, Ryan CS, Brauer M, Kraus S. Stereotypes and Ethnocentrism: Diverging Interethnic Perceptions of African American and White American Youth. J Pers Soc Psychol. 1995;69(3):460–81.

2. Hine R, Krakouer J, Elston J, Fredericks B, Hunter SA, Taylor K, et al. Identifying and dismantling racism in Australian perinatal settings: Reframing the narrative from a risk lens to intentionally prioritise connectedness and strengths in providing care to First Nations families. Women and Birth. 2023;36(1):136–40.

3. Jordal M, Wahlberg A. Challenges in providing quality care for women with female genital cutting in Sweden–A literature review. Sexual & reproductive healthcare. 2018;17:91–6.

4. Poetry S. Giving Birth in South Florida : A Phenomenological Study of the Pregnancy and Birthing Experiences among Black and Afro-Latina Women. 2020.

5. Petersen EE, Davis NL, Goodman D, Cox S, Syverson C, Seed K, et al. Racial/ethnic disparities in pregnancy-related deaths—United States, 2007–2016. Morbidity and Mortality Weekly Report. 2019;68(35):762.

6. Hartz D, Sherwood J. Midwives working with Aboriginal and Torres Strait Islander women. Midwifery: Preparation for practice. 2019;158–74.

7. Dossetor PJ, Freeman JM, Thorburn K, Oscar J, Carter M, Jeffery HE, et al. Health services for aboriginal and Torres Strait Islander children in remote Australia: A scoping review. PLOS Global Public Health. 2023;3(2):e0001140.

8. Dörr NM, Dietz G. Racism and discrimination in the care of Totonaca women in Veracruz, Mexico: the need for intercultural competencies. Social Medicine. 2022;15(1):42–53.

9. Shiraev EB, Levy DA. Cross-cultural psychology: Critical thinking and contemporary applications. Routledge; 2020.

10. Vedam S, Stoll K, Taiwo TK, Rubashkin N, Cheyney M, Strauss N, et al. The Giving Voice to Mothers study: inequity and mistreatment during pregnancy and childbirth in the United States. Reprod Health. 2019;16(1):1–18.

11. Thapa R, Teijlingen E Van, Regmi PR, Heaslip V. Caste Exclusion and Health Discrimination in South Asia : A Systematic Review. 2021;

12. Kozhimannil KB, Interrante JD, Tofte AN, Admon LK. Severe maternal morbidity and mortality among indigenous women in the United States. Obstetrics and gynecology. 2020;135(2):294.

13. MacDonald C, Steenbeek A. The impact of colonization and western assimilation on health and wellbeing of Canadian aboriginal people. International Journal of Regional and Local History. 2015;10(1):32–46.

14. Green TL, Zapata JY, Brown HW, Hagiwara N. Rethinking bias to achieve maternal health equity: changing organizations, not just individuals. Obstetrics and gynecology. 2021;137(5):935.

15. Adhikari T, Sahu D, Nair S, Saha KB, Sharma RK, Pandey A. Factors associated with utilization of antenatal care services among tribal women: A study of selected States. Indian J Med Res. 2016;144(1):58.

16. Küey L. Clarifying Definitions of „Race “, Racism, and Ethnocentrism. European Psychiatry. 2022;65(S1):S35–S35.

17. Rice ZS, Liamputtong P. Culture and Global Health: Cultural Determinants of Health and Well-Being. Handbook of Global Health. 2020;1–26.

18. Akter S. Examining access to health services for women of reproductive age from ethnic communities in Chittagong Hill Tracts (CHT) of Bangladesh.

19. Thresia CU, Srinivas PN, Mohindra KS. The Health of Indigenous Populations in South Asia : A Critical Review in a Critical Time. 2022;

20. Akter S, Davies K, Rich JL, Inder KJ. Barriers to accessing maternal health care services in the Chittagong Hill Tracts, Bangladesh: A qualitative descriptive study of Indigenous women's experiences. PLoS One. 2020;15(8):e0237002.

21. Islam RM. Utilization of maternal health care services among indigenous women in Bangladesh: A study on the Mru tribe. Women Health. 2017;57(1):108–18.

22. Chowdhury SK. Uptake of antepartum care services in a matrilineal-matrilocal society: a study of Garo indigenous women in Bangladesh. BMC Pregnancy Childbirth. 2023;23(1):75.

23. Akter S, Davies K, Rich JL, Inder KJ. Community perspectives of barriers indigenous women face in accessing maternal health care services in the Chittagong Hill Tracts, Bangladesh. Ethn Health. 2022;27(5):1222–40.

24. Statistics S. Kaveri Mayra 1 , Zoë Matthews 1 , Sabu S Padmadas 1 1. :1–39.

25. Khosla R, Zampas C, Vogel JP, Bohren MA, Roseman M, Erdman JN. International human rights and the mistreatment of women during childbirth. Health Hum Rights. 2016;18(2):131.

26. Das DrTK. Psycho-Social dimensions of ethnicity : The situation of the Garo community in Bangladesh. Journal of Ethnic Affairs [Internet]. 2005;I(1):5–10. Available from: www.ecdo-bd.org

27. Islam MR, Islam MA, Banowary B. Determinants of exposure to mass media family planning messages among indigenous people in Bangladesh: A study on the Garo. J Biosoc Sci. 2009;41(2):221–9.

28. Rahman A. Fighting Exclusion: Towards Understanding the Predicament of Adivasis in Bangladesh. In: Political Governance and Minority Rights. Routledge India; 2020. p. 104–20.

29. Gerharz E. Indigenous activism in Bangladesh: translocal spaces and shifting constellations of belonging. Asian Ethnicity. 2014;15(4):552–70.

30. Bal E, Chambugong TC. The Borders that Divide, the Borders that Unite: (Re)interpreting Garo Processes of Identification in India and Bangladesh. J Borderl Stud. 2014;29(1):95–109.

31. Le Roux J, Oyedemi TD. Indelible apartheid: intergenerational post-colonial narratives of colonial-born coloured females about hair, race and identity in South Africa. Social Identities. 2022;28(2):150–65.

32. Raitapuro M, Bal E. 'Talking about mobility': Garos aspiring migration and mobility in an 'insecure' Bangladesh. South Asian Hist Cult [Internet]. 2016;7(4):386–400. Available from: http://dx.doi.org/10.1080/19472498.2016.1223723

33. Sumon MH. Ethnicity, Ethnographic Imagination, and Identity Construction of Mandi Youth in Dhaka.

34. Navile TE, Hossain J. BIRTH RITUAL, PRACTICES AND GENDER PREFERENCE FOR EXPECTING CHILD IN A MATRILINEAL INDIGENOUS COMMUNITY: A CASE OF GAROS LIVING IN SAINAMARI VILLAGE, MADHUPUR, BANGLADESH. Journal of the Asiatic Society of Bangladesh (Hum). 2019;64(2):239–50.

35. Bhowmik KR, Das S, Islam MA. Modelling the number of antenatal care visits in Bangladesh to determine the risk factors for reduced antenatal care attendance. PLoS One. 2020;15(1):e0228215.

36. Ame AS, Mozumdar L, Islam MA. Impact of social networks on the choice of place of delivery among ethnic women in Bangladesh. Sexual & Reproductive Healthcare. 2021;28:100588.

37. Akter S. Examining access to health services for women of reproductive age from ethnic communities in Chittagong Hill Tracts (CHT) of Bangladesh.

38. Bhopal R. Glossary of terms relating to ethnicity and race: for reflection and debate. J Epidemiol Community Health (1978). 2004;58(6):441–5.

39. Callaghan WM. Maternal mortality: addressing disparities and measuring what we value. Vol. 135, Obstetrics & Gynecology. LWW; 2020. p. 274–5.

40. Janevic T, Sripad P, Bradley E, Dimitrievska V. “ There’s no kind of respect here” A qualitative study of racism and access to maternal health care among Romani women in the Balkans. Int J Equity Health. 2011;10(1):1–12.

41. Jee-Lyn García J, Sharif MZ. Black lives matter: A commentary on racism and public health. Am J Public Health. 2015;105(8):e27–30.

42. Dörr NM, Dietz G. Racism against Totonaco women in Veracruz: Intercultural competences for health professionals are necessary. PLoS One. 2020;15(1):e0227149.

43. Bailey ZD, Feldman JM, Bassett MT. How structural racism works—racist policies as a root cause of US racial health inequities. Vol. 384, New England Journal of Medicine. Mass Medical Soc; 2021. p. 768–73.

44. Timmins CL. The impact of language barriers on the health care of Latinos in the United States: a review of the literature and guidelines for practice. J Midwifery Womens Health. 2002;47(2):80–96.

45. Akinade T, Kheyfets A, Piverger N, Layne TM, Howell EA, Janevic T. The influence of racial-ethnic discrimination on women’s health care outcomes: A mixed methods systematic review. Soc Sci Med. 2023;316:114983.

46. Crenshaw K. Demarginalizing the intersection of race and sex: A black feminist critique of antidiscrimination doctrine, feminist theory and antiracist politics. u Chi Legal f. 1989;139.

47. Echebarria-Echabe A. Socio-Psychological Approaches to Racism: A Critical Review. Papers on Social Representations Peer Reviewed Online Journal ISSN. 1997;6(1):1–11.

48. Hagendoom L, Hraba J. Social distance toward Holland’s minorities: Discrimination against and among ethnic outgroups. Ethn Racial Stud. 1987;10(3):317–33.

49. Stikkers K. ... But I'm Not Racist": Toward a Pragmatic Conception of "Racism. The Pluralist. 2014;9(3):1–17.

50. Carignan N, Sanders M, Pourdavood RG. Racism and Ethnocentrism: Social Representations of Preservice Teachers in the Context of Multi- and Intercultural Education. Int J Qual Methods. 2005;4(3):1–19.

51. Baraldi C. New forms of intercultural communication in a globalized world. Int Commun Gaz. 2006;68(1):53–69.

52. POVERTY AND SUSTAINABLE LIVELIHOODS IN THE SEASONALLY SUBMERGED HAOR AREA OF NETROKONA DISTRICT , BANGLADESH TALUKDER GOLAM RABBY THESIS SUBMITTED IN FULFILMENT OF THE REQUIREMENTS FOR THE DEGREE OF DOCTOR OF PHILOSOPHY FACULTY OF ECONOMICS AND ADMINISTRA. 2012;

53. Karim MA, Kabir MM, Siddiqui MA, Laskar MSI, Saha A, Naher S. Epidemiology of Imported Malaria in Netrokona District of Bangladesh 2013-2018: Analysis of Surveillance Data. Malar Res Treat. 2019;2019.

54. Sharmin S. Socio-Economic Situation and Land Rights of the Indigenous People in Bangladesh. OIDA International Journal of Sustainable Development. 2011;2(11):85–96.

55. Rahman SMB, Uddin MB, Hussain I. Anthropometric study on children of Garo and non-Garo families in Netrakona district of Bangladesh. Journal of the Bangladesh Agricultural University. 2011;9(2):267–72.

56. Rahman M. Paradoxes of participation of the women leaders in the union parishad in Bangladesh: A phenomenological analysis. Jurnalul Practicilor Comunitare Pozitive. 2019;19(2):58–73.

57. Merz S, Jaehn P, Pischon T, Fischer B, Wirkner K, Rach S. Investigating people ' s attitudes towards participating in longitudinal health research : an intersectionality - informed perspective. 2023;1–18.

58. Etikan I, Alkassim R, Abubakar S. Comparision of snowball sampling and sequential sampling technique. Biom Biostat Int J. 2016;3(1):55.

59. Atkinson R, Flint J. Accessing hidden and hard-to-reach populations: Snowball research strategies. Social research update. 2001;33(1):1–4.

60. Klar S, Leeper TJ. Identities and intersectionality: a case for Purposive sampling in Survey-Experimental research. Experimental methods in survey research: Techniques that combine random sampling with random assignment. 2019;419–33.

61. Roy P, Promila M. Quest for security, equality, equity and integration: Locus of Indigenous women in Bangladesh. Prof Chowdhury Mong Shanoo, editor Thailand: Asia Indigenous Peoples Pact (AIPP) and Kapaeeng Foundation. 2014;99–118.

62. Vinding D, Kampbel ER. Indigenous Women Workers With case studies from Bangladesh, Nepal and the Americas . ILO’s International Labour Standards Department (PRO 169) . 2012. 1–58 p.

63. Beckett K, Hoffman B. Challenging medicine: Law, resistance, and the cultural politics of childbirth. Law Soc Rev. 2005;39(1):125–70.

64. Felton-Busch C, Larkins S. Remote dwelling Aboriginal Australian women and birthing: A critical review of literature. Women and Birth. 2019;32(1):6–15.

65. Bar-Zeev SJ, Barclay L, Farrington C, Kildea S. From hospital to home: the quality and safety of a postnatal discharge system used for remote dwelling Aboriginal mothers and infants in the top end of Australia. Midwifery. 2012;28(3):366–73.

66. Cidro J, Hayward A, Bach R, Sinclair S. Walking with our Most sacred: indigenous birth workers clearing the path for returning birthing to indigenous communities. Introduction to Determinants of First Nations, Inuit, and Métis Peoples’ Health in Canada. 2022;225–36.

67. Yeung S. Conceptualizing cultural safety: Definitions and applications of safety in health care for Indigenous mothers in Canada. Journal for Social Thought. 2016;1.

68. Hayward A, Cidro J. Indigenous birth as ceremony and a human right. Health Hum Rights. 2021;23(1):213.

69. Akter S. Factors influencing health service utilization among mothers for under-five children: A cross-sectional study in Khulna district of Bangladesh. PLoS One. 2022;17(9):e0274449.

70. Moran A. White Australia, settler nationalism and Aboriginal assimilation. Australian Journal of Politics and History. 2005;51(2):168–93.

71. Butler R, Ben J. Centring settler colonialism in rural Australian multicultures: race, place and local identities. J Ethn Migr Stud. 2021;47(9):2179–97.

72. Dietz G, Mateos Cortés LS. Multiculturalism and Intercultural Education Facing the Anthropology of Education. A Companion to the Anthropology of Education. 2011;(July):495–516.

73. Celis K, Erzeel S, Mügge L, Damstra A. Quotas and intersectionality: Ethnicity and gender in candidate selection. International Political Science Review. 2014;35(1):41–54.

74. Spivak G. In Other Worlds: Essays in Cultural Politics. New York: Methuen. other worlds: essays in cultural politics. 1987;

75. Sauerbronn FF, Ayres RM, da Silva CM, Lourenço RL. Decolonial studies in accounting? Emerging contributions from Latin America. Critical Perspectives on Accounting. 2021;(xxxx):102281.

Click here to access/download
Supporting Information
Ethics Approval Letter.pdf

Click here to access/download
Supporting Information
In-depth Interview checklists.docx

"They don't even listen to our voices": Ethnocentrism and biomedical maternity care access among Garo indigenous women in Bangladesh

Suban Kumar Chowdhury[1]

[1] Department of International Relations, University of Rajshahi, Bangladesh.

[*]Suban Kumar Chowdhury

E-mail: skc_ruir@ru.ac.bd

Author's contributions

The author developed the idea of conducting this research from his interest in indigenous women's maternal health care matters. The author reviewed the relevant literature and designed the research instruments to complete this study. The author wrote the initial and final drafts of the manuscript as the principal researcher. For data collection, the author sought help from four female research assistants appointed from the Garo community.

Acknowledgement

The author is thankful to the anonymous reviewers of this manuscript for their kind review and valuable comments. This article is part of the author's 'MSc thesis work' submitted to AIT under Dr Joyee S Chatterjee (program Chair-Gender and Development Studies program- and Assistant Professor at the Department of Development and Sustainability, AIT). Therefore, the author would like to thank Dr Joyee S Chatterjee. Also, the author would like to thank the research participants and the three research assistants (who helped throughout the data collection process).

Abstract

Garo, a matriarchal ethnic group in Bangladesh, faces discriminatory health care treatment during pregnancy and childbirth. This study examined how ethnocentrism affects this group of women's experience accessing biomedical maternity care. It did this by using Janevic's conceptual framework from the analytical lens of intersectionality, which helped create the analytical categories. The study followed a qualitative research approach. 24 semi-structured interviews were conducted with Garo expectant mothers with the help of three female research assistants appointed from the Garo community. The interview sessions were mostly focused on knowing about biomedical care seeking during pregnancy and childbirth, the previous or shared experience of biomedical care, and the perception of ethnocentric discrimination in biomedical maternity care. The audio-recorded data were transcribed into the Bengali language first with the help of the research assistant and then transcribed into English. The emerging themes were grouped under institutional, personally-mediated, and internalized ethnocentrism. The emergent themes include inadequate material conditions, absence of culturally competent care, and power dynamics at the institutional level of ethnocentrism, interpersonal hierarchy, language barrier, use of non-sensical medical jargon, and community practices of maternity care and miscommunication at the level of personally-mediated ethnocentrism, and the themes emerged under the domain of internalized ethnocentrism includes less agency, self-devaluation, and unworthiness of biomedical care. These themes originated from a shared understanding and experience of hospital-level treatment. The three levels of ethnocentrism intersect and produce gendered power structures, masculinist attitudes, prejudice, and stigmatization, making it difficult for Garo mothers to access maternity care. The author proposes a clinician intercultural competency training program (CICTP), and

institutional-level initiatives are needed to improve hospital staffing and provide culturally competent care.

Introduction

Ethnocentrism-refers to cultural stereotypes, prejudice, discrimination, and an unwillingness to learn from other cultures (1)-is a major maternity health concern (2). However, how it impacts indigenous women's experience accessing maternity care services is often overlooked (3). This is crucial because research conducted in different countries has shown that indigenous women experience higher maternal and infant morbidity rates and mortality linked to ethnocentrism (4–7), (8). For example, healthcare providers' ethnocentric discrimination, which refers to the unjust treatment of individuals or groups based on their ethnic or cultural background (9), in maternity care against indigenous mothers is a well-documented issue in the United States of America and India (10–15). Recent research showed that ethnocentric discrimination and cultural insensitivity in the biomedical maternity care system could lead to feelings of disempowerment, mistrust, and trauma, leading to little interest in healthcare access. It is an important argument for understanding the significance of ethnocentrism as a social determinant of health (16).

Ethnocentrism, however, is a neglected health issue in Bangladesh and other South Asian nations (17). A comprehensive review of indigenous South Asian women's maternal health found that ethnicity affects maternity healthcare access, including varied health conditions and life expectancy, limited biomedical care services, unfriendly hospital staff, inequitable treatment and bias, dissatisfaction, and lack of practical and emotional support (18–20). However, ethnocentrism and maternity care access in Bangladesh are still not being studied, except for a few studies that have examined the experiences of indigenous moms who receive maternity care (21–23) even though research is increasing worldwide, with studies from the United States (4), Mexico (8), and India (24). Given the evidence that a woman's social

setting caused variation in the experience of maternity healthcare experience (25), a context-specific understanding of ethnocentrism and maternity healthcare access is crucial.

Given the above landscapes, this study examined how ethnocentrism affects Garo indigenous women's experience of biomedical maternity care in Bangladesh. Since 2011, ethnic prejudice against the Garo, one of the world's last close-knit matriarchal communities (26–28), become more due to their recognition as tribal, meaning uncivilized people (29, 30). Such designation implied ethnic inferiority and continued colonial exclusionary tactics (31). Linking to discriminatory postcolonial policies concerning the case of the Garo, a group of scholars claim that the state has overlooked the Garo and other ethnic minority groups in Bangladesh their right to be recognized as indigenous communities and has reified and essentialized their culture and traditions (32). Institutional discourses have portrayed their culture as something to be kept in galleries, separating 'indigenous' people from 'civilization' and linking them to the past (33). In addition, statistics show 62.3% of Garo women did not receive skilled maternity care (34–36). Despite Garo women's limited access to biomedical maternity care, ethnic prejudice has not been studied in this context. The Bangladeshi government's commitment to indigenous-friendly health care requires disaggregated data on indigenous mothers' health, which is lacking (37). Thus, it adds another dimension to undertaking this study. Another rationality lies in previous research findings showing that women's vulnerability to maternal health owing to ethnicity is in the limelight, and maternity care is under question (38,39).

The current study used a framework adapted from Janevic's conceptual underpinning of racism and, thus, framed ethnocentrism under three levels: institutional, personally-mediated, and internalized (40). Institutional-level ethnocentrism is the routine and systemic enactment of ethnocentric discrimination by institutions, policies, and practices that deeply and extensively affect underprivileged indigenous women (41). Thus, institutionalized ethnocentrism occurs via commission (activity, habit, social practice, legislation) and omission, manifesting in material condition and power dynamics (42). Personally-mediated ethnocentrism is related to individual action of treating others in a discriminatory way based on ethnicity

and manifests due to disrespectfulness, distrust, devaluation, blaming, and dehumanization (40,43,44). Internalized ethnocentrism refers to the acceptance of own abilities and intrinsic worth by stigmatized ethnic individuals, leading to resignation, helplessness, and hopelessness (40).

Janevic's conceptual framework has been widely used to study indigenous women's discriminatory maternal healthcare access. Most studies have focused on racism (45), ignoring ethnocentrism from an intersectional analytical approach examining how interlocking power systems affect underprivileged women (46). This is crucial because ethnocentrism, the root of racism (47), has distinct social representations (47) (48). Racism is a social construct of attitudes, views, opinions, behavior, practices, and actions (49)(50), while ethnocentrism is how someone sees the world through their meanings (51). Thus, understanding the indigenous mothers' experience of accessing biomedical maternity care requires a more holistic approach emphasizing the intersectionality of institutional, personally-mediated, and internalized ethnocentrism. This study used Janevic's conceptual framework to fill this gap.

Materials and methods

Settings

Kalmakanda is one of 10 administrative Upazila (sub-district) of Netrakona District in northern Bangladesh (52). Out of the 8 unions (village), Lengura and Nazirpur are the two unions that have long been home to most of the Garo people of Bangladesh (53)(54). The study was conducted at these two unions on the northeastern side of Kalmakanda Upazila, with scant maternal healthcare facilities (55,56). The Kalmakanda Hospital has two Union sub-centres and 31 community clinics, but these services are unavailable in either Union. The Upazila hospital has ten doctors, eleven nurses, two male medical assistants, and a cesarean section operated by a non-Garo man gynecologist. Most of the pregnant patients who needed care were not Garo mothers. It means mothers' health is a top concern in the hospital, but not many Garo women use these services.

Map 1: Study area

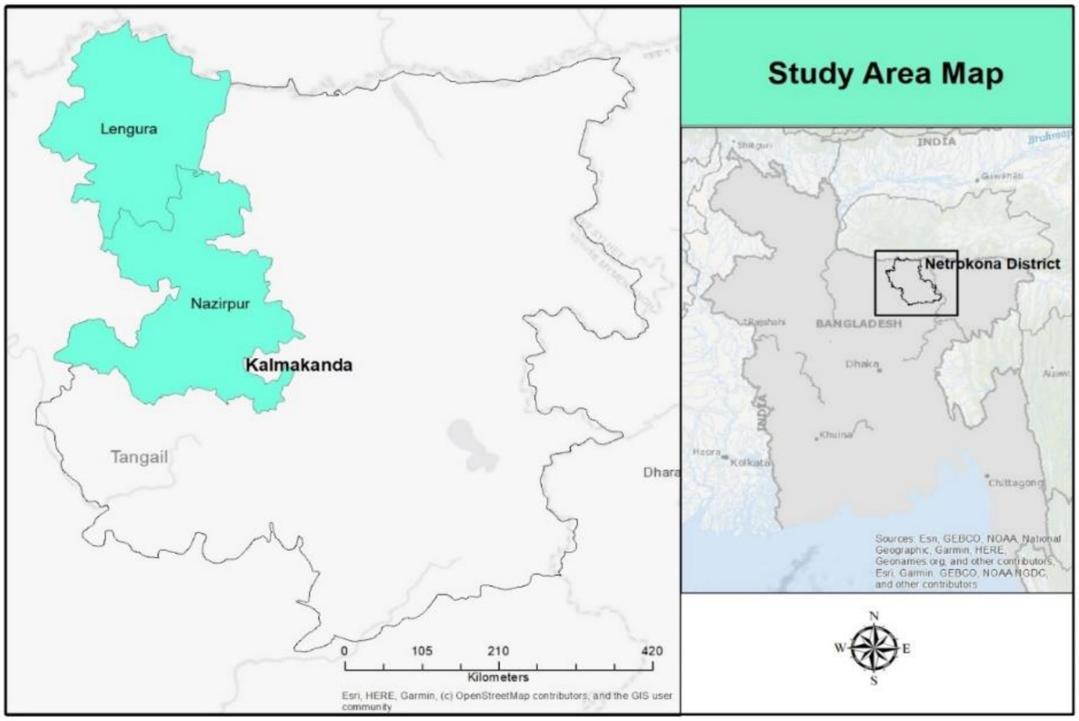

Study Procedures

This qualitative study conducted in-depth interviews with Garo women to understand their perspectives, emphasizing an effort to listen to their unheard voices. The interviews were conducted with assistance from three Garo female research assistants living in the research area.

Sampling and recruitment

Participants were selected using “intersectionality-informed methods”. Such techniques involved “thinking beyond categories like gender, age, education level, and economic class, as additives or alone, create space for the complexity of identities and experiences” (57). It helps to see identities and experiences as multidimensional rather than additive or distinct (46). Thus, the author interviewed 24 Garo moms using purposive sampling (58), specifically "snowball sampling" (59) as an intersectionality-informed method (60). Snowball sampling was utilized since identifying

pregnant and new mothers was difficult. A recruiting matrix was used to ensure age, literacy, and pregnancy diversity.

Data collection

The researcher conducted in-depth interviews with participants from February 2021 to March 2021, using semi-structured interviews and open-ended questionnaires. The in-depth interview questionnaire covered knowledge of biomedical maternal health care, maternal health beliefs, and pregnancy and childbirth health care experiences. Training sessions were organized for research assistants, and checklists were prepared. Each interview lasted at least 50 minutes and was audio-recorded and saved on a password-protected computer. Field notes were also taken.

Analysis

The research assistants first transcribed the audio-recorded data into Bengali because the data were taken in the Mandi language. Then, the author translates these into English. Next, the author analyzed the transcribed data, written in English, using constant comparison and displayed verbatim. After that, the transcripts were coded in four phases: initial codes, refinements, drawing a diagram to structure the codes, and categorizing the themes that indicate the three levels of ethnocentrism's effect on Garo women's access to biomedical maternal care. In this process, the researcher rechecked the codes and themes to ensure that these best reflect the Garo mothers' perspectives.

Ethics

This study is part of the author's MSc thesis at the Asian Institute of Technology (AIT), Thailand. The author received ethics approval from AIT Ethics Committee. Before conducting interviews, participants and their husbands were contacted and informed about the research. Also, verbal

consent was taken before conducting interviews, and the ethics committee approved it. The participants were informed that confidentiality would be maintained and their participation was voluntary and could be withdrawn without consequences. Pseudonyms were used to ensure data privacy.

Results

Participant characteristics

The in-depth interview (IDI) included 24 Garo mothers. **Table 1** lists the number of IDI participants and the inclusion criteria. After nineteen IDI saturation of themes was achieved. Five more IDI were conducted to confirm the saturation of themes and execute an in-depth exploration of some subthemes. The author noticed active engagement of all participants with good interaction with himself and the research assistants.

Table 1: Lists of IDI and KII respondents and their inclusion criteria.

IDI respondents				
Area			***Number***	***Selection criteria***
Netrokona District	Kalmakanda Upazila	Lengura Union	n=12	The Garo women (aged 18 to 35) who were pregnant or had childbirth within a year on the interview date.
		Nazirpur Union	n=12	

Figure 1 visualizes the eight themes from coding. Following Janevic's conceptual framework, these are organized into three levels for clarity. Themes under the institutional level include

material conditions, the absence of culturally appropriate treatment, and power dynamics. Inter-personal hierarchy, language barrier, non-sensical medical terminologies, and community practices of maternity care and misunderstanding are grouped under personally-mediated ethnocentrism. The third level, internalized ethnocentrism, includes less agency and self-devaluation. Based on Figure 1, as visualized below, the following sub-section presents the themes in detail.

Figure 1: Three layered ethnocentrism and the emergent themes

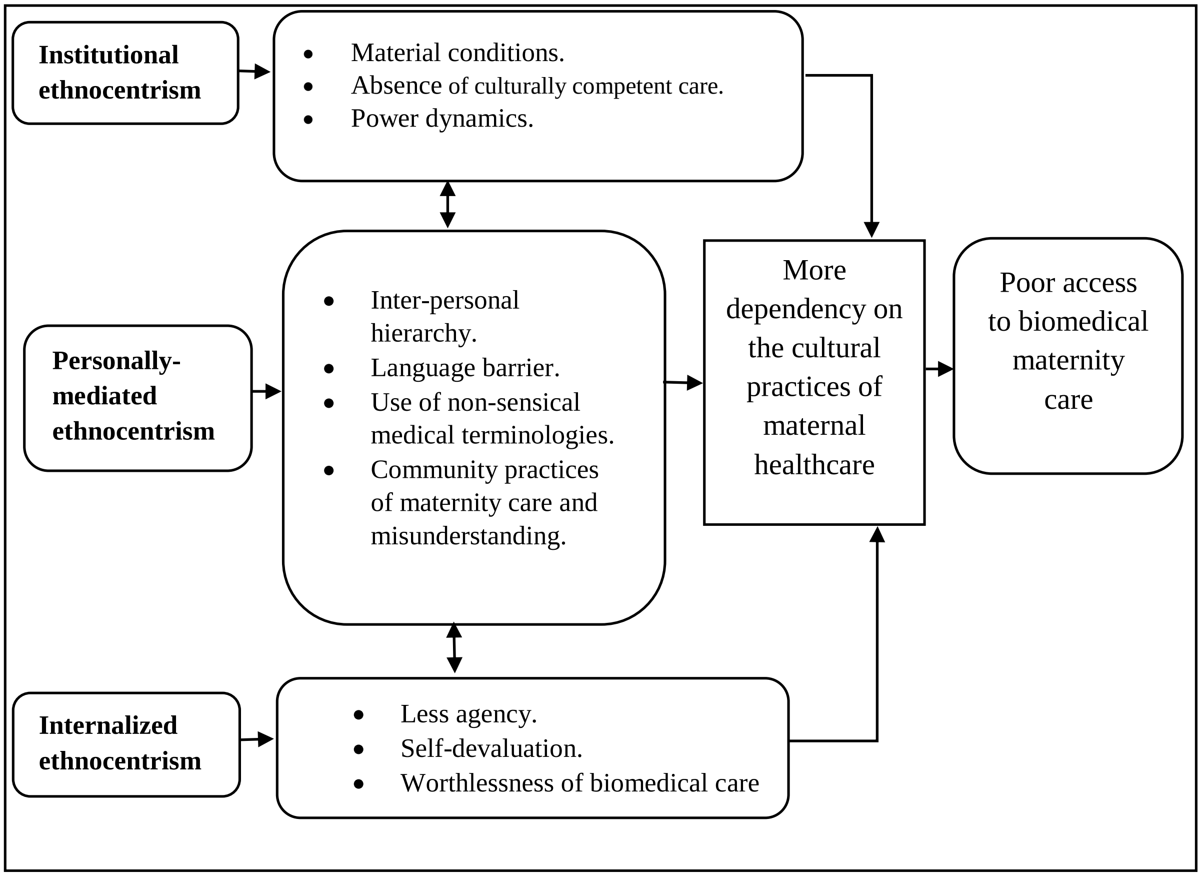

Institutional ethnocentrism

Material conditions and power dynamics represent institutional ethnocentrism. Indigenous women in Bangladesh are marginalized and lack institutional biomedical care (61). This study confirmed

this trend. Mothers often blame poor maternity care access on inadequate service facilities and institutional power dynamics. One mother reported:

> The hospital has no services, making pregnancy care impossible. We have trouble getting care because the only available doctor always found busy with non-Garo women. The man gaini doctor (gynecologist) always serves non-Garo women, ignoring us. What can I do there? I don't go to hospitals like my neighbors (L8, 26).

The interviewed women cited inadequate hospital staffing, discriminatory treatment, and clinicians' priority of caring for local women for not using institutional health facilities. The Garo mothers' accounts also suggest culturally inappropriate birthing care. Cultural issues were most apparent in the hospital's dressing rules and birthing room privacy and the absence of Garo female clinicians. Furthermore, they complained not meeting the midwife before hospital birth. She said:

> Doctors are not Garo women. No one is allowed to enter the delivery room without wearing the hospital's dress provided by them (the doctors). It's unfair because many women use these clothes, already, during their delivery (N6, age 28).

In addition, some mothers highlight the higher cost of seeking care from the Upazila hospital as another reason for not attending institutional care:

> Distance cost isn't all. Hospitals want money. Despite their claim, they don't charge 5 BDT. They demand more. Traveling makes you hungry, but food is expensive. We must spend all we earn if we go. She also adds, we don't control travel and treatment costs. They ignore our issue. They said "you

> have to follow the hospital rules" whenever we told them our problem. What else can we do but avoid it? (L2, age 30).

Due to expensive travel and treatment, Garo mothers avoid biomedical care. These factors must be taken into account when managing maternity health care. It is an example of typical financial cost-driven discrimination in biomedical maternity care. A study found that indigenous women in Bangladesh earn less due to ethnicity (62). Hence, the Garo mothers' denial of biomedical care due to economic cost-driven discrimination shows how institutionalized ethnocentrism affects biomedical care access. Overall, the mothers typically blamed the insufficient human resources, the power of the clinicians for poor access. Also, they present the obligation of following the institutional guidelines defined by the clinicians as a reason for not accessing these services. This illustrates a link to the personally-mediated ethnocentrism, as presented in the following sub-section.

Personally-mediated ethnocentrism

The Garo mothers' experiences with clinicians' personally-mediated ethnocentrism include disrespect, distrust, devaluation, and blaming indigenous cultural practices. Another issue that emerged is the clinicians' ethnocentrism related to language, which made them view Garo mothers' Mandi language use as problematic. These women generally described how a clinician miscommunicated with them, showing that indigenous language speakers are discriminated against because they do not communicate using mainstream language:

> He (the clinician) doesn't understand what we're saying. We don't want to be treated by him because he's aggressive and doesn't know how. Our Garo women went there with health issues like jak jachak dalya (swelling of feet and hands), wakkalna ha'sika (vomiting), matha betha (dazziness), Hapani

> (shortness of breath), and bikma chikki sadika (abdominal pain), but the doctor didn't listen. He gives us medicine even though he doesn't understand (N5, age 24).

Few Garo mothers were bilingual; they speak Mandi in the community and Bengali elsewhere. Their accounts suggest they still face discrimination at the Upazila Hospital due to their difficulty pronouncing Bengali words. Data demonstrates that using Bengali in the hospital is emotionally and substantially harder than in daily life. The women also complained about clinicians' using nonsensical medical terminology they were unfamiliar with. As one woman typically reported:

> Though I can't pronounce Bengali words well, I can explain my health issues to doctors. But, I'm hesitant to go again because the doctor laughed at me when I talked about my health issues a few months ago. The doctor replied with medical jargon. He laughed again when I asked him to explain these. I felt insulted (L7, age 27).

The issue of power and domination over the Garo mothers' bodies was highlighted, with the use of force by clinicians, touching private organ without permission, and not giving the placenta to the family to perform cultural rituals as significant barriers. As one mother reported:

> Our culture forbids delivering babies by cutting the belly. They touch our private organ during mid-pregnancy visits. Permission was never sought. My Garo neighbors say that. I won't go. They forcefully violate our culture (N2, age-23).

The woman's quote suggests that clinicians used masculinist power to disregard the mothers' cultural norms for maternity care and forced them to give up their cultural practices to promote biomedical care as the embodiment of modernity. The women's accounts also showed that

clinicians' perception about community maternal health care practices stigmatized them, making it difficult for them to get to Upazila Hospital:

> The doctors don't listen to our cultural needs, instead blaming us for our customs, such as not cutting the belly and reciting the delivery mantra. They said we are responsible for our health complications, especially birthing complications (N5, age 24).

According to mothers, clinicians stigmatize them for respecting indigenous maternity care customs. Most women were afraid to go to a hospital because they thought the clinicians would force C-sections or vaginal incisions, which violate cultural norms and harm reproductive health. Thus, the clinicians' personally-mediated ethnocentrism stigmatized Garo mothers by attributing serious maternal health complications to the community's maternity healthcare practices. As data suggests, the goal is to place mothers under biomedical supervision without regard to indigenous culture. Thus, the doctor-Garo mother hierarchy is revealed:

> We were forced to cut the belly for delivering the baby. That's why we won't go to the hospital. In fact, the doctor decides how to deliver, not we (N9, age 26).

As evident from the data, the clinicians' suggestion for C-section to Garo mothers reflects institutionalized ethnocentrism, which ignores the culturally believed health safety of normal birthing. This suggests the interplay between institutional and personally-mediated ethnocentrism, making the Garo mother hesitant to access biomedical maternity care.

Internalized ethnocentrism

Three sub-themes emerged related to internalized ethnocentrism. There are less agency, self-devaluation, and worthlessness in biomedical care. While discussing the effect of the previous experience of being stigmatized, another mother typically reported her less agency in raising voices concerning the perceived and shared negative experiences of ethnocentric discrimination:

> I was embarrassed and disappointed when the doctor told me my cultural beliefs were to blame for my health issues. Since I'm Garo and there's no Garo doctor, I can't comment on the doctor's behavior (N5, age 24).

Self-devaluation comes from the fact that women often blame themselves for how health professionals treat them when they seek medical care:

> We are responsible for how the doctors treat us in the hospital, as we don't understand their language, speak Bengali with them, and dress like them. It's all our fault (N3, age 19).

Interestingly, the interviewed mothers linked their less agency and feeling of self-devaluation with the worthlessness of biomedical care compared to community maternity care. Evidence suggests that their encounters with discrimination and stigmatization at institutional ethnocentrism manifested in material conditions and power dynamics and at the level of personally-mediated ethnocentrism reflected in hierarchical relations. Some studies showed the hegemony of biomedical treatment, defined as the domination of medical care (63), over traditional cures (64)(65), while another found that biomedical care has replaced indigenous mothers' community cultural practices of maternity care (66). This study contradicts these findings, as institutionalized ethnocentrism and clinicians' personally mediated ethnocentrism manifested in hierarchical power

relations and stigmatization strengthen these mothers' dependence on community-based maternity care over biomedical care:

> It is better to seek care at home following our cultural practices, as there is no chance of being insulted by the doctors (L7, age 27).

Thus, it seems that due to the internalization of the intersectional mix of institutional and personally-mediated ethnocentrism which the health institutions imposed on them and which they reproduce by identifying with the dominant biomedical facilities.

Discussions

This study used Janevic's conceptual framing (40) to explore the Garo indigenous mothers' biomedical maternity care experiences. Results showed that Garo women were hesitant to get biomedical care due to inadequate material conditions, human resources, discrimination based on economic status, ethnicity, culturally inappropriate care, barriers related to language, and access to information. A previous study has focused on biomedical care service delivery institutions' neglect of ethnocentric discrimination (17). Data demonstrates that these factors are the dimensions of institutionalized ethnocentrism manifested in ethnocentric practices that create power imbalances between the hospital staff and the Garo mothers. These outcomes led to self-low-agency, self-devaluation, and dependency on community maternity care considering biomedical care as worthless.

The methodological limitations of the current study were driven by the participants' varied traits and emotional responses. By fostering trust, the author was able to get past this limitation. This trust was confirmed when the Garo indigenous mothers refused to talk about their experiences publicly but did so during individual interviews in their homes. The analysis is based on the data

found, but triangulation with health professionals and mainstream women's experiences may contribute much more to research outcomes if contrast is done among these three perspectives.

Ethnocentrism levels often reinforce each other, according to analysis. The hospital's non-indigenous staff's interactions with the Garo indigenous mothers demonstrate a post-colonial mindset and asymmetric power relations. Previous studies in different countries have shown the continuity and persistence of imbalanced power relations between biomedical caregiving institutions and indigenous mothers and their cultural healing related to maternity care (67, 68) (42). Such a pattern also becomes evident in the current study. Bangladesh's health policy segregated the indigenous people from the mainstream population by acknowledging them as a tribe, meaning uncivilized (29), but not as Adivasi (indigenous community) (69). It prevents health practitioners from understanding historical and contemporary contexts and identifying unequal power relations with Garo mothers. Thus, as data suggests, health practitioners following service guidelines reproduce colonial prejudices and stereotypes in an institutionalized context. Resultantly, Garo indigenous patients have found internalized a sense of low agency and self-dehumanization and considered biomedical care unworthy because it undermined cultural maternity care practices. The mothers' strong belief in their culture, close-knit community, and neighborhood settings explain the unworthiness. This is unlike a previous study conducted in the context of Mexico that found mothers abandoning indigenous cultural practices and assimilation with the biomedical care system in the name of modernization (42).

Concerning modernization, other studies have shown that segregationist colonialism and assimilationist nationalism have shaped public institutions' relations with indigenous people

(70)(71). Thus, it creates a racialized "grammar of diversity," an underlying cultural logic of intercultural relations between state representatives and communities (72). Adding to these findings, the present study shows that ethnocentrism interacts with other sources of discrimination (46). For example, as data suggests, two sources are the gendered power hierarchies and clinicians' masculinist attitudes toward Garo mothers, especially in community maternity care and interpersonal interactions. Thus, this finding provides another layer of contribution to a recent study (45).

In the research site, the hospital staff wielded gendered power and saw Garo mothers as responsible for their maternal health complications, wrongfully dependent on their community maternity care practices. Gendered power hierarchy is reflected at the level of personally-mediated ethnocentrism and reproduced masculinist behavior among clinicians towards Garo mothers, causing discrimination, language barriers, miscommunication, and misunderstanding between doctors and Garo mothers about community maternity care and biomedical care. However, institutionalized ethnocentrism underlies this power hierarchy and masculinist attitudes, suggesting its cross-cutting relationship with personally-mediated ethnocentrism. Thus, institutional and personally-mediated ethnocentrism revealed gendered power hierarchies and masculinist attitudes toward indigenous Garo mothers and their maternity care. Based on this finding, the current study suggests further research from the feminist analytical lens of power to understand how gender, ethnicity, and the three levels of ethnocentrism affect Indigenous mothers' access to biomedical maternity care. Because ethnicity and gender are mutually constitutive of ethnocentric discrimination (73), such a future study would be reasonable. However, the study showed that three levels of ethnocentrism intersected and cross-cut, creating a gendered power

hierarchy and masculinist attitudes among clinicians that discriminated against Garo mothers. The clinicians' personally mediated ethnocentrism intertwined with historical colonialism through the subalterization of Garo indigenous mothers, who were perceived as the "others" by doctors. Subalterization, which devalues women and their culture based on their social position across multiple identities, including ethnicity (74), drives colonization (75).

This study emphasized the importance of clinicians' intercultural competency training program (CICTP) that promotes ethnic relations and an institutional policy that increases hospital staffing and culturally sensitive maternity care. The CICTP would eliminate hierarchical relations between health practitioners and indigenous mothers, bias and discrimination against indigenous mothers, miscommunication, and misunderstanding about cultural birthing norms and placenta practices. Indigenous mothers may overcome low agency and self-doubt if clinicians recognize cultural competencies in maternity care and non-discrimination. Indigenous mothers must be convinced that biomedical and community maternity care can improve maternity health together to change their view of biomedical care as worthless.

Conclusion

Ethnocentrism is an important maternity health concern for Garo indigenous women, with institutional ethnocentrism and personally-mediated ethnocentrism manifesting in discrimination and stigmatization due to language and community practices. In this study, data demonstrates internalized ethnocentrism in two different directions. First, a feeling of low agency and self-devaluation, and second, a perception of the worthlessness of biomedical care. Therefore, to overcome ethnocentric discrimination and stigmatization, the study suggests that CICTP training programs and institutional initiatives related to increasing the number of hospital staffing and

providing culturally competent care are strikingly necessary. Such training does not to substitute any existing training program organized for the clinicians, but to inject a strong sense of ethnic-relations to ensuring the delivery of culturally appropriate care acknowledging the ethnic diversity.

References

1. Judd CM, Park B, Ryan CS, Brauer M, Kraus S. Stereotypes and Ethnocentrism: Diverging Interethnic Perceptions of African American and White American Youth. J Pers Soc Psychol. 1995;69(3):460–81.

2. Hine R, Krakouer J, Elston J, Fredericks B, Hunter SA, Taylor K, et al. Identifying and dismantling racism in Australian perinatal settings: Reframing the narrative from a risk lens to intentionally prioritise connectedness and strengths in providing care to First Nations families. Women and Birth. 2023;36(1):136–40.

3. Jordal M, Wahlberg A. Challenges in providing quality care for women with female genital cutting in Sweden–A literature review. Sexual & reproductive healthcare. 2018;17:91–6.

4. Poetry S. Giving Birth in South Florida : A Phenomenological Study of the Pregnancy and Birthing Experiences among Black and Afro-Latina Women. 2020.

5. Petersen EE, Davis NL, Goodman D, Cox S, Syverson C, Seed K, et al. Racial/ethnic disparities in pregnancy-related deaths—United States, 2007–2016. Morbidity and Mortality Weekly Report. 2019;68(35):762.

6. Hartz D, Sherwood J. Midwives working with Aboriginal and Torres Strait Islander women. Midwifery: Preparation for practice. 2019;158–74.

7. Dossetor PJ, Freeman JM, Thorburn K, Oscar J, Carter M, Jeffery HE, et al. Health services for aboriginal and Torres Strait Islander children in remote Australia: A scoping review. PLOS Global Public Health. 2023;3(2):e0001140.

8. Dörr NM, Dietz G. Racism and discrimination in the care of Totonaca women in Veracruz, Mexico: the need for intercultural competencies. Social Medicine. 2022;15(1):42–53.

9. Shiraev EB, Levy DA. Cross-cultural psychology: Critical thinking and contemporary applications. Routledge; 2020.

10. Vedam S, Stoll K, Taiwo TK, Rubashkin N, Cheyney M, Strauss N, et al. The Giving Voice to Mothers study: inequity and mistreatment during pregnancy and childbirth in the United States. Reprod Health. 2019;16(1):1–18.

11. Thapa R, Teijlingen E Van, Regmi PR, Heaslip V. Caste Exclusion and Health Discrimination in South Asia : A Systematic Review. 2021;

12. Kozhimannil KB, Interrante JD, Tofte AN, Admon LK. Severe maternal morbidity and mortality among indigenous women in the United States. Obstetrics and gynecology. 2020;135(2):294.

13. MacDonald C, Steenbeek A. The impact of colonization and western assimilation on health and wellbeing of Canadian aboriginal people. International Journal of Regional and Local History. 2015;10(1):32–46.

14. Green TL, Zapata JY, Brown HW, Hagiwara N. Rethinking bias to achieve maternal health equity: changing organizations, not just individuals. Obstetrics and gynecology. 2021;137(5):935.

15. Adhikari T, Sahu D, Nair S, Saha KB, Sharma RK, Pandey A. Factors associated with utilization of antenatal care services among tribal women: A study of selected States. Indian J Med Res. 2016;144(1):58.

16. Küey L. Clarifying Definitions of „Race “, Racism, and Ethnocentrism. European Psychiatry. 2022;65(S1):S35–S35.

17. Rice ZS, Liamputtong P. Culture and Global Health: Cultural Determinants of Health and Well-Being. Handbook of Global Health. 2020;1–26.

18. Akter S. Examining access to health services for women of reproductive age from ethnic communities in Chittagong Hill Tracts (CHT) of Bangladesh.

19. Thresia CU, Srinivas PN, Mohindra KS. The Health of Indigenous Populations in South Asia : A Critical Review in a Critical Time. 2022;

20. Akter S, Davies K, Rich JL, Inder KJ. Barriers to accessing maternal health care services in the Chittagong Hill Tracts, Bangladesh: A qualitative descriptive study of Indigenous women’s experiences. PLoS One. 2020;15(8):e0237002.

21. Islam RM. Utilization of maternal health care services among indigenous women in Bangladesh: A study on the Mru tribe. Women Health. 2017;57(1):108–18.

22. Chowdhury SK. Uptake of antepartum care services in a matrilineal-matrilocal society: a study of Garo indigenous women in Bangladesh. BMC Pregnancy Childbirth. 2023;23(1):75.

23. Akter S, Davies K, Rich JL, Inder KJ. Community perspectives of barriers indigenous women face in accessing maternal health care services in the Chittagong Hill Tracts, Bangladesh. Ethn Health. 2022;27(5):1222–40.

24. Statistics S. Kaveri Mayra 1 , Zoë Matthews 1 , Sabu S Padmadas 1 1. :1–39.

25. Khosla R, Zampas C, Vogel JP, Bohren MA, Roseman M, Erdman JN. International human rights and the mistreatment of women during childbirth. Health Hum Rights. 2016;18(2):131.

26. Das DrTK. Psycho-Social dimensions of ethnicity : The situation of the Garo community in Bangladesh. Journal of Ethnic Affairs [Internet]. 2005;I(1):5–10. Available from: www.ecdo-bd.org

27. Islam MR, Islam MA, Banowary B. Determinants of exposure to mass media family planning messages among indigenous people in Bangladesh: A study on the Garo. J Biosoc Sci. 2009;41(2):221–9.

28. Rahman A. Fighting Exclusion: Towards Understanding the Predicament of Adivasis in Bangladesh. In: Political Governance and Minority Rights. Routledge India; 2020. p. 104–20.

29. Gerharz E. Indigenous activism in Bangladesh: translocal spaces and shifting constellations of belonging. Asian Ethnicity. 2014;15(4):552–70.

30. Bal E, Chambugong TC. The Borders that Divide, the Borders that Unite: (Re)interpreting Garo Processes of Identification in India and Bangladesh. J Borderl Stud. 2014;29(1):95–109.

31. Le Roux J, Oyedemi TD. Indelible apartheid: intergenerational post-colonial narratives of colonial-born coloured females about hair, race and identity in South Africa. Social Identities. 2022;28(2):150–65.

32. Raitapuro M, Bal E. 'Talking about mobility': Garos aspiring migration and mobility in an 'insecure' Bangladesh. South Asian Hist Cult [Internet]. 2016;7(4):386–400. Available from: http://dx.doi.org/10.1080/19472498.2016.1223723

33. Sumon MH. Ethnicity, Ethnographic Imagination, and Identity Construction of Mandi Youth in Dhaka.

34. Navile TE, Hossain J. BIRTH RITUAL, PRACTICES AND GENDER PREFERENCE FOR EXPECTING CHILD IN A MATRILINEAL INDIGENOUS COMMUNITY: A CASE OF GAROS LIVING IN SAINAMARI VILLAGE, MADHUPUR, BANGLADESH. Journal of the Asiatic Society of Bangladesh (Hum). 2019;64(2):239–50.

35. Bhowmik KR, Das S, Islam MA. Modelling the number of antenatal care visits in Bangladesh to determine the risk factors for reduced antenatal care attendance. PLoS One. 2020;15(1):e0228215.

36. Ame AS, Mozumdar L, Islam MA. Impact of social networks on the choice of place of delivery among ethnic women in Bangladesh. Sexual & Reproductive Healthcare. 2021;28:100588.

37. Akter S. Examining access to health services for women of reproductive age from ethnic communities in Chittagong Hill Tracts (CHT) of Bangladesh.

38. Bhopal R. Glossary of terms relating to ethnicity and race: for reflection and debate. J Epidemiol Community Health (1978). 2004;58(6):441–5.

39. Callaghan WM. Maternal mortality: addressing disparities and measuring what we value. Vol. 135, Obstetrics & Gynecology. LWW; 2020. p. 274–5.

40. Janevic T, Sripad P, Bradley E, Dimitrievska V. “ There’s no kind of respect here” A qualitative study of racism and access to maternal health care among Romani women in the Balkans. Int J Equity Health. 2011;10(1):1–12.

41. Jee-Lyn García J, Sharif MZ. Black lives matter: A commentary on racism and public health. Am J Public Health. 2015;105(8):e27–30.

42. Dörr NM, Dietz G. Racism against Totonaco women in Veracruz: Intercultural competences for health professionals are necessary. PLoS One. 2020;15(1):e0227149.

43. Bailey ZD, Feldman JM, Bassett MT. How structural racism works—racist policies as a root cause of US racial health inequities. Vol. 384, New England Journal of Medicine. Mass Medical Soc; 2021. p. 768–73.

44. Timmins CL. The impact of language barriers on the health care of Latinos in the United States: a review of the literature and guidelines for practice. J Midwifery Womens Health. 2002;47(2):80–96.

45. Akinade T, Kheyfets A, Piverger N, Layne TM, Howell EA, Janevic T. The influence of racial-ethnic discrimination on women’s health care outcomes: A mixed methods systematic review. Soc Sci Med. 2023;316:114983.

46. Crenshaw K. Demarginalizing the intersection of race and sex: A black feminist critique of antidiscrimination doctrine, feminist theory and antiracist politics. u Chi Legal f. 1989;139.

47. Echebarria-Echabe A. Socio-Psychological Approaches to Racism: A Critical Review. Papers on Social Representations Peer Reviewed Online Journal ISSN. 1997;6(1):1–11.

48. Hagendoom L, Hraba J. Social distance toward Holland’s minorities: Discrimination against and among ethnic outgroups. Ethn Racial Stud. 1987;10(3):317–33.

49. Stikkers K. ... But I'm Not Racist": Toward a Pragmatic Conception of "Racism. The Pluralist. 2014;9(3):1–17.

50. Carignan N, Sanders M, Pourdavood RG. Racism and Ethnocentrism: Social Representations of Preservice Teachers in the Context of Multi- and Intercultural Education. Int J Qual Methods. 2005;4(3):1–19.

51. Baraldi C. New forms of intercultural communication in a globalized world. Int Commun Gaz. 2006;68(1):53–69.

52. POVERTY AND SUSTAINABLE LIVELIHOODS IN THE SEASONALLY SUBMERGED HAOR AREA OF NETROKONA DISTRICT , BANGLADESH TALUKDER GOLAM RABBY THESIS SUBMITTED IN FULFILMENT OF THE REQUIREMENTS FOR THE DEGREE OF DOCTOR OF PHILOSOPHY FACULTY OF ECONOMICS AND ADMINISTRA. 2012;

53. Karim MA, Kabir MM, Siddiqui MA, Laskar MSI, Saha A, Naher S. Epidemiology of Imported Malaria in Netrokona District of Bangladesh 2013-2018: Analysis of Surveillance Data. Malar Res Treat. 2019;2019.

54. Sharmin S. Socio-Economic Situation and Land Rights of the Indigenous People in Bangladesh. OIDA International Journal of Sustainable Development. 2011;2(11):85–96.

55. Rahman SMB, Uddin MB, Hussain I. Anthropometric study on children of Garo and non-Garo families in Netrakona district of Bangladesh. Journal of the Bangladesh Agricultural University. 2011;9(2):267–72.

56. Rahman M. Paradoxes of participation of the women leaders in the union parishad in Bangladesh: A phenomenological analysis. Jurnalul Practicilor Comunitare Pozitive. 2019;19(2):58–73.

57. Merz S, Jaehn P, Pischon T, Fischer B, Wirkner K, Rach S. Investigating people ' s attitudes towards participating in longitudinal health research : an intersectionality - informed perspective. 2023;1–18.

58. Etikan I, Alkassim R, Abubakar S. Comparision of snowball sampling and sequential sampling technique. Biom Biostat Int J. 2016;3(1):55.

59. Atkinson R, Flint J. Accessing hidden and hard-to-reach populations: Snowball research strategies. Social research update. 2001;33(1):1–4.

60. Klar S, Leeper TJ. Identities and intersectionality: a case for Purposive sampling in Survey-Experimental research. Experimental methods in survey research: Techniques that combine random sampling with random assignment. 2019;419–33.

61. Roy P, Promila M. Quest for security, equality, equity and integration: Locus of Indigenous women in Bangladesh. Prof Chowdhury Mong Shanoo, editor Thailand: Asia Indigenous Peoples Pact (AIPP) and Kapaeeng Foundation. 2014;99–118.

62. Vinding D, Kampbel ER. Indigenous Women Workers With case studies from Bangladesh, Nepal and the Americas . ILO's International Labour Standards Department (PRO 169) . 2012. 1–58 p.

63. Beckett K, Hoffman B. Challenging medicine: Law, resistance, and the cultural politics of childbirth. Law Soc Rev. 2005;39(1):125–70.

64. Felton-Busch C, Larkins S. Remote dwelling Aboriginal Australian women and birthing: A critical review of literature. Women and Birth. 2019;32(1):6–15.

65. Bar-Zeev SJ, Barclay L, Farrington C, Kildea S. From hospital to home: the quality and safety of a postnatal discharge system used for remote dwelling Aboriginal mothers and infants in the top end of Australia. Midwifery. 2012;28(3):366–73.

66. Cidro J, Hayward A, Bach R, Sinclair S. Walking with our Most sacred: indigenous birth workers clearing the path for returning birthing to indigenous communities. Introduction to Determinants of First Nations, Inuit, and Métis Peoples' Health in Canada. 2022;225–36.

67. Yeung S. Conceptualizing cultural safety: Definitions and applications of safety in health care for Indigenous mothers in Canada. Journal for Social Thought. 2016;1.

68. Hayward A, Cidro J. Indigenous birth as ceremony and a human right. Health Hum Rights. 2021;23(1):213.

69. Akter S. Factors influencing health service utilization among mothers for under-five children: A cross-sectional study in Khulna district of Bangladesh. PLoS One. 2022;17(9):e0274449.

70. Moran A. White Australia, settler nationalism and Aboriginal assimilation. Australian Journal of Politics and History. 2005;51(2):168–93.

71. Butler R, Ben J. Centring settler colonialism in rural Australian multicultures: race, place and local identities. J Ethn Migr Stud. 2021;47(9):2179–97.

72. Dietz G, Mateos Cortés LS. Multiculturalism and Intercultural Education Facing the Anthropology of Education. A Companion to the Anthropology of Education. 2011;(July):495–516.

73. Celis K, Erzeel S, Mügge L, Damstra A. Quotas and intersectionality: Ethnicity and gender in candidate selection. International Political Science Review. 2014;35(1):41–54.

74. Spivak G. In Other Worlds: Essays in Cultural Politics. New York: Methuen. other worlds: essays in cultural politics. 1987;

75. Sauerbronn FF, Ayres RM, da Silva CM, Lourenço RL. Decolonial studies in accounting? Emerging contributions from Latin America. Critical Perspectives on Accounting. 2021;(xxxx):102281.

Dear Respected Reviewer(s),

Thank you very much for your kind and valuable comments and guidelines to revise the manuscript. The table below provides the list of your comments and how I addressed this.

**Abstract**<br>a. clarify if you used grounded theory or inductive thematic analysis. It looks to me that you used the latter.<br>b. You did not even mention the three levels of ethnocentrism in the results section of your abstract.<br>c. The conclusions in the abstract refer to two levels of ethnocentrism and mention a policy document that you did not mention before. Please, rewrite this section.<br>d. In academic English, the use of long sentences is highly discouraged. Long sentences reduce the readability of texts. Please, revise the English accordingly.	**a.** The revised one indicates the use of Janevic's conceptual framework to create analytical categories.[BACKGROUND SECTION, Lines in red color]<br>**b.** I revised the result section mentioning the three levels of ethnocentrism and their dimensions derived from the data.<br>**c.** I rewrite the conclusion section.<br>**d.** I tried my best to avoid using long sentences in the revised manuscript.
**Background**<br><br>a. You say your objective was to study disparities. However, you did not present any comparison with other groups. It looks like your study aimed to address the experience of Garo women in their access to maternal care. Please, revise.<br>b. Revise the paragraph with the definition of ethnocentrism. It is not clear.<br>c. The last paragraph is unnecessary. Please delete	a. Revised the objective of this manuscript to "exploing the how ethnocentrism influence Garo women's experience of accessing biomedical maternity care" [lines in red color, paraphaph 3 of the BACKGROUND section] [also revised it accordingly in the abstract section, line in red color of the background part]<br><br>b. I revised the meaning of ethnocentrism with proper citation, first at the begining of the BACKGROUND section [page 3, first line of paragraph 1, (highlighted in red color] and then in the last paragraph of the same section [page 5, lines in red color]

	c. I have deleted the last paragraph of the 'Background section' that presentd the structure of this manuscript.
**Methods**<br><br>a. What is the 'intersectionality-informed method' you used? Please, explain it briefly when you mention it.<br>b. Table 1 belongs to the results section. In the results, you should start by describing the participants. Also, Tables 1 and 2 could be integrated into one table.<br>c. better explain what are 'Lengura and Nazirpur Unions of Kalmakanda Upazila.' Remember, we have an international audience not familiar with Bangladesh.<br>d. You indicated Janevic's conceptual framework (methods) and then Anderson's framework (in the Discussion) were used to create analytical categories. However, you described an inductive analysis. Please, be more concrete and specific in describing what you did.	A. I explained the meaning of 'intersectionality informed method' in the METHOD section of the manuscript with proper citation. Also, mentioned why I used this approach. [lines in red color of the METHOD SECTION, [sub-heading: Sampling and recruitment. Page-7, lines in red color]<br><br>B. I put the table in the result section. As I removed data on KII respondents considering the word limit and focused solely on the women's perspective, I deleted table 2.<br><br>C. I provide some informaion about the Lengura Union and Nazirpur Union [lines in red color, page-5] and also mention where these two Unions are located. I also, include a Map for clear geographical locational indication. [page 6].<br><br>D. I mentioned about the use of Janevic's conceptual framework to create analytical categories, in the revised manuscript.
Results<br><br>a. You mention that Figure 1 has five domains 'for more transparent comprehension.' However, in Figure 1, it seems like you used three categories to summarise results.<br><br>I. Overall, you present interesting quotes, but they have a lot of repetition. Please, consider making some quotes shorter to facilitate readability.	a. In the revised manuscript, I revised the Figure 1 with some new dimensions. Also, revised the part second paragraph of the RESULT section that illustrates the Figure 1. [lines in red color]<br><br>I. I tried my best to revised the result section following the revised Figure 1 and remored all the repetitions.<br><br>II. I replaced the 'ethno-cultural' to culture.

II. What does Ethnocultural mean? Perhaps use cultural.	
III. What does Womanization mean? Please, consider not using this word if you could explain the idea with more accessible language.	III. In the revised manuscript I did not use the work 'womanization'.
IV. Page 11, last paragraph. You said 'Garo women's acceptance of the health practitioners' ethnic and occupational superiority may be the outcome of internalizing discriminatory attitudes encountered by the former group.' This is an assumption. Please delete.	IV. I deleted this paragraph.
V. Last sentence on page 13. Interestingly, while showing the ethnocentric aspects of maternal care, the author reproduces these views, for example, when describing mothers and mothers-in-law as ignorant people who cannot provide proper advice to pregnant women. Is this assumption supported by data? It is unacceptable that you describe Garo mothers as ignorant and discuss the cultural competency of their care simultaneously. Furthermore, this topic should be part of the health literacy part.	V. I deteled this paragraph.
VI. Clarify the first sentence of Health System Factors. Also, it is unclear if you are addressing health system-level issues here because the content is mostly about institutional care.	VI. I deleted this sentence.
VII. Please, avoid repetition. For example, the information in the last paragraph of page 15 was already mentioned on page 9 as part of a different category.	VII. I deleted the discussion on health system factor and focused on institutional, personally-mediated, and internalized ethnocentrism in the revised manuscript.
VIII. The last paragraph of the results belongs to the discussion. Please delete.	VIII. I deleted the last paragraph.

Discussion	
a. Why did you mention Anderson’s framework? This seems very unnecessary. Perhaps it is better to delete it.	a. I deleted it.
b. How did you jump from three categories of ethnocentrism to five types of discrimination? Please, avoid this or explain the methods and present the results.	b. In the revised manuscript I kept my focus on the three categories of ethnocentrism and the themes emerged from the data, avoiding external issues not conforming to the Figure 1.
**c. References 39-41 have nothing to do with the Upazila. They do not support your statement.**	c. I deleted this references in the revised DISCUSSION Section of the manuscript.
d. You should write a shorter discussion section which addresses the following topics (do not use these as subheadings): a statement of the principal findings; strengths and weaknesses of the study; strengths and weaknesses in relation to other studies, discussing important differences in results; the meaning of the study: possible explanations and implications for clinicians and policymakers; and unanswered questions and future research.	d. In the revised discussion, I tried my best to answer these questions.

Kind Regards.

The Author.
