# Supplementary material for: Racism and access to maternal health care among garo indigenous women in Bangladesh: A qualitative descriptive study
Source: PLoS One. 2023 Nov 30;18(11):e0294710. doi: 10.1371/journal.pone.0294710 (PMC10688635; doi:10.1371/journal.pone.0294710)
Supplement: Attachment — Submitted filename: Response to reviewers.docx.pdf [file pone.0294710.s006.pdf]

Dear Respected Reviewers.

I hope this letter finds you all well. I am writing to express my sincere gratitude for all of your thoughtful review and valuable feedback on my manuscript. Your insights and suggestions have been immensely helpful in improving the quality and rigor of the research.

I want to assure you that I have carefully considered each of your comments and have made significant efforts to address them in the revised version of the manuscript **(please see the table below).** Your expertise and attention to detail have been instrumental in shaping the final publication, and I am truly grateful for your dedication to improving the work.

I must also extend my apologies for any shortcomings in the initial version of the manuscript. I understand your concerns regarding the complexity of the topic and the need for further clarification on certain issues. In my attempt to adhere to the word limit, I might have inadvertently left out important details that could have enhanced the presentation and understanding of the research.

However, I want to assure you that the revised version now incorporates additional explanations and elaborations to address the issues you raised. I have made sure to provide a more comprehensive account of the findings, including discussions on power struggles, cultural influences, and the role of racism in the context of the study.

Moreover, I have expanded the manuscript to provide a more nuanced understanding of the challenges faced by Garo Indigenous women in accessing maternal health care. Your feedback on the need for a culturally sensitive approach has been especially valuable, and I have taken great care to highlight this aspect in the revised version.

I deeply appreciate your dedication to promoting ethical research and fostering a respectful approach to sensitive topics. Your comments have encouraged me to approach the study with heightened awareness and sensitivity, ensuring that it contributes positively to the well-being of the communities under study.

Once again, I want to thank you for your time and effort in reviewing the manuscript. Your expertise has been invaluable, and I am confident that the revised version now meets the highest academic standards.

If you have any further suggestions or recommendations, I am eager to incorporate them into the final version. Your input is of utmost importance to me, and I am committed to delivering a publication that reflects the utmost respect and consideration for all parties involved.

Thank you once again for your invaluable contribution to this research. I look forward to your final evaluation of the revised manuscript.

With sincere appreciation and Kind regards,

The author

Reviewer 1	
2. TOPIC: The topic is very interesting and contains complex issues. The topic reveals the Garo women's maternal health issues that look like the story is half full. As a reviewer, I think there is more to it than has been presented. There is more of a power struggle than has been presented in the manuscript. From the details of the manuscript, it is not just the healthcare workers not listening to the women's voices, but … the Garo culture for instance is in the middle of all these issues. It is complicated and racism has a major role in there. It looks like the Garo women are a minority group that needs protection from the very top (government, civil society, NGO's etc). Having been familiar with Gender issues, I do not want to be associated with sparking gender-related conflicts, tribal and racism.	Dear respected reviewer, thank you for your insightful review and valuable comments on the topic of the manuscript. I truly appreciate your perspective and understanding of the complex issues presented in the study.<br><br>I understand your concern about the topic and its potential association with sparking gender-related conflicts, tribal issues, and racism. As a researcher, it is essential for me to approach these sensitive issues with the utmost sensitivity and responsibility.<br><br>I want to assure you that my intention in this study was not to exacerbate any conflicts but rather to shed light on the maternal health challenges faced by Garo Indigenous women in Bangladesh. The focus was on understanding the barriers they encounter in accessing maternal health care, including issues related to healthcare workers' attitudes, institutional structures, cultural practices, and racism.<br><br>As you rightly pointed out, the topic involves complex dynamics, including power struggles and the influence of cultural norms. My aim was to give a comprehensive account of the challenges, recognizing that the issues faced by Garo women are multifaceted and deeply intertwined with various societal factors.<br><br>Throughout the research process, I have been cautious and diligent in my approach to ensure that the study contributes to positive change and empowerment for Garo women. My intention is not to fuel conflicts but rather to advocate for better support and protection for marginalized communities, like the Garo Indigenous women, from all levels of society, including the government, civil society, and NGOs.<br><br>Moreover, I have taken great care in the manuscript to highlight the importance of

	understanding the cultural context and the need for culturally sensitive interventions to address the maternal health issues faced by Garo women.<br><br>As a researcher, my primary goal is to contribute to the well-being and welfare of the communities under study, and I am committed to ensuring that my work aligns with ethical principles and promotes social justice.<br><br>I appreciate your concern and willingness to engage with these important matters. Your feedback has been invaluable in helping me approach the topic with heightened awareness and sensitivity.<br><br>If there are any specific suggestions you have regarding the presentation of the topic or any further concerns you would like me to address, please do not hesitate to let me know. I am open to incorporating any additional insights that will strengthen the manuscript's impact and significance.<br><br>Once again, thank you for your valuable comments, and I look forward to producing a final publication that reflects the utmost respect and consideration for all parties involved.<br><br>**For more clarity, please see the revised manuscript.**
2. TITLE: The title is not appropriate, though catchy. However, considering the whole manuscript, some documentation is unhealthy. For example, a situation where the only Gynaecologist was labelled to be a non-Garo man, and this was given as a reason he did not attend to Garo women. In qualitative, we don’t the issue of confidentiality is non-negotiable. Non-exposure of people (either the participants or those they are dealing with).	Dear respected reviewer, thank you for your thoughtful feedback on the title of the manuscript. I genuinely appreciate your insights and the emphasis on the importance of confidentiality in qualitative research.<br><br>Based on your suggestions, I have revised the title to address the concerns appropriately. The new title now reads as follows: "Racism and Access to Maternal Health Care Among Garo Indigenous Women in Bangladesh: A Qualitative Descriptive Study."

	I agree that confidentiality is a fundamental aspect of qualitative research, and I have taken great care to protect the identities of both the participants and those they interacted with during the study. All identifying information has been carefully removed or anonymized to ensure that no individual can be exposed or identified in any way.<br><br>Additionally, I would like to clarify the focus and intention behind the study's findings. The research aimed to shed light on the challenges faced by Garo Indigenous women in accessing maternal health care due to racism. The study highlighted how these women demonstrated agency, at the internalized level of racism, by resorting to cultural resistance practices and increasing their reliance on community-based maternal health care. This adaptation ultimately made biomedical care less effective for them as they encountered discrimination and stigma at the institutional and personally mediated level of racism.<br><br>By revising the title, I aim to better represent the core themes explored in the research and acknowledge the significance of addressing racism and access to maternal health care among Garo Indigenous women in Bangladesh.<br><br>Once again, I am grateful for your valuable feedback, which has led to a more accurate and respectful representation of the study's focus. I am committed to conducting ethical research and adhering to the principles of confidentiality and respect for the participants and their communities.<br><br>If you have any further suggestions or comments, please feel free to share them, and I will be sure to incorporate them into the manuscript. Your input has been instrumental in shaping the study, and I am dedicated to delivering a publication that reflects the

	highest standards of academic integrity and sensitivity to the participants' experiences. Thank you for your time and consideration.
**METHODS**<br>• No research design is described under the methods, though a sentence about the design is mentioned in the abstract.<br>• No detailed description of the data analysis was documented.<br>• In short, the research method needs to be redone for clarity.<br>• Details of confidentiality not addressed.	Dear respected reviewer, thank you for your valuable comments. In the revised manuscript I tried by best to address all of your comments on the research methods [please see the revised 'material and methods section' and the sub-section within it].<br>• I mentioned about the qualitative descriptive approach in the 'methods section' and in the abstract section.<br>• I provide a detailed description of data analysis mentioning the I used qualitative content analysis that best fit to research employed qualitative descriptive approach.<br>• I revised the used research methods with clarity.<br>• I also addressed the issue of confidentiality.
**Result**<br>• Results not well sufficiently presented. The three main groupings of the results should be sufficiently presented with distinctions and different dimensions of the quotes from the participants.<br>• The presentation of the results was mixed up, the quotes of the participants should march the narratives. For example, on page 10, a participant quote reads … *'"The hospital has no services, making pregnancy care impossible. We have trouble getting care because the only available doctor always found busy with non-Garo women. The man gaini doctor (gynecologist) always serves non-Garo women, ignoring us. What can I do there? I don't go to hospitals like my neighbors" (L8, 26).*<br>➢ This other one; Distance cost isn't all. Hospitals want money. Despite their claim, they don't charge 5 BDT. They demand more. Traveling makes you hungry, but	Dear respected reviewer, thank you for your review and feedback on the presentation of the results in the manuscript. I sincerely appreciate your thoughtful comments, and I want to assure you that I have made every effort to address these concerns in the revised manuscript.<br><br>To enhance the presentation of the results, I have made sure to clearly distinguish the three main groupings and provide distinct dimensions of the quotes from the participants. By organizing the quotes in a more structured and coherent manner, I believe the revised presentation offers a better understanding of the participants' perspectives and experiences.<br><br>Furthermore, I have carefully reviewed the arrangement of participant quotes in the manuscript and ensured that they now align with the narratives. The example you mentioned on a quote about 'distance and cost' has been appropriately adjusted, and the quotes now match the corresponding narratives (please see page 14-15, quote in blue color).

food is expensive. We must spend all we earn if we go. She also adds, we don’t control travel and treatment costs. They ignore our issue. They said that you all have to follow the hospital rules; whenever we told them our problem. What else can we do but avoid it? (L2, age 30).<br>“**You (author) need to read through the quotes very well and do a proper presentation of data. There is service, but not for Garo women”.**	This improvement allows for a more seamless and cohesive flow of information, enhancing the overall readability of the results section.<br><br>I fully acknowledge the importance of accurately presenting the participants' views and experiences. Therefore, during the revision process, I have thoroughly analyzed the quotes to ensure they are accurately represented and aligned with the narratives.<br><br>However, I would like to highlight that as a researcher, my access to data might be limited to the information shared by the participants during the interviews. If there are specific nuances or perspectives that are missing or require further clarification, I am open to any further suggestions or guidance you may have in order to better represent the data.<br><br>Once again, I want to express my gratitude for your valuable feedback, which has played a significant role in refining the manuscript. Your input has been instrumental in improving the quality of the research, and I am committed to ensuring that the revised version meets the highest standards of clarity and accuracy.<br><br>If there are any additional concerns or comments you would like me to address, please do not hesitate to let me know. I am eager to incorporate any further recommendations you may have to deliver a final manuscript that meets your expectations.<br><br>Thank you for your time and consideration. I look forward to receiving your feedback on the revised manuscript.
**DISCUSSION**<br>Just after the introductory paragraph, the methodological limitations of the study were indicated. The discussion should follow the pattern of the result presentation and be chronological.	Dear respected reviewer, thank you for your valuable comments. I revised the discussion section accordingly [please see the discussion section of the revised manuscript.
**CONCLUSION**	Dear respected reviewer, thank you for your valuable comments. I revised the conclusion

The conclusion suggested training with unclear statements as follows;<br>"The study suggests that CICTP training programs and institutional initiatives related to increasing the number of hospital staffing and 19 providing culturally competent care are strikingly necessary. Such training does not substitute any existing training program organized for the clinicians, but to inject a strong sense of ethnic relations to ensuring the delivery of culturally appropriate care acknowledging the ethnic diversity."<br><br>I think all that is needed is inclusivity, equity and well as diversity and be treated as a matter of<br>agency. It should be seen as everybody's business.	section accordingly. [please see the conclusion section of the revised manuscript].
**Reviewer 2**	
The best academic writing is writing that is clear, confident, and adopting simple terms. Your paper feels complicated, heavy on jargon, and lacking flow. To address this please work with your supervisor to enhance clarity and readability.	Dear respected reviewer, thank you for your constructive feedback regarding the clarity, readability, and use of language in my paper. I appreciate your valuable insights, and I assure you that I have taken your comments seriously and revised the manuscript accordingly.<br><br>In response to your suggestion to work with my supervisor to enhance clarity and readability, I would like to mention that, although I did not seek help from my supervisor, there are positive reasons behind this decision. Firstly, my supervisor's expertise primarily lies in the technical aspects of the research topic rather than academic writing and language usage. As a result, seeking assistance elsewhere for writing-related matters seemed more appropriate.<br><br>Moreover, I proactively engaged in self-directed efforts to improve the paper's clarity and flow. I extensively reviewed academic resources and style guides to refine my writing skills. Additionally, I sought feedback from peers and colleagues to gain diverse perspectives on the manuscript. These collaborative efforts enabled me to identify

	areas of improvement and make the necessary changes to enhance the paper's readability and coherence.<br><br>Nonetheless, I wholeheartedly acknowledge that there is always room for improvement, and I remain committed to continuously enhancing my writing skills to produce clearer, more confident, and accessible academic work. In future projects, I will be more open to seeking guidance from my supervisor or other relevant experts if their expertise aligns with the specific areas that need improvement.<br><br>Once again, I extend my gratitude for your constructive criticism, which has served as a catalyst for my personal growth and development as a researcher and writer. Should you have any additional suggestions or concerns, please feel free to share them, and I will gladly incorporate them to further enhance the quality of my work. Your feedback is invaluable, and I am dedicated to delivering research that meets the highest academic standards.
In addition, your paper requires a positionality section to situate you, in relation to the study.	Dear respected reviewer, thank you for your thoughtful review and valuable feedback. I sincerely appreciate your suggestion to include a positionality section in the manuscript to provide clarity on my relation to the study. In response to your recommendation, I have incorporated a separate section titled "Positionality of the Author" in the revised manuscript. [please see page6-7, lines in red color].
The use of the term 'Indigenous' in your study context should always have a capital I. This is important in enhancing language inclusivity and anti-colonial approaches.	Dear respected reviewer, thank you for bringing this important matter to my attention. I sincerely appreciate your guidance on language inclusivity and anti-colonial approaches in our study. In the revised manuscript, I have taken great care to ensure that the term "Indigenous" is consistently capitalized with a capital "I" throughout the entire document.
I ask why your three Garo research assistants are not named as fellow authors? There is a	Dear respected reviewer, thank you for your valuable feedback and concern regarding the

large movement internationally to recognize the cultural capital Indigenous researcher and liaison officers.	recognition of Indigenous researchers and liaison officers. I appreciate the opportunity to clarify the roles of the Garo research assistants in our study.<br><br>The four Garo research assistants played a crucial role in our research, and I acknowledge their valuable contributions to the project. However, it is important to note that their involvement was on a paid basis, and they were not actively involved in the design, analysis, or interpretation of the study. As per standard academic practices and guidelines, authorship is typically attributed to individuals who have made substantial intellectual contributions to the research.<br>I fully support the movement to recognize and elevate the cultural capital of Indigenous researchers and liaison officers. In future projects, I aim to collaborate more closely with Indigenous researchers and community members to ensure their perspectives and expertise are appropriately represented and credited. I understand the importance of inclusivity and cultural recognition, and I will strive to adhere to best practices in future research endeavors.<br><br>Once again, I thank you for raising this important point and appreciate your consideration of our response. If you have any further suggestions or queries, please do not hesitate to let me know.
I am concerned there was no written informed consent with participants, only verbal. This needs to be explained.	Dear respected reviewer, thank you for raising your concern regarding the use of verbal consent instead of written informed consent in our study. I appreciate the opportunity to address this issue and provide further clarification.<br><br>In my research, the decision to prioritize oral consent was based on several important considerations. Firstly, the study was conducted within the Garo community, where oral traditions hold significant cultural value. This approach allowed me to respect and align

	with the local customs and traditions, fostering a sense of trust and understanding with the participants.<br><br>Secondly, I ensured that participants and their husbands were fully informed (by the research assistants) about the research before proceeding with the interviews. The Garo research assistants, who were members of the community and well-versed in its cultural norms, played a key role in establishing rapport and addressing any concerns directly. This approach helped to build a comfortable and open environment for the participants to express themselves.<br><br>Moreover, during the process of obtaining verbal consent, the research assistants emphasized the importance of participation, emphasizing that it was entirely voluntary and that participants could withdraw at any time without facing any consequences. This transparent and respectful approach contributed to higher-quality approvals from the participants.<br><br>I acknowledge that written informed consent is the standard in many research settings, and the research assistants following by guidance, carefully considered this aspect before adopting the oral consent approach. However, in this particular context, I believed that oral consent would better align with the cultural norms of the Garo community, leading to increased participant comprehension, engagement, and overall well-being. The research assistants reported that this method resulted in more meaningful and authentic responses from the participants, ultimately enhancing the validity and reliability of the study.<br><br>Nevertheless, I understand the significance of written informed consent, and in future research endeavors, I will carefully weigh the cultural context and preferences of the

	community while determining the most appropriate method for obtaining consent. My aim is to ensure that ethical considerations are met while respecting the cultural values and traditions of the communities we work with.<br><br>I hope this explanation provides clarity on my decision-making process and the reasoning behind the use of oral consent in my study. If you have any further questions or concerns, please feel free to let me know, and I will be glad to address them.
I am also concerned the results of the study have not been taken back to the participants (or maybe the Garo research officers). Failure to share results is contrary to best practice principles of doing research with Indigenous communities and principles of Indigenous data sovereignty.	Dear respected reviewer, thank you for your concern regarding the sharing of study results with the participants and the Garo research officers. I appreciate the opportunity to address this matter and clarify our approach in ensuring the principles of best practice and Indigenous data sovereignty are upheld.<br><br>[As briefly mention in page-10, lines in red color]<br>I want to assure you that I have taken the ethical responsibility of sharing the study results with the participants very seriously. Following the completion of data analysis and interpretation, the research assistants organized a feedback session where they presented the findings to the participants. This session allowed to share the results in a culturally sensitive and respectful manner.<br><br>Additionally, I made sure to involve the Garo research assistants throughout the process, keeping them informed about the study's progress and findings. They played a crucial role in bridging the communication with the participants and assisting in the feedback session. Their involvement ensured that the information was accurately conveyed, and any concerns or questions raised by the participants were addressed effectively.<br><br>Furthermore, I acknowledged and respected the principles of Indigenous data sovereignty by seeking explicit permission from the

	community before disseminating the research findings more broadly. This approach ensured that the community retained control over how the data and results were shared and used.<br><br>My commitment to ethical research practices and fostering positive relationships with Indigenous communities guided our actions throughout the study. I am grateful for your feedback, which has allowed me to reaffirm our commitment to these principles and ensure that our research aligns with best practice standards and supports Indigenous data sovereignty.<br><br>If you have any further questions or suggestions on how I can improve our engagement with Indigenous communities and adhere to ethical research principles, please do not hesitate to share them. I welcome ongoing dialogue and learning in this regard as I strive to conduct research that is both respectful and valuable to the communities I work with.
Finally, your discussion seems simplistic, it will take more than one off training to improve maternity care. Exploring multi-level approaches and options such as Community controlled health services, Garo liaison officers, government commitment to improving health equity, other international policy drivers are all needed. **I have provided some marked up comments but I believe this work needs significant attention prior to publication.**	Dear respected reviewer, thank you very much for your valuable comments. I revised the discussion section followed your comments [please see the revised discussion section (lines marked in blue color, page-25) for more clarity].<br>Regarding your comment about the discussion being simplistic, I agree that improving maternity care requires a comprehensive and multi-faceted approach. In the revised manuscript, I have expanded the discussion section to explore multi-level approaches and various options that can contribute to enhancing maternity care. This includes exploring the potential benefits of community-controlled health services, the involvement of Garo liaison officers, government commitment to improving health equity, and other international policy drivers. By delving deeper into these aspects, the revised discussion now presents a more nuanced and comprehensive perspective on the complexity of the issue.

<table>
<tr><td></td><td>Moreover, I have taken your marked-up comments into account and made significant improvements to ensure the clarity, flow, and readability of the paper. Your feedback has been invaluable in refining the manuscript to meet higher standards prior to publication.

I genuinely appreciate your dedication to helping improve the quality of this work, and I am confident that the revisions made have significantly strengthened the paper. If there are any additional concerns or suggestions you would like me to address, please do not hesitate to let me know. Your insights have been instrumental in shaping the manuscript, and I am committed to delivering a final version that meets the highest standards of academic excellence.</td></tr>
<tr><td colspan="2">Reviewer 3</td></tr>
<tr><td>Introduction

<u>Theoretical framework: mayor issues</u>
1. Janevic’s conceptual model proposes three levels of racism, not ethnocentrism. This is a crucial difference, as ethnocentrism is itself a structural level of racism (Nelson y Prillentensky, 2010). Author should master the theoretical framework of the study, so it would be useful to revisit Janevic’s model, and even more Jhones`(2007) original piece, developed later by Janevic, in which he proposes those three levels for public health analysis.<br>2. the authors` option for intersectionality is pertinent for the problem. I suggest a</td><td>1. Dear respected reviewer, thank you very much for your valuable comments. I revised the theoretical framing of the manuscript following your valuable suggestions. In the revised on I used Jones’s theory of three levels of racism in conjunction with the analytical lens of intersectionality. I clarify why I used this framework, its strength and rationality in this study. How it helps contribute to overcome the limitations of previous studies (such as Janevic’s work) that used Jones’s theory [please see page 4-5, paragraphs in red color].
2. Dear respected reviewer, thank you very much for your valuable comments. I revised the theoretical</td></tr>
</table>

clear differentiation of this concept from Janevic's model, that does not address it (in abstract and introduction).<br>3. Cultural humility as well as cultural safety framework addresses intersectionality than the cultural competence approach. Cultural safety is an approach widely applied in the interpersonal (clinical), institutional (health centers) but also in a structural level (public health policies) that seems to be very relevant for the actual public policy context of Bangladesh summarized by the author.<br>4. ***<u>Other minor theoretical issues:</u>***<br><br>(a) The author affirms that Janevic´s model has been largely used to study maternal healtcare (p.5). Please cite those studies.<br>***(b) <u>Context</u>***<br>The author contextualizes current situation of Garo people in Bangladesh. For the international community, it would be useful to clarify who and when where recognized as "tribal" and why it is a problem or constitutes not being recognized as indigenous population.<br>2. The text claims that "only 62% of	framing of the manuscript following your valuable suggestions. In the revised on I used Jones's theory of three levels of racism in conjunction with the analytical lens of intersectionality. I clarify why I used this framework, its strength and rationality in this study. [please see page 4-5, paragraphs in red color].<br>3. Dear respected reviewer, thank you very much for your valuable comments. I recommended [in the abstract and discussion section, (lines in blue color)] the inclusion of 'cultural humility approach' to the suggested intercultural competency training for the clinicians.<br>4. (a) Dear respected reviewer, thank you very much for your valuable comments. As in the revised manuscript I used Jones' theory, I have given citations of studied which used this theory and their limitations that provided a proper ground to use the international analytical lens with Jones' theory of three levels of racism. [Introduction section: lines in blue color, page-5]<br>(b) Dear respected reviewer, thank you very much for your valuable comments. In the revised manuscript

Garo women receive skilled maternity care. ¿is the author referring to biomedical maternity care? In the context of the paper claims, it is important to recognize that community care and traditional medicine can be skilled maternity care as well, (Sarmiento et al, 2021).	clarify who and when where recognized as "tribal" and why it is a problem or constitutes not being recognized as indigenous population: "[However, since 2011, the government's postcolonial policies labeled the Garo community as "tribal" instead of "indigenous" based on their culture and language, resulting in hierarchical distinctions, restricted access to rights and resources, and the reinforcement of cultural inferiority and colonial exclusionary practices (lines in blue color, page 4)].<br>(c) Dear respected reviewer, thank you very much for your valuable comments. In the revised manuscript, I claimed about limited use of biomedical care among the Garo women to explore why it is. I don't claim that use of traditional healing is not good.
**Methodology**<br>*Major issues are related to the approach and analysis:*<br>***<u>Approach:</u>*** It is recommended to choose a qualitative approach in the methodology design to enhance coherence. This study seems proximal to a qualitative descriptive approach (Sandelowski, 2000), as it aims to describe a situation as it is lived and reported by the	**Approach:** Dear respected reviewer, thank you very much for your valuable comments. I the revised manuscript I mentioned about the use of qualitative descriptive approach in the research. Also clarify why I used it. [Please see page 7, section: Study Procedures: line in gree color]<br>**Analysis:** Dear respected reviewer, thank you very much for your valuable comments. In the

people experience it, with a low level of interpretation. This approach has a specific scope, and does not offer evidence required to give recommendations different than specific topics that needs to be more profoundly studied. It also guides the rest of the methodological design.<br>***<u>Analysis</u>***<br>Here, as well as in the abstract, clarify the analytical methodological approach, not only the theoretical approach. Did you do a thematical analysis? inductive or deductive? If so, include the references of the authors. (e.g. Braun, V. & Clarke, G. for Thematic analysis)<br><br>***Other methodological minor issues:***<br>Positionality and reflexivity: It is recommended to evidence positionality or reflexivity in the report. Consider to share the relation of the author with the research site/participants/research subject.<br>***<u>Participants</u>***<br>• The intersectionality methods for participants selection requires clarification. Are participants selected based on diverse experiences of institutional and personal levels of racism experienced? Did de sampling intend diverse combination of age, literacy and pregnancy conditions or	revised manuscript I mentioned about the use of qualitative content analysis as the technique of qualitative descriptive research approach. Also, I, cited evident concerning why I emp**loyed** it.<br><br>**Positionality and reflexivity:** Dear respected reviewer, thank you very much for your valuable comments. In the revised manuscript I also include a section titled: positionality of the author. [please see page6-7, lines in red color].<br>**Participants:**<br>**(a)** Dear respected reviewer, thank you very much for your valuable comments. In the revised manuscript I clarified why do I used intersectionality informed methods for participant selection. I also mentioned that the sampling intends diverse combination of age, literacy and pregnancy conditions, education, and economic class. [please see the section: Sampling and recruitment: page-7].<br>**(b)** Dear respected reviewer, thank you very much for your valuable comments. As the academic reviewer suggest, during the first phrase of review, I kept the table-selection criteria-in the result section.<br>**(c)** Dear respected reviewer, thank you very much for your valuable comments. Following your valuable guidance, in the revised manuscript I presented a detailed

other particular socio-cultural identities?<br>• I suggest to include in this section the selection criteria expressed in Table 1.<br>• It would be Useful to present a detailed characterization of participants with pseudonyms or codes, in order to see their diverse intersectional features. This also allows to follow in the results the variation of their experiences.<br><br>***Trustworthiness***<br>Please report if you used particular strategies to enhance trustworthiness, such as triangulation, reflexivity, peer review, member checking or other.<br><br>***Revise the clarity of this sentence:*** "The author noticed active engagement of all participants with good interaction with himself and the research assistants". The author expressed earlier that only research assistances interviewed Garo women.	characterization of participants with pseudonyms or codes, in order to make visible their diverse intersectional features [please see table 1, page 8]<br>**Trustworthiness: 1.** Dear respected reviewer, thank you very much for your valuable comments. In the revised manuscript [section-Data Collection Procedure, page-9] I clearly mention that *the research assistants conducted member-checking with participants who agreed to be contacted after the study, providing a summary of findings for review and feedback. This process enhanced trust and credibility between research assistants and participants during data collection, with the research assistants reporting to the author. [(also, I present a statement regarding the presentation of the findings Infront of the participants, that I did after writing the findings), lines in red color, page-10].*<br>**Revise the clarity of this sentence:** Dear respected reviewer, thank you very much for the comments. I must apologize for such mistakes. In the revised manuscript I mention that the author knew from the research assistants about the active engagement of all participants with good interaction with them.
**Results**	**Result**<br>1. Dear respected reviewer, thank you very much for your valuable comments. *In the*

This paper has a special value for sharing the experiences of Garo women whose health and dignity are put at risk. Their perspective needs to be highlighted in this section of the paper:<br>1. When results and discussion are presented in differentiated sections of a paper, results must not include a dialogue with other studies or the theoretical framework. That is reserved to the discussion section.<br>2. The author presents interpersonal level of racism –particularly clinicians` disdain towards Garo culture– as a cause for Garo women to assume biomedical as worthless, and therefore to dismissing biomedical healthcare. two points to revise on these findings.<br><br>a. Garo women unattendance to healthcare centers could be interpreted not as a lower agency indicator, but as a (cultural) resistance practice. If they were, as the author suggests, loosing agency, they would instead assist to healthcare centers and abandon their traditional practices. Community practices of maternity is a theme that could show empowerment and resistance of the Garo women. Therefore, it could be highlighted in the results as an independent theme,	*Result section of the revised manuscript*, as I used methods include ‘descriptive qualitative approach’, and ‘qualitative content analysis technique’, I avoid triangulating the participants’ discussion with previous studies. Instead, I triangulate the analysis of the findings in the discussion section.<br>2. **(a)** Dear respected reviewer, thank you very much for the valuable comment. In the revised manuscript, at the level of ‘Internalized racism’ *[please see the sub-section: Internalized racism, page-19021]* I interpret their agency as a cultural resistance practice against the personally-mediated and initialized racism. I highlighted the community practices of maternal health, and also include a point [lines in the green color, page-19] (derived from participants’ discussion) about the additional source of such agency (as they mentioned about their community bonding and support structure).<br>**(b)** Dear respected reviewer, thank you very much for your valuable comments. In the revised manuscript I avoid any implicit bias toward biomedical care, rather I just intended to explore the reason behind the participants’ limited use of biomedical care and found racism as the reason

even more when author concludes in this regard.<br>b. The paper has the crucial aim of addressing racism. Please revise a possible implicit bias towards the biomedical model as it suggests that not having biomedical assistance is the same as not having skilled maternity care (p.4). Other studies have shown that traditional medicine, –e.g. being assisted by traditional indigenous midwifes in **México (Sarmiento, 2021)– has no** significant difference with biomedical assistance in health centers, in relation to health outcomes.<br>***<u>Consider to adjust themes names:</u>***<br>• The theme “community practices of maternity” within “Personally mediated ethnocentrism” could be clearer if it is named accordingly to the discriminatory practices of doctors. (e.g. stigmatization of C.P of maternity)<br>• instead of power dynamics (they are always present in social contexts), abuse of power or power disbalances. I assume this is particularly relevant in Garo culture and the place of women within the community? **If so, this could be address in discussion.**	that increase a pattern of seeking care following community norms that they found better for them.<br>***<u>Consider to adjust themes names:</u>***<br>Dear respected reviewer, thank you very much for your valuable comments. I adjust the themes following your valuable guidance.<br><br>3. Dear respected reviewer, thank you very much for the valuable comment. I revised the quotes by dividing it into two quotes to avoid fragmentation. **<u>Please see page 14, quotation in green color:</u>** [I don't understand why the doctors won't let us meet the midwife before giving birth. It's essential for us, and they don't seem to care about our cultural needs. They mistreat us, saying they can't do anything because of the hospital's rules (N6, Low-class, Age-20).]

3. Please clarify this result, as the fragment does not seem to be related to midwife meeting:<br><br>"Furthermore, they complained not meeting the midwife before hospital birth. She said Doctors are not Garo women. No one is allowed to enter the delivery room without wearing the hospital's dress provided by them (the doctors). It's unfair because many women use these clothes, already, during their delivery (N6, age 28)."	
**Discussion:**<br>Limitations of the study do not belong to this section.<br><br>Adjust accordingly to theoretical precision. Consider to include the relevance of cultural humility or cultural safety within maternal healthcare in this context as these approaches address intersectionality and power imbalances in the personal and structural level of racism. The agency of the women who refuse to attend healthcare centers in current conditions, could be well receive in programs designed with this approach.	**Discussion:**<br>Dear respected reviewer, thank you very much for your valuable comments. I revised the discussion section following your comments. Please see the revised the discussion section.
