# Supplementary material for: Racism and access to maternal health care among garo indigenous women in Bangladesh: A qualitative descriptive study
Source: PLoS One. 2023 Nov 30;18(11):e0294710. doi: 10.1371/journal.pone.0294710 (PMC10688635; doi:10.1371/journal.pone.0294710)
Supplement: Attachment — Submitted filename: RESPONSE TO THE REVIEWERS COMMENTS.pdf [file pone.0294710.s008.pdf]

Dear esteemed reviewer,

I would like to express my sincere gratitude for your invaluable feedback on my manuscript. Your insights and recommendations have been immensely helpful in refining the quality and clarity of the research.

I am pleased to inform you that I have diligently addressed each of your suggestions, and the changes have been incorporated into the revised manuscript. To provide a clear overview of the modifications, I have compiled them in the table below:

Response to the Reviewers Recommendations

**Acknowledgement**<br>I strongly suggest you include at least in this section the acknowledgment of the Garo research assistants, whom not only participated in data collection, but also in the analysis and member checking strategy. Do the same with the teacher.	Dear respected reviewer, thank you for your thoughtful suggestion. In response to your recommendation, I have included acknowledgments for both the Garo research assistants and the teacher in the appropriate section of the manuscript. Their significant contributions to data collection, analysis, and member checking have been duly noted. Your feedback is greatly appreciated.
**Study procedure**<br><br>• Review this phrase: "This study utilized a qualitative descriptive approach, commonly employed in semi-structured interviews". Do you mean "qualitative descriptive approach, recollecting data with semi-structured interviews"?<br>• Author should expand the description of the method`s scope and utility, explained by Sandelowski: it aims to explore and describe a situation, generally understudied, as it is lived and reported by the people whom experience it. Remember that this approach has a specific scope. It is exploratory and it generally does not have evidence deep enough to support recommendations (as policies). Researchers discuss and conclude based on the data, However, they may point central phenomena to take into consideration in further studies or policy making.<br>• **Sampling:** review the sentence " The participants were categorized into these three groups based on… " ¿which groups?.<br>• **Sampling. Table 1.**<br>This table in qualitative descriptive studies is important as it shows the variation of the sampling, highly valued	1. Dear respected reviewer, thank you very much for your valuable comment. I revised the sentences as: This research was guided by the qualitative description approach. Qualitative description is widely used methodological approach, especially within practice disciplines.<br>2. Dear respected reviewer, I addressed this comment following your guidelines. [please see the 'procedures' section of the revised manuscript.<br>3. Dear respected reviewer, thank you for your feedback. The sentence you mentioned has been revised as follows: "The author categorized their household income into three classes: high, middle, and low, based on their monthly household income in comparison to the national average of monthly household income, which was 15,988 BDT as per Bangladesh Bureau of Statistics (BBS) data."<br>I believe this revision provides a clearer explanation of how the participants were categorized based on their income levels. [please see the result section, page 11]<br>4. Dear respected reviewer, thank you very much for your valuable comment. I have addressed your valuable comments by making the following revisions:

in this kind of research. Variation in the participant´s group assures an expanded exploration of an understudied phenomena. Participant table could be helpful to indicate possible areas or populations that require further research. For this, we find this table in the results section.<br>• Include in this section (sampling) only table 2, adding all the inclusion criteria.<br>• As author have made significant improvements in results and discussions highlighting intersectionality relevance, table 1 is more useful in the first part of the results. This table must be clarified as follows.<br><br>-Please revise precise data of every participant, as in the current table we have mixed information.<br>-Briefly explain how Household economic status is determined in your context.<br>-Clarify what “class five” means in your context ¿basic education or high school?<br>-Briefly explain what does “Self-educated” mean in Garo women context.<br>-Pregnancy diversity can be better understood if it is shared how many children have been born and if they are or not currently pregnant.	• I have relocated Table 1 to the first part of the results section, as suggested by the reviewer. This adjustment helps emphasize the variation in participant groups and the potential areas for further research, aligning with the goals of qualitative descriptive studies.<br>• In response to your recommendation, I have included Table 2 in the sampling section, incorporating all the inclusion criteria. This ensures that the sampling section comprehensively presents participant characteristics.<br><br>- I have revised and provided precise data for each participant, ensuring that the information in the table is clear and well-organized.<br><br>-I have included a brief explanation of how household economic status is determined in the context of the study.<br><br>-I clarified that "class five" in the context refers to primary level of education.<br><br>-I have replaced the term "Self-educated" to illiterate as it will be easier to understand for the reader.<br><br>-I have included information about the number of children each participant has and their pregnancy status to enhance the understanding of pregnancy diversity.
**Analysis**<br>1. In this new version author reports doing content analysis. Even though this approach is highly used in qualitative descriptive analysis, results are presented in themes. There is a common confusion between content and thematic analysis in qualitative descriptive analysis and needs to be avoided. Vaismoradi, Turunen and Bonda clarify how to address this risk in their paper. Please adjust analysis, briefly highlining the process that makes it content analysis or thematic analysis, to	**Analysis**<br>1. Dear respected reviewer, I appreciate your valuable feedback, and I have made the necessary revisions to the analysis section to ensure methodological coherence and transparency. In the revised version, I have explicitly mentioned that I used 'thematic analysis' as the method for analyzing the data. This adjustment helps clarify the approach taken in the study, aligning it with the methodological choice and addressing any potential confusion between content and thematic analysis.

address methodological coherence and transparency in the report.<br>2. Also, clarify if it is a deductive or inductive process. Apparently, the author did a deductive analysis, based on Jones' racism framework, combined with an inductive analysis, as there seem to have emergent categories (not addressed in Jones´ racism theory): Agency; Cultural resistance practice; Community practices of maternal healthcare (traditional medicine practices of maternal health?).	Thank you for highlighting this important point, and I hope the clarification improves the clarity of the report.<br>2. Dear respected reviewer,<br>Thank you for your insightful comments. I have addressed the issue of deductive and inductive analysis in the revised 'Analysis' section of the manuscript. I explicitly mention that I employed both inductive and deductive approaches in a complementary manner.<br>I clarified the rationale behind this choice, explaining that while I initially used a deductive approach based on Jones' racism framework, I also allowed for the emergence of additional categories that were not addressed in Jones' theory. This combination of deductive and inductive analysis enriches the understanding of the data and ensures that both the pre-defined and emergent themes are thoroughly explored.<br>I hope these clarifications provide a more comprehensive view of the analysis process in the study. Thank you for your valuable feedback, which has contributed to improving the rigor and transparency of the research.
**Trustworthiness:**<br>consider to include this section in the procedure as you develop multiple strategies to enhance rigor in your context: triangulation between assistants and researcher during analysis; member-checking of results with participants. Also include here positionality of author and the strategies used to enhance cultural sensitivity with the support of Garo women assistants and the community teacher.	Dear respected reviewer,<br>Thank you for your suggestion regarding the inclusion of a 'Trustworthiness' section in the procedure. I appreciate your thoughtful input. I want to inform you that I have indeed included a separate section titled 'Trustworthiness' immediately after the 'Analysis' section in the revised manuscript. In this section, I have addressed multiple strategies employed to enhance the rigor of the study.<br>I also merged the section on 'Author's Positionality' with the section titled 'Research Site' to provide a more comprehensive understanding of the context and the strategies used to enhance cultural sensitivity with the support of Garo women assistants and the community teacher.<br><br>I believe these revisions have strengthened the manuscript, and I hope they align with your expectations regarding the presentation of trustworthiness measures. Your feedback has been instrumental in refining the study, and I am grateful for your guidance.

**Results**<br><br>Author improved significantly the results report. Fragments included about traditional medicine practices, and about women agency and trust in their culture are valuable. However, because of the way results are presented, there is a major theoretical incoherence that could have a simple solution:<br>1. How does agency, Cultural resistance practice. and Community practices of maternal healthcare constitute internalized racism? It is exactly the opposite of internalized racism. If women had internalized racism (as in the Mexican study you cite on discussion), they would prefer to go the hospitals instead of their midwifes, they would not be proud of their culture, they would even believe they deserve bad treatment.<br>2. This emergent theme is crucial to understand and support Garo Culture and Garo women strategies to face racism at bio-medical centers. Protecting Garo Culture and women agency is as necessary as transforming the biomedical healthcare institution, from the theoretical framework reported. I strongly suggest to create an additional theme grouping these sub-themes, related to cultural strength or resistance towards racism.<br>3. Even though the author clearly has the intention to support Garo Women and Garo culture from racism, the model continues to suggest that more dependency on the cultural practices of maternal healthcare is a negative effect of institutional racism and internalized racism. therefore, it suggests cultural strength cause poor access to biomedical maternity care. In this regard consider that:<br>-there is no sufficient data evidence that describe how more dependency on traditional medicine leads to poor access to biomedical maternity care.<br>-Presenting a model suggesting these relations may contribute to enhance institutional racism, if the reader misunderstand that traditional medicine and cultural practices (and not racism directly) are the reason Garo women do not seek bio-medical maternal health care.	Dear respected reviewer,<br><br>I sincerely appreciate your careful examination of the results and your thoughtful suggestions. I have taken your feedback seriously and made the following revisions to address the issues you raised:<br><br>• **Clarification of Internalized Racism:** I understand the concern you raised regarding the terminology and its alignment with the emergent themes. To address this, I have revised the discussion of the emergent theme related to cultural strength and resistance towards racism to ensure that it is consistent with the model and does not suggest that cultural strength causes poor access to biomedical maternity care. Instead, I have emphasized that this cultural strength represents a resistance strategy against institutional and personally-mediated racism. This revision aligns with the Garo women's experiences and perspectives as reported in the study.<br>• **Adjustment of the Model:** I have revised the model to accurately reflect the relations reported by Garo women. The revised model now highlights that poor access to biomedical maternal healthcare is rooted in institutional and personally-mediated racism. It also acknowledges the emergent broad theme “women Agency” as a resistance strategy against these forms of racism. Internalized racism has been removed from the model, as there was no sufficient data evidence to support its inclusion.<br>4. Dear respected reviewer,<br>Thank you for your valuable feedback regarding the language used in describing the hospital's role in the study. I would like to clarify that the issue of exploitation was commonly shared among all the Garo women participants in the study. Their collective experiences highlighted a systemic concern that needed to be addressed.<br>However, I also understand your suggestion to strike a balanced tone when discussing the hospital's role. While the

-The solution would be to adjust the model, identifying the relations reported by Garo women only: a) Poor access to biomedical maternal healthcare is rooted in institutional and interpersonal; b) there does not seem to be reports of internalized racism. If so, remove it from the Figure. c). the emergent theme (related to strong Cultural traditional-medicine practices / women agency) may work as a resistance towards both types of racism.<br>4. Author may want to attenuate the affirmation of the hospital exploiting Garo women, taking into account a) ¿is it an experience of all the women in the study? (if so, author should report the abuse, if not, author could report the experience of some of the women with some of the staff) b) does the author want the hospital to be open to this study suggestions, and not defensive towards the claims?<br>• P. 16: Adjust this sentence: "This illustrates a link to personally-mediated ethnocentrism, as presented in the following sub-section". (Racism?)	experiences were consistent among participants, it is essential to ensure that the language used remains constructive and encourages collaboration and dialogue with healthcare institutions. I have adjusted the text to reflect this approach, acknowledging the shared experiences of exploitation while maintaining a respectful and open stance toward potential changes within the healthcare system.<br><br>These revisions aim to provide a more accurate representation of the study findings and the experiences of Garo women while fostering a constructive dialogue with healthcare institutions.<br><br>I appreciate your feedback, which has contributed to enhancing the clarity and sensitivity of the study's language and approach.<br>• Dear respected reviewer, I have revised the sentence as per your suggestion, replacing "ethnocentrism" with "Racism" in the revised manuscript. Thank you for pointing out this correction.
**Discussion and conclusion:**<br>1. Results do not suggest how to solve the problem (implementing intercultural training programs) and it is not the scope of this kind of study. How ever, author can attenuate the affirmation, focusing on concluding facts that the study shows based on the evidence of the reports of Garo women: there is strong racism at different levels, and it strongly compromises biomedical healthcare access. Garo women count on a strong traditional medicine system to attend maternal healthcare. Garo women agency derives in their decision to avoid hospitals were they and their culture is mis-respected. Therefore, to increase biomedical healthcare access for Garo women racism needs to be prevent in the institutional and relational level. Author then can suggests the models to explore for future policy-making.<br>2. In discussion and abstract the author refers to Inclusive approaches like	1. Dear respected reviewer,<br>I want to express my sincere gratitude for your insightful comments and recommendations. Your feedback has been instrumental in refining the manuscript, and I truly appreciate your time and expertise. I have incorporated your suggestions into the discussion section to provide a more nuanced and evidence-based conclusion.<br>In light of your input, I have adjusted the discussion as follows:<br>Based on the evidence from the reports of Garo women, it is evident that racism exists at different levels and significantly compromises their access to biomedical healthcare. Garo women have a strong reliance on traditional medicine for maternal healthcare. Their agency is rooted in their decision to avoid hospitals where they and their culture are not respected. Therefore, to enhance biomedical healthcare access for Garo women, it is imperative to address racism

'Community Controlled Health Services' and 'Garo liaison officers'. These programs have not been previously presented, so the relation with result is unclear. Consider to present as examples, or expand it in the introduction.	at both the institutional and relational levels. "then I suggest for implementing intercultural competency training program (CICTP) for the clinicians".<br><br>2. Dear respected reviewer,<br>Thank you for your comment regarding the mention of 'Community Controlled Health Services' and 'Garo liaison officers' in the discussion and abstract sections of the manuscript. I appreciate your feedback and have made the necessary revisions based on your suggestion.<br>I have removed the policy recommendations related to these inclusive approaches from the discussion section as they were not adequately introduced or connected to the study's findings. Instead, I have focused on highlighting the broader implications of the study's results and the need for addressing racism at different levels within healthcare institutions, as per your earlier recommendations. This change was made to ensure clarity and relevance in the discussion section.<br>Once again, I want to express my gratitude for your valuable input, which has contributed to improving the coherence and effectiveness of the manuscript.
**Other minor revisions:**<br>Here are some fragments that need writing revisions, but a final review of the writing is required.<br>• Revise last phrase on p.4 "It is argues that a consideration of the women's varied social contexts and diverse identities is essential in comprehending their maternity care experiences (44)."<br>• Revise redaction of Jones's 'three levels of racism' new paragraph in introduction.<br>• P. 5. Revise this redaction: "Given such strength, Jones's theory, evident in studies like Janevic's on Romani women(43) and others on Indigenous women (3,39,64), highlights racism's impact on maternal healthcare…" and "The Upazila hospital has medical staff, including non-Garo male	• These studies often lack a context-specific understanding of intersectionality and overlook the perspectives of Indigenous women who face compounded prejudice and implicit bias due to various aspects of their identity such as gender and economic class. To comprehensively grasp Indigenous women's maternity care experiences, it is imperative to consider their diverse identities within distinct social contexts.<br>• The study employs Jones's 'three levels of racism' framework (22) along with Crenshaw's intersectional lens (19) to frame and analyze the themes emerged from participants' discussion. Jones's model categorizes racism into three levels: institutional, personally-mediated, and internalized (22). This breakdown helps

gynecologist, with most of the pregnant patients seeking care there are not Garo women”.<br>• Positionality: Consider including this section with no title, but merged with the setting or in the suggested section of trustworthiness. In this phrase “The author has a distinct positionality concerning the research site and the participants” what do you mean by distinct positionality?<br>• p.9 This process enhanced trust and credibility between research assistants and participants during data collection, with the research assistants reporting to the author.<br>• P. 14. “As another participant shared, the issue of culturally inappropriate care extends to the midwifery meeting”. Do you mean “to the participation of the midwife ”?<br>• P14. This explanation may be removed as it is contains in previous paragraph: “This participant expresses concern about the absence of Garo female doctors, as seeking care from a non-Garo male doctor goes against their cultural norms of not sharing personal health problems with male doctors”.<br>• P.16. Differentiate researchers’ voice from participants: “Distance cost isn’t all. Hospitals want money. Despite their claim, they don’t charge 5 Taka (Bangladeshi currency). They demand more. Traveling makes you hungry, but food is expensive. We must spend all we earn if we go. She also adds, we don't control travel and treatment costs. They ignore our issue. They said ‘you have to follow the hospital rules’ whenever we told them our problem. What else can we do but avoid it? (L2, Middle-class, Age-30).<br>• P.21. Consider removing this part of the fragment that does not add much to what is being shown: “I don’t know. Probably would have sent me to a free hospital maybe” (L7, Low-class, Age-27).	providing a deeper understanding of the complex impact of racism on maternal healthcare access. Unlike models that primarily focus on the social construction of race or ethnicity and the structural factors perpetuating racial inequalities (64–66), Jones’s comprehensive approach offers a holistic understanding. It suggests that institutional and personally-mediated racism contribute to the internalization of racism within the context of maternal healthcare access (22,28). Given its strong theoretical foundation, Jones’s model is particularly relevant for our study.<br>• Studies such as Janevic’s research on Romani women (45) and similar work with Indigenous women (3,31,67,68), provide significant insights into the influence of racism on maternal healthcare access. However, these studies often neglect a crucial aspect: the intersection of racism with various identities, including gender and economic class. Despite its importance, the impact of this intersectionality on maternal healthcare access is frequently underemphasized (19,69). Maternal health research (70,71) underscores the significance of intersectionality, as it explores how social identities intersect to shape experiences, exacerbate healthcare disparities, and reveal interconnected systems of oppression (19). Hence, in alignment with Jones’s ‘Three Levels of Racism’ theory (22), the current study utilized intersectionality (19) as an analytical framework. It facilitates a clear understanding of how racism intersects with gender and economic class, leading to diverse experiences of discrimination that influence maternal healthcare access (19,69,72). This approach enhances the comprehension of healthcare inequalities and provides valuable insights for addressing the impact of racism on Garo Indigenous women’s access to maternal healthcare.<br>• I merge the ‘positionality section’ with setting.<br>• This strengthened trust and credibility between the research assistants and participants.

	• Dear respected reviewer, I do not mean "to the participation of the midwife ". I revised the sentences as follow to ensure clarity: Another participant also mentioned that culturally inappropriate care includes not being able to meet the birth attendant before delivering in a hospital, which goes against their cultural norms and expectations.<br>• Dear respected reviewer, I removed this sentence: "This participant expresses concern about the absence of Garo female doctors, as seeking care from a non-Garo male doctor goes against their cultural norms of not sharing personal health problems with male doctors".<br>• Dear respected reviewer, in the revised manuscript I tried by best to differentiate author's voice from the participants.<br>• Dear respected reviewer, I removed this fragmented quote: "I don't know. Probably would have sent me to a free hospital maybe" (L7, Low-class, Age-27).

I trust that these alterations have enhanced the overall quality and coherence of the manuscript, aligning it more closely with the standards of the journal.

Once again, thank you for your time, expertise, and commitment to improving the research. Your feedback has been instrumental in strengthening this work.

Kind Regards.

The Author
