# Supplementary material for: Racism and access to maternal health care among garo indigenous women in Bangladesh: A qualitative descriptive study
Source: PLoS One. 2023 Nov 30;18(11):e0294710. doi: 10.1371/journal.pone.0294710 (PMC10688635; doi:10.1371/journal.pone.0294710)
Supplement: Attachment — Submitted filename: PONE-D-22-34953_R3_rev.pdf [file pone.0294710.s009.pdf]

PLOS ONE

Racism and Access to Maternal Health care Among Garo Indigenous Women in Bangladesh: A Qualitative Descriptive Study

--Manuscript Draft--

Manuscript Number:	PONE-D-22-34953R3
Article Type:	Research Article
Full Title:	Racism and Access to Maternal Health care Among Garo Indigenous Women in Bangladesh: A Qualitative Descriptive Study
Short Title:	Racism and Access to Maternal Health care Among Garo Indigenous Women in Bangladesh
Corresponding Author:	Suban Kumar Chowdhury, MSc in Gender and Development Studies<br>University of Rajshahi<br>Rajshahi, BANGLADESH
Keywords:	Garo Women; racism; Discrimination; Maternal Health.
Abstract:	Racism as social determinant of health significantly affects Indigenous women's maternal healthcare access. This study uses Jones's 'Three Levels of Racism' theory and an intersectional lens to explore how racism shapes the experience of maternal health care access among Garo Indigenous women in Bangladesh. Semi-structured interviews were conducted with 24 women of diverse backgrounds and pregnancy statuses using snowball sampling. Thematic analysis, incorporating inductive and deductive approaches, was employed for data analysis. The findings reveal a significant deviation from Jones's theory regarding the level of internalized racism within the specific context of Garo Indigenous women's experiences. Jones's theory usually focuses on how racism is internalized due to institutional and personally-mediated factors. On the contrary, this study uncovers a unique theme: 'women agency.' This theme emerges as a robust response among the Garo Indigenous women to their encounters with institutional and personally-mediated racism, highlighting their cultural resistance and resilience. The findings suggest that the complex relationship between these two forms of racism contributes to the strengthening of agency among Garo Indigenous women. Their agency stems from avoiding hospitals that disrespect their culture, manifesting their cultural resistance practice against the encountered racism at the institutional and relational levels. To increase biomedical healthcare access among Garo Indigenous women, it is recommended to address racism through intercultural competency training with a 'cultural humility' approach. This approach would foster inclusivity and empowerment, recognizing the agency of Garo women in healthcare decisions. Additionally, it would facilitate constructive dialogues between clinicians and Garo Indigenous women, acknowledging the shared experiences of exploitation within the latter group.
Order of Authors:	Suban Kumar Chowdhury, MSc in Gender and Development Studies
Opposed Reviewers:	
Response to Reviewers:	Dear esteemed reviewer,<br>I would like to express my sincere gratitude for your invaluable feedback on my manuscript. Your insights and recommendations have been immensely helpful in refining the quality and clarity of the research.<br>I am pleased to inform you that I have diligently addressed each of your suggestions, and the changes have been incorporated into the revised manuscript. To provide a clear overview of the modifications, I have compiled them, as below:<br><br><br>Reviewer's comments on Acknowledgement:<br>I strongly suggest you include at least in this section the acknowledgment of the Garo research assistants, whom not only participated in data collection, but also in the analysis and member checking strategy. Do the same with the teacher.<br>RESPONSE: Dear respected reviewer, thank you for your thoughtful suggestion. In

Powered by Editorial Manager® and ProduXion Manager® from Aries Systems Corporation

response to your recommendation, I have included acknowledgments for both the Garo research assistants and the teacher in the appropriate section of the manuscript. Their significant contributions to data collection, analysis, and member checking have been duly noted. Your feedback is greatly appreciated.

Reviewer's comments on Study procedure:

•Review this phrase: “This study utilized a qualitative descriptive approach, commonly employed in semi-structured interviews”. Do you mean “qualitative descriptive approach, recollecting data with semi-structured interviews”?
•Author should expand the description of the method`s scope and utility, explained by Sandelowski: it aims to explore and describe a situation, generally understudied, as it is lived and reported by the people whom experience it. Remember that this approach has a specific scope. It is exploratory and it generally does not have evidence deep enough to support recommendations (as policies). Researchers discuss and conclude based on the data, However, they may point central phenomena to take into consideration in further studies or policy making.
•Sampling: review the sentence “ The participants were categorized into these three groups based on… “ ¿which groups?.
•Sampling. Table 1.
This table in qualitative descriptive studies is important as it shows the variation of the sampling, highly valued in this kind of research. Variation in the participant´s group assures an expanded exploration of an understudied phenomena. Participant table could be helpful to indicate possible areas or populations that require further research. For this, we find this table in the results section.
•Include in this section (sampling) only table 2, adding all the inclusion criteria.
•As author have made significant improvements in results and discussions highlighting intersectionality relevance, table 1 is more useful in the first part of the results. This table must be clarified as follows.

-Please revise precise data of every participant, as in the current table we have mixed information.
-Briefly explain how Household economic status is determined in your context.
-Clarify what “class five” means in your context ¿basic education or high school?
-Briefly explain what does “Self-educated” mean in Garo women context.
-Pregnancy diversity can be better understood if it is shared how many children have been born and if they are or not currently pregnant.

RESPONSE TO THE REVIEWER'S COMMENTS ON STUDY PROCEDURES:

1.Dear respected reviewer, thank you very much for your valuable comment. I revised the sentences as: This research was guided by the qualitative description approach. Qualitative description is widely used methodological approach, especially within practice disciplines.
2.Dear respected reviewer, I addressed this comment following your guidelines. [please see the ‘procedures’ section of the revised manuscript.
3.Dear respected reviewer, thank you for your feedback. The sentence you mentioned has been revised as follows: "The author categorized their household income into three classes: high, middle, and low, based on their monthly household income in comparison to the national average of monthly household income, which was 15,988 BDT as per Bangladesh Bureau of Statistics (BBS) data."
I believe this revision provides a clearer explanation of how the participants were categorized based on their income levels.
[please see the result section, page 11]
4.Dear respected reviewer, thank you very much for your valuable comment. I have addressed your valuable comments by making the following revisions:
* I have relocated Table 1 to the first part of the results section, as suggested by the reviewer. This adjustment helps emphasize the variation in participant groups and the potential areas for further research, aligning with the goals of qualitative descriptive studies.
* In response to your recommendation, I have included Table 2 in the sampling section, incorporating all the inclusion criteria. This ensures that the sampling section comprehensively presents participant characteristics.

Powered by Editorial Manager® and ProduXion Manager® from Aries Systems Corporation

	-I have revised and provided precise data for each participant, ensuring that the information in the table is clear and well-organized.<br><br>-I have included a brief explanation of how household economic status is determined in the context of the study.<br><br>-I clarified that "class five" in the context refers to primary level of education.<br><br>-I have replaced the term "Self-educated" to illiterate as it will be easier to understand for the reader.<br><br>-I have included information about the number of children each participant has and their pregnancy status to enhance the understanding of pregnancy diversity.<br><br>Reviewer's comments on ANALYSIS SECTION:<br>1.In this new version author reports doing content analysis. Even though this approach is highly used in qualitative descriptive analysis, results are presented in themes. There is a common confusion between content and thematic analysis in qualitative descriptive analysis and needs to be avoided. Vaismoradi, Turunen and Bonda clarify how to address this risk in their paper. Please adjust analysis, briefly highlining the process that makes it content analysis or thematic analysis, to address methodological coherence and transparency in the report.<br>2.Also, clarify if it is a deductive or inductive process. Apparently, the author did a deductive analysis, based on Jones' racism framework, combined with an inductive analysis, as there seem to have emergent categories (not addressed in Jones´ racism theory): Agency; Cultural resistance practice; Community practices of maternal healthcare (traditional medicine practices of maternal health?).<br>RESPONSE TO THE REVIEWER'S COMMENTS OF THE ANALYSIS SECTION:<br>1.Dear respected reviewer, I appreciate your valuable feedback, and I have made the necessary revisions to the analysis section to ensure methodological coherence and transparency. In the revised version, I have explicitly mentioned that I used 'thematic analysis' as the method for analysing the data. This adjustment helps clarify the approach taken in the study, aligning it with the methodological choice and addressing any potential confusion between content and thematic analysis. Thank you for highlighting this important point, and I hope the clarification improves the clarity of the report.<br>2.Dear respected reviewer, thank you for your insightful comments. I have addressed the issue of deductive and inductive analysis in the revised 'Analysis' section of the manuscript. I explicitly mention that I employed both inductive and deductive approaches in a complementary manner.<br>I clarified the rationale behind this choice, explaining that while I initially used a deductive approach based on Jones' racism framework, I also allowed for the emergence of additional categories that were not addressed in Jones' theory. This combination of deductive and inductive analysis enriches the understanding of the data and ensures that both the pre-defined and emergent themes are thoroughly explored.<br>I hope these clarifications provide a more comprehensive view of the analysis process in the study. Thank you for your valuable feedback, which has contributed to improving the rigor and transparency of the research.<br>Reviewer's comments on Trustworthiness:<br>consider include this section in the procedure as you develop multiple strategies to enhance rigor in your context: triangulation between assistants and researcher during analysis; member-checking of results with participants. Also include here positionality of author and the strategies used to enhance cultural sensitivity with the support of Garo women assistants and the community teacher.<br>RESPONSE<br>Dear respected reviewer,<br>Thank you for your suggestion regarding the inclusion of a 'Trustworthiness' section in the procedure. I appreciate your thoughtful input. I want to inform you that I have indeed included a separate section titled 'Trustworthiness' immediately after the 'Analysis' section in the revised manuscript. In this section, I have addressed multiple strategies employed to enhance the rigor of the study.<br>I also merged the section on 'Author's Positionality' with the section titled 'Research Site' to provide a more comprehensive understanding of the context and the strategies

Powered by Editorial Manager® and ProduXion Manager® from Aries Systems Corporation

used to enhance cultural sensitivity with the support of Garo women assistants and the community teacher.

I believe these revisions have strengthened the manuscript, and I hope they align with your expectations regarding the presentation of trustworthiness measures. Your feedback has been instrumental in refining the study, and I am grateful for your guidance.

Reviewer's comments on the RESULT SECTION

Author improved significantly the results report. Fragments included about traditional medicine practices, and about women agency and trust in their culture are valuable. However, because of the way results are presented, there is a major theoretical incoherence that could have a simple solution:
1.How does agency, Cultural resistance practice. and Community practices of maternal healthcare constitute internalized racism? It is exactly the opposite of internalized racism. If women had internalized racism (as in the Mexican study you cite on discussion), they would prefer to go the hospitals instead of their midwifes, they would not be proud of their culture, they would even believe they deserve bad treatment.
2.This emergent theme is crucial to understand and support Garo Culture and Garo women strategies to face racism at bio-medical centers. Protecting Garo Culture and women agency is as necessary as transforming the biomedical healthcare institution, from the theoretical framework reported. I strongly suggest to create an additional theme grouping these sub-themes, related to cultural strength or resistance towards racism.
3.Even though the author clearly has the intention to support Garo Women and Garo culture from racism, the model continues to suggest that more dependency on the cultural practices of maternal healthcare is a negative effect of institutional racism and internalized racism. therefore, it suggests cultural strength cause poor access to biomedical maternity care. In this regard consider that:
-there is no sufficient data evidence that describe how more dependency on traditional medicine leads to poor access to biomedical maternity care.
-Presenting a model suggesting these relations may contribute to enhance institutional racism, if the reader misunderstand that traditional medicine and cultural practices (and not racism directly) are the reason Garo women do not seek bio-medical maternal health care.
-The solution would be to adjust the model, identifying the relations reported by Garo women only:
a) Poor access to biomedical maternal healthcare is rooted in institutional and interpersonal; b) there does not seem to be reports of internalized racism. If so, remove it from the Figure. c). the emergent theme (related to strong Cultural traditional-medicine practices / women agency) may work as a resistance towards both types of racism.
4. Author may want to attenuate the affirmation of the hospital exploiting Garo women, taking into account a) ¿is it an experience of all the women in the study? (if so, author should report the abuse, if not, author could report the experience of some of the women with some of the staff) b) does the author want the hospital to be open to this study suggestions, and not defensive towards the claims?
•P. 16: Adjust this sentence: “This illustrates a link to personally-mediated ethnocentrism, as presented in the following sub-section”. (Racism?)

Reviewer's comments Discussion and Conclusion Section:

1.Results do not suggest how to solve the problem (implementing intercultural training programs) and it is not the scope of this kind of study. How ever, author can attenuate the affirmation, focusing on concluding facts that the study shows based on the evidence of the reports of Garo women: there is strong racism at different levels, and it strongly compromises biomedical healthcare access. Garo women count on a strong traditional medicine system to attend maternal healthcare. Garo women agency derives in their decision to avoid hospitals were they and their culture is mis-respected. Therefore, to increase biomedical healthcare access for Garo women racism needs to be prevent in the institutional and relational level. Author then can suggests the models to explore for future policy-making.
2.In discussion and abstract the author refers to Inclusive approaches like ‘Community

Powered by Editorial Manager® and ProduXion Manager® from Aries Systems Corporation

Controlled Health Services' and 'Garo liaison officers'. These programs have not been previously presented, so the relation with result is unclear. Consider to present as examples, or expand it in the introduction.

Response to the Reviewer's comments Discussion and Conclusion Section:
1.Dear respected reviewer,
I want to express my sincere gratitude for your insightful comments and recommendations. Your feedback has been instrumental in refining the manuscript, and I truly appreciate your time and expertise. I have incorporated your suggestions into the discussion section to provide a more nuanced and evidence-based conclusion. In light of your input, I have adjusted the discussion as follows:
Based on the evidence from the reports of Garo women, it is evident that racism exists at different levels and significantly compromises their access to biomedical healthcare. Garo women have a strong reliance on traditional medicine for maternal healthcare. Their agency is rooted in their decision to avoid hospitals where they and their culture are not respected. Therefore, to enhance biomedical healthcare access for Garo women, it is imperative to address racism at both the institutional and relational levels. "then I suggest for implementing intercultural competency training program (CICTP) for the clinicians".

2.Dear respected reviewer,
Thank you for your comment regarding the mention of 'Community Controlled Health Services' and 'Garo liaison officers' in the discussion and abstract sections of the manuscript. I appreciate your feedback and have made the necessary revisions based on your suggestion.
I have removed the policy recommendations related to these inclusive approaches from the discussion section as they were not adequately introduced or connected to the study's findings. Instead, I have focused on highlighting the broader implications of the study's results and the need for addressing racism at different levels within healthcare institutions, as per your earlier recommendations. This change was made to ensure clarity and relevance in the discussion section.
Once again, I want to express my gratitude for your valuable input, which has contributed to improving the coherence and effectiveness of the manuscript.

Other minor revisions as suggested by the reviewer:
Here are some fragments that need writing revisions, but a final review of the writing is required.
•Revise last phrase on p.4 "It is argues that a consideration of the women's varied social contexts and diverse identities is essential in comprehending their maternity care experiences (44)."
•Revise redaction of Jones's 'three levels of racism' new paragraph in introduction.
•P. 5. Revise this redaction: "Given such strength, Jones's theory, evident in studies like Janevic's on Romani women(43) and others on Indigenous women (3,39,64), highlights racism's impact on maternal healthcare…" and "The Upazila hospital has medical staff, including non-Garo male gynecologist, with most of the pregnant patients seeking care there are not Garo women".
•Positionality: Consider including this section with no title, but merged with the setting or in the suggested section of trustworthiness. In this phrase "The author has a distinct positionality concerning the research site and the participants" what do you mean by distinct positionality?
•p.9 This process enhanced trust and credibility between research assistants and participants during data collection, with the research assistants reporting to the author.
•P. 14. "As another participant shared, the issue of culturally inappropriate care extends to the midwifery meeting". Do you mean "to the participation of the midwife "?
•P14. This explanation may be removed as it is contains in previous paragraph: "This participant expresses concern about the absence of Garo female doctors, as seeking care from a non-Garo male doctor goes against their cultural norms of not sharing personal health problems with male doctors".
•P.16. Differentiate researchers' voice from participants: "Distance cost isn't all. Hospitals want money. Despite their claim, they don't charge 5 Taka (Bangladeshi currency). They demand more. Traveling makes you hungry, but food is expensive. We must spend all we earn if we go. She also adds, we don't control travel and treatment costs. They ignore our issue. They said 'you have to follow the hospital rules' whenever we told them our problem. What else can we do but avoid it? (L2, Middle-class, Age-

Powered by Editorial Manager® and ProduXion Manager® from Aries Systems Corporation

	30).<br>•P.21. Consider removing this part of the fragment that does not add much to what is being shown: “I don’t know. Probably would have sent me to a free hospital maybe” (L7, Low-class, Age-27).<br><br>Author's response:<br>•These studies often lack a context-specific understanding of intersectionality and overlook the perspectives of Indigenous women who face compounded prejudice and implicit bias due to various aspects of their identity such as gender and economic class. To comprehensively grasp Indigenous women’s maternity care experiences, it is imperative to consider their diverse identities within distinct social contexts.<br>• The study employs Jones’s ‘three levels of racism' framework (22) along with Crenshaw’s intersectional lens (19) to frame and analyze the themes emerged from participants’ discussion. Jones’s model categorizes racism into three levels: institutional, personally-mediated, and internalized (22). This breakdown helps providing a deeper understanding of the complex impact of racism on maternal healthcare access. Unlike models that primarily focus on the social construction of race or ethnicity and the structural factors perpetuating racial inequalities (64–66), Jones’s comprehensive approach offers a holistic understanding. It suggests that institutional and personally-mediated racism contribute to the internalization of racism within the context of maternal healthcare access (22,28). Given its strong theoretical foundation, Jones’s model is particularly relevant for our study.<br>• Studies such as Janevic’s research on Romani women (45) and similar work with Indigenous women (3,31,67,68), provide significant insights into the influence of racism on maternal healthcare access. However, these studies often neglect a crucial aspect: the intersection of racism with various identities, including gender and economic class. Despite its importance, the impact of this intersectionality on maternal healthcare access is frequently underemphasized (19,69). Maternal health research (70,71) underscores the significance of intersectionality, as it explores how social identities intersect to shape experiences, exacerbate healthcare disparities, and reveal interconnected systems of oppression (19). Hence, in alignment with Jones’s ‘Three Levels of Racism’ theory (22), the current study utilized intersectionality (19) as an analytical framework. It facilitates a clear understanding of how racism intersects with gender and economic class, leading to diverse experiences of discrimination that influence maternal healthcare access (19,69,72). This approach enhances the comprehension of healthcare inequalities and provides valuable insights for addressing the impact of racism on Garo Indigenous women’s access to maternal healthcare.<br>•I merge the ‘positionality section’ with setting.<br>•This strengthened trust and credibility between the research assistants and participants.<br>•Dear respected reviewer, I do not mean “to the participation of the midwife ”. I revised the sentences as follow to ensure clarity: Another participant also mentioned that culturally inappropriate care includes not being able to meet the birth attendant before delivering in a hospital, which goes against their cultural norms and expectations.<br>•Dear respected reviewer, I removed this sentence: “This participant expresses concern about the absence of Garo female doctors, as seeking care from a non-Garo male doctor goes against their cultural norms of not sharing personal health problems with male doctors”.<br>•Dear respected reviewer, in the revised manuscript I tried by best to differentiate author’s voice from the participants.<br>•Dear respected reviewer, I removed this fragmented quote: “I don’t know. Probably would have sent me to a free hospital maybe” (L7, Low-class, Age-27).<br><br>I trust that these alterations have enhanced the overall quality and coherence of the manuscript, aligning it more closely with the standards of the journal.<br>Once again, thank you for your time, expertise, and commitment to improving the research. Your feedback has been instrumental in strengthening this work.<br><br>Kind Regards.<br>The Author
**Additional Information:**	
**Question**	**Response**

Powered by Editorial Manager® and ProduXion Manager® from Aries Systems Corporation

**Financial Disclosure**<br><br>Enter a financial disclosure statement that describes the sources of funding for the work included in this submission. Review the submission guidelines for detailed requirements. View published research articles from *PLOS ONE* for specific examples.<br><br>This statement is required for submission and **will appear in the published article** if the submission is accepted. Please make sure it is accurate.<br><br>**Unfunded studies**<br>Enter: *The author(s) received no specific funding for this work.*<br><br>**Funded studies**<br>Enter a statement with the following details:<br>• Initials of the authors who received each award<br>• Grant numbers awarded to each author<br>• The full name of each funder<br>• URL of each funder website<br>• Did the sponsors or funders play any role in the study design, data collection and analysis, decision to publish, or preparation of the manuscript?<br>• **NO** - Include this sentence at the end of your statement: *The funders had no role in study design, data collection and analysis, decision to publish, or preparation of the manuscript.*<br>• **YES** - Specify the role(s) played.<br><br>* typeset	The author received no specific funding for this research.
**Competing Interests**<br><br>Use the instructions below to enter a competing interest statement for this submission. On behalf of all authors, disclose any competing interests that could be perceived to bias this work—acknowledging all financial support and any other relevant financial or non-financial competing interests.<br><br>This statement is required for submission	The authors have declared that no competing interests exist.

Powered by Editorial Manager® and ProduXion Manager® from Aries Systems Corporation

and **will appear in the published article** if the submission is accepted. Please make sure it is accurate and that any funding sources listed in your Funding Information later in the submission form are also declared in your Financial Disclosure statement.<br><br>View published research articles from *PLOS ONE* for specific examples.<br><br>**NO authors have competing interests**<br><br>Enter: *The authors have declared that no competing interests exist.*<br><br>**Authors with competing interests**<br><br>Enter competing interest details beginning with this statement:<br><br>*I have read the journal's policy and the authors of this manuscript have the following competing interests: [insert competing interests here]*<br><br>* typeset	
**Ethics Statement**<br><br>Enter an ethics statement for this submission. This statement is required if the study involved:<br><br>• Human participants<br>• Human specimens or tissue<br>• Vertebrate animals or cephalopods<br>• Vertebrate embryos or tissues<br>• Field research<br><br>Write "N/A" if the submission does not require an ethics statement.<br><br>General guidance is provided below. Consult the submission guidelines for detailed instructions. **Make sure that all information entered here is included in the Methods section of the manuscript.**	As this research is the partial part of the author's MSc thesis project which has done during pursuing MSc degree from the Asian Institute of Technology (AIT), ethics approval has received from AIT ethics committee.

**Format for specific study types**<br><br>**Human Subject Research (involving human participants and/or tissue)**<br>• Give the name of the institutional review board or ethics committee that approved the study<br>• Include the approval number and/or a statement indicating approval of this research<br>• Indicate the form of consent obtained (written/oral) or the reason that consent was not obtained (e.g. the data were analyzed anonymously)<br><br>**Animal Research (involving vertebrate animals, embryos or tissues)**<br>• Provide the name of the Institutional Animal Care and Use Committee (IACUC) or other relevant ethics board that reviewed the study protocol, and indicate whether they approved this research or granted a formal waiver of ethical approval<br>• Include an approval number if one was obtained<br>• If the study involved *non-human primates*, add *additional details* about animal welfare and steps taken to ameliorate suffering<br>• If anesthesia, euthanasia, or any kind of animal sacrifice is part of the study, include briefly which substances and/or methods were applied<br><br>**Field Research**<br><br>Include the following details if this study involves the collection of plant, animal, or other materials from a natural setting:<br>• Field permit number<br>• Name of the institution or relevant body that granted permission	
**Data Availability**<br><br>Authors are required to make all data underlying the findings described fully available, without restriction, and from the time of publication. PLOS allows rare exceptions to address legal and ethical concerns. See the PLOS Data Policy and FAQ for detailed information.	Yes - all data are fully available without restriction

A Data Availability Statement describing where the data can be found is required at submission. Your answers to this question constitute the Data Availability Statement and **will be published in the article**, if accepted.<br><br>**Important:** Stating 'data available on request from the author' is not sufficient. If your data are only available upon request, select 'No' for the first question and explain your exceptional situation in the text box.<br><br>Do the authors confirm that all data underlying the findings described in their manuscript are fully available without restriction?	
**Describe where the data may be found in full sentences. If you are copying our sample text, replace any instances of XXX with the appropriate details.**<br><br>• If the data are **held or will be held in a public repository**, include URLs, accession numbers or DOIs. If this information will only be available after acceptance, indicate this by ticking the box below. For example: *All XXX files are available from the XXX database (accession number(s) XXX, XXX.).*<br>• If the data are all contained **within the manuscript and/or Supporting Information files**, enter the following: *All relevant data are within the manuscript and its Supporting Information files.*<br>• If neither of these applies but you are able to provide **details of access elsewhere**, with or without limitations, please do so. For example:<br><br>*Data cannot be shared publicly because of [XXX]. Data are available from the XXX Institutional Data Access / Ethics Committee (contact via XXX) for researchers who meet the criteria for access to confidential data.*<br><br>*The data underlying the results presented in the study are available from (include the name of the third party*	All relevant data are within the manuscript and its Supporting Information files.

and contact information or URL).<br>• This text is appropriate if the data are owned by a third party and authors do not have permission to share the data.<br><br>* typeset	
Additional data availability information:	Tick here if your circumstances are not covered by the questions above and you need the journal's help to make your data available.

Powered by Editorial Manager® and ProduXion Manager® from Aries Systems Corporation

Dear respected academic editor,

I wish to extend my heartfelt appreciation for your ongoing support.

I am delighted to inform you that I have thoroughly addressed all the valuable suggestions provided by the reviewer(s), and I have successfully incorporated the recommended changes into the revised manuscript. Your supports have been instrumental in improving the quality of the paper.

I believe that the revised version of the manuscript will be of interest to the readers of your journal. Also, I declare that this manuscript is original, has not been published before and is not currently being considered for publication elsewhere. I know of no conflict of interest associated with this publication.

Thank you, once again.

Kind Regards.

The Author

Manuscript
Click here to access/download;Manuscript;Manuscript.docx

Racism and Access to Maternal Health care Among Garo Indigenous Women in Bangladesh: A Qualitative Descriptive Study

Suban Kumar Chowdhury[1]

[1] Department of International Relations, University of Rajshahi, Bangladesh.
*Suban Kumar Chowdhury
E-mail: skc_ruir@ru.ac.bd

Author's contributions

The author's interest in Indigenous women's maternal health led to this research. He reviewed relevant literature, designed research instruments, and served as the principal researcher, writing both the initial and final manuscript drafts.

Acknowledgement

The author would like to express gratitude to the anonymous reviewers for their valuable input. This article is based on the author's MSc thesis work, which was supervised by Dr. Joyee S. Chatterjee, Program Chair of Gender and Development Studies at AIT. The author extends heartfelt thanks to Dr. Chatterjee. Special recognition is given to Shila Gagra for her assistance in developing culturally relevant interview questions and recruiting four female research assistants: Manisha, Namita, Malita, and Tomita. The author sincerely appreciates their active supports throughout the research. Additionally, the author acknowledges Dr. Eisuke Saito, Dr. Anne Keary (Faculty at Monash University, Australia), and Dr. Melissa Barnes (La Trobe University, Australia) for their guidance on using inductive and deductive approaches in research.

Racism and Access to Maternal Health care Among Garo Indigenous Women in Bangladesh: A Qualitative Descriptive Study

Abstract

Racism as social determinant of health significantly affects Indigenous women's maternal healthcare access. This study uses Jones's 'Three Levels of Racism' theory and an intersectional lens to explore how racism shapes the experience of maternal health care access among Garo Indigenous women in Bangladesh. Semi-structured interviews were conducted with 24 women of diverse backgrounds and pregnancy statuses using snowball sampling. Thematic analysis, incorporating inductive and deductive approaches, was employed for data analysis. The findings reveal a significant deviation from Jones's theory regarding the level of internalized racism within the specific context of Garo Indigenous women's experiences. Jones's theory usually focuses on how racism is internalized due to institutional and personally-mediated factors. On the contrary, this study uncovers a unique theme: 'women agency.' This theme emerges as a robust response among the Garo Indigenous women to their encounters with institutional and personally-mediated racism, highlighting their cultural resistance and resilience. The findings suggest that the complex relationship between these two forms of racism contributes to the strengthening of agency among Garo Indigenous women. Their agency stems from avoiding hospitals that disrespect their culture, manifesting their cultural resistance practice against the encountered racism at the institutional and relational levels. To increase biomedical healthcare access among Garo Indigenous women, it is recommended to address racism through intercultural competency training with a 'cultural humility' approach. This approach would foster inclusivity and empowerment, recognizing the agency of Garo women in healthcare decisions. Additionally, it would facilitate constructive

dialogues between clinicians and Garo Indigenous women, acknowledging the shared experiences of exploitation within the latter group.

Introduction

Racism, defined as unfair treatment based on ethnic or cultural origin (1), significantly affects the maternal health outcomes of Indigenous women (2–5). While well-documented (3,5–7), its diverse impact on Indigenous women's access to maternal healthcare remains underexplored (8). Research from various countries indicates higher rates of maternal morbidity and mortality associated with racism (9–11). Instances of racial discrimination against Indigenous women by healthcare providers are evident in both India and the United States (12–15). Biomedical maternity care often exhibits racial bias and cultural insensitivity, resulting in trauma, mistrust, and reduced motivation among Indigenous women to seek healthcare (6,16). The intersection of gender, economic class, and other identities with racism further complicates Indigenous women's healthcare experiences (17–19), highlighting racism as a significant social determinant influencing their access to maternal healthcare (5,20). Racism as a system of privilege and oppression operates across institutional, personally-mediated, and internalized levels, resulting in an unequal distribution of power (21). This systemic racism perpetuates stereotypes, bias, and unequal access to resources (21,22), highlighting the absence of cultural humility approaches at the institutional and relational levels (23). Consequently, racism-driven discriminations have a substantial impact on maternal healthcare access (22,24–28). The patient-perceived discrimination scale is a valuable tool for investigating experiences within Indigenous communities (28). Additionally, understanding racially biased provider attitudes, particularly implicit bias, enhances awareness of interpersonal discrimination (29–31).

Racism against Indigenous identity is gaining recognition (32) but remains overlooked as a health issue in South Asian countries, including Bangladesh (33). Research reveals that racism significantly impacts Indigenous South Asian women's maternity care access, leading to health disparities and inadequate support (34–36). In Bangladesh, limited research focuses on Indigenous women's experiences with racism and maternity care (37,38), in contrast to global research in countries like the United States, Mexico, and India (9,31). Most research on healthcare access and racism centers on patients' perceptions of provider bias or implicit discrimination (39–44), with few exploring its direct health effects (39,45). These studies often lack a context-specific understanding of intersectionality and overlook the perspectives of Indigenous women who face compounded prejudice and implicit bias due to various aspects of their identity such as gender and economic class. To comprehensively grasp Indigenous women's maternity care experiences, it is imperative to consider their diverse identities within distinct social contexts (46).

This study examines how racism impacts Garo Indigenous women's experience of accessing biomedical maternal healthcare in Bangladesh. The Garo community is one of the largest Indigenous communities in Bangladesh (47), and one of the few close-knit communities worldwide (48). They follow a unique 'matrilineal-matrilocal' family structure (48). In this arrangement, family lineage and property are traced through the mother's line (49). Newly married couples reside with or near the wife's family, which grants women higher status and authority within the household and community (50). Since 2011, however, the government's postcolonial policies labeled the Garo community as 'tribal' instead of 'Indigenous' based on their culture and language, resulting in hierarchical distinctions, restricted access to rights and resources, and the reinforcement of cultural inferiority and colonial exclusionary practices (51–59). Despite their limited access to biomedical maternity care, racial biases in healthcare remain understudied (60–

62). Additionally, the scarcity of disaggregated data on indigenous women's healthcare access (63) underscores the necessity of this study.

The study employs Jones's 'three levels of racism' framework (22) along with Crenshaw's intersectional lens (19) to frame and analyze the themes emerged from participants' discussion. Jones's model categorizes racism into three levels: institutional, personally-mediated, and internalized (22). This breakdown helps providing a deeper understanding of the complex impact of racism on maternal healthcare access. Unlike models that primarily focus on the social construction of race or ethnicity and the structural factors perpetuating racial inequalities (64–66), Jones's comprehensive approach offers a holistic understanding. It suggests that institutional and personally-mediated racism contribute to the internalization of racism within the context of maternal healthcare access (22,28). Given its strong theoretical foundation, Jones's model is particularly relevant for our study.

Studies such as Janevic's research on Romani women (45) and similar work with Indigenous women (3,31,67,68), provide significant insights into the influence of racism on maternal healthcare access. However, these studies often neglect a crucial aspect: the intersection of racism with various identities, including gender and economic class. Despite its importance, the impact of this intersectionality on maternal healthcare access is frequently underemphasized (19,69). Maternal health research (70,71) underscores the significance of intersectionality, as it explores how social identities intersect to shape experiences, exacerbate healthcare disparities, and reveal interconnected systems of oppression (19). Hence, in alignment with Jones's 'Three Levels of Racism' theory (22), the current study utilized intersectionality (19) as an analytical framework. It facilitates a clear understanding of how racism intersects with gender and economic class, leading to diverse experiences of discrimination that influence maternal healthcare access

(19,69,72). This approach enhances the comprehension of healthcare inequalities and provides valuable insights for addressing the impact of racism on Garo Indigenous women's access to maternal healthcare.

Materials and methods

Settings

The study was conducted in Kalmakanda, a sub-district in northern Bangladesh. It primarily focused on the Garo people living in the unions of Lengura and Nazirpur (See Map 1) (73). Garo Indigenous women in these unions face challenges as they have limited access to maternal healthcare facilities, lacking Union sub-centers and community clinics (74,75). While the Upazila hospital has medical staff, including non-Garo male gynecologists, it is noteworthy that few Garo women seek care there. This situation prompts questions about the potential influence of racism in this context.

Map 1: Study area

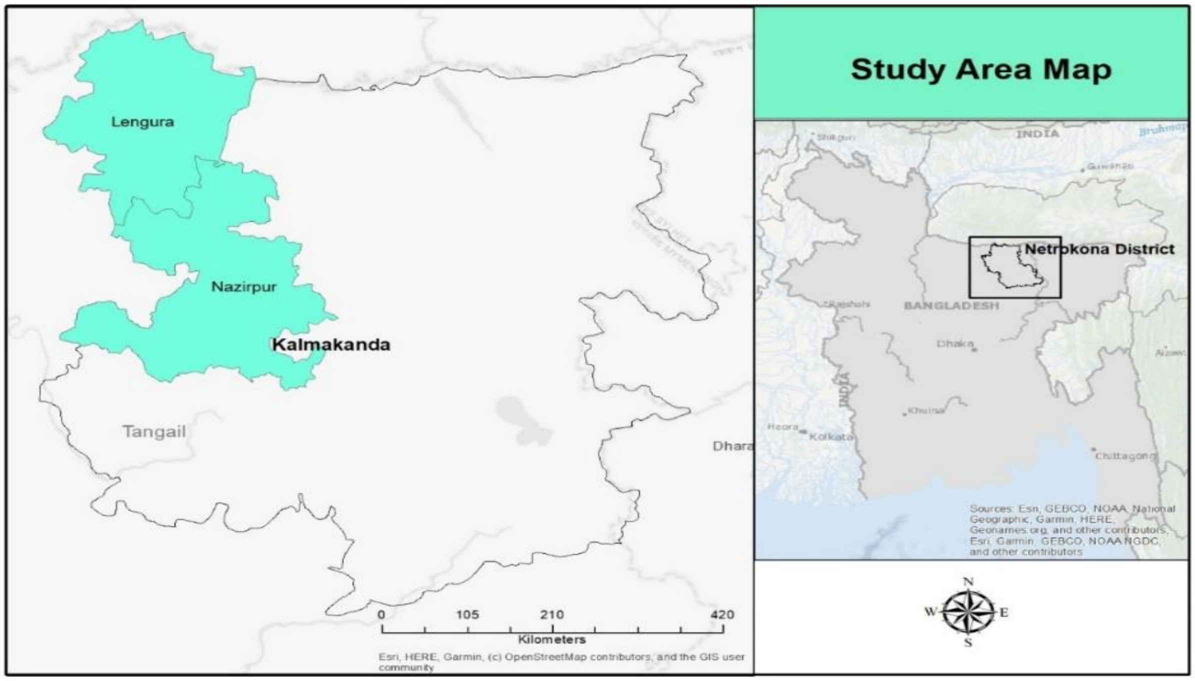

The author of the study is a male and holds the role of a researcher and primary investigator. He is from the mainstream non-Garo community in Bangladesh living outside of the study area. This makes him stranger when it comes to the place where the research was done and the participants who were part of it. The research site involves interactions with Garo Indigenous women seeking maternal health care. Being an outsider to the Garo community and coming from a different culture, the author emphasized the significance of culturally relevant approaches when engaging with the research subjects-Garo Indigenous women. To achieve this, the author hired female Garo research assistants on a paid basis to collect data since they have a cultural affinity with the participants, helping to establish cultural understanding and trust. This decision was based on the knowledge that cultural insiders could better connect with the participants and conduct the research responsibly (76). The author conducted three training sessions covering roles, rapport-building, and ethical considerations. Practical training included two demo interviews, enabling research assistants to gain the necessary skills for conducting interviews aligned with research objectives.

Study Procedures

This research was guided by the qualitative description approach (77). Qualitative description is widely used methodological approach, especially within practice disciplines (78). This approach was selected for this study as the technique of inquiry because it is typically applied to research driven by exploratory questions (79). The lack of in-depth research on maternal healthcare access among Garo indigenous women led to a need for an in-depth exploratory study. Qualitative description is notable for employing everyday language to create detailed summaries of findings (80). It facilitates detailed summarization of experiences or phenomena (80), assisting knowledge development in emerging fields (81). Given the nascent state of research on Indigenous women's

maternal healthcare access in Bangladesh (61,82), the outcomes of this qualitative description form a crucial basis for future research aimed at a broader theoretical understanding of the experience of racism among Indigenous women accessing maternal healthcare in Bangladesh.

Sampling and recruitment

The study used intersectionality-informed methods (83) to ensure a diverse sample by considering participants' overlapping identities and experiences. This approach captured the complexities of their lives and how social factors shape their perspectives (83). Thus, beyond traditional categories, the selection process considered identities as multidimensional, leading to a comprehensive understanding of participants' perspectives (19). Purposive and snowball sampling techniques, as the intersectionality-informed methods (84), were used to enroll 24 Garo Indigenous women (n=12 from Lengura Union and n=12 from Nazirpur Union) (85,86). It helps to address the challenges in identifying pregnant and new mothers (85). By combining purposive and snowball sampling methods and considering factors such as age, literacy, economic class, and pregnancy conditions (see Table 1 for inclusion criteria), the study ensured diversity. Also, it helps to represented the diverse perspectives of the participants. The author recognized historical challenges faced by Indigenous members in proving their Indigenous membership and did not require enrollment proof (87).

Table 1: Research area, number of IDI participants, and their inclusion criteria.

IDI respondents							
Area			***Number***	***Selection criteria***			
Netrokona District	Kalmakanda Upazila	Lengura Union	n=12	Age range	Household economic status	Educational level	Pregnancy diversity
				19-30	-Low-class<br>-Middle-Class<br>-High-class	Illiterate and Literate	The Garo women who were pregnant or had childbirth within 1 year before the date of taking the interview date
		Nazirpur Union	n=12				

Development of the Interview Guide

The author collaborated with Shila Gagra, a Garo school teacher in the study area during the study. Shila voluntarily provided valuable input in developing interview questions, ensuring cultural relevance. She also assisted in recruiting four Garo female research assistants and disseminating study findings.

Data Collection Procedure

Data were collected from February to March 2021 through semi-structured interviews and open-ended questionnaires, exploring maternal healthcare experiences and practices. Interviews lasted at least 50 minutes, were audio-recorded, and field notes were taken. The comprehensive training of the research assistants and their efforts in the structured data collection process ensured the acquisition of detailed and valuable insights into the respondents' experiences with maternal healthcare access. To ensure rigorous data collection, research assistants followed strategies of encouraging participants for open expression (88). They performed member-checking with willing participants, offering to provide a summary of findings for their review and feedback. This strengthened trust and credibility between the research assistants and participants.

Data Analysis

Virginia Braun and Victoria Clarke's six-step framework was used to guide the thematic analysis in this research, incorporating both inductive and deductive approaches (89). The rationale for employing both inductive and deductive methods lie in their complementary nature, as they allow for the use of theory to understand the data while also permitting the data to generate key themes/concepts that speak to the theory. Integration of both methods allowed an evaluation of

Jones's 'Three Levels of Racism' theory in the context of Garo Indigenous women, leading to novel insights that contribute to existing knowledge.

To enhance both the reliability and coherence of the findings, collaborative efforts were made with research assistants throughout the data analysis process. The interviews underwent transcription in Bengali, followed by translation into English, and cross-checking by both the author and research assistants. Immersion within the transcripts aimed to achieve a comprehensive understanding of racial experiences in the context of Jones's 'Three Levels of Racism' theory, with an openness to emerging themes. An inductive approach was employed to generate initial codes within each transcript, forming the basis for a systematic search for themes guided by Jones's theory, validated through collaboration with research assistants. These themes were defined, named, and aligned with Jones's theory, recognizing the emergence of new themes. The themes were presented to the participating Garo Indigenous women to ensure their alignment with the participants' perspective and viewpoint. Finally, the author writes the result section using the identified themes and relevant verbatim quotes from the transcripts. A summary of the results, written in Mandi language through collaborative efforts with research assistants, is shared with participants to gather their feedback for the final draft. Once consent is obtained, the author revisits the study area, invites all participants, and presents the final version of the research findings. Shila Gagra and research assistants assist the author in this process.

Trustworthiness

The triangulation between assistants and author during analysis; re-checking the codes and themes, member-checking of results with participants which has been abstracted thus far demonstrates the author's effort to ensure trustworthy of the research findings.

Ethical Consideration

This study, conducted as part of the author's MSc thesis at the Asian Institute of Technology (AIT), Thailand, received ethics approval from the AIT Ethics Committee. There was not local ethics committee from which the author required to seek approval. Before conducting interviews, participants and their husbands, as the local guardian, were informed about the research and provided verbal consent, following the approved approach. Research assistants from the Garo community established rapport and addressed concerns directly, leading to higher-quality approval. The prioritization of oral consent enhanced participant comprehension, engagement, and well-being, as reported by the research assistants, ultimately increasing the validity and reliability of the study. Confidentiality and voluntary participation were emphasized throughout the data collection process, ensuring participants were free to withdraw without consequences. Pseudonyms were utilized to protect privacy.

Results

Participants' characteristics

In-depth interviews (IDIs) were conducted with twenty-four Garo women. They typically expressed dissatisfaction with hospital-level healthcare access. Thematic saturation was achieved after nineteen IDIs, but to delve deeper, five more IDIs were conducted. The participants actively engaged and interacted effectively with the research assistants. Their ages ranged from 19 to 30 years, and they had diverse educational backgrounds and income levels. Most of them had at least one child. The author categorized their household income into three classes: high, middle, and low, based on their monthly household income in comparison to the national average of monthly household income, which was 15,988 BDT as per Bangladesh Bureau of Statistics (BBS) data (90).

Table 2: Participant profiles with pseudonyms from Lengura Union

Place of residence	Participant	Age	Household economic Status	Educational Level	Pregnancy diversity
Lengura Union	L1	21	Low-class	Illiterate	She had 2 children and was in the third trimester of her pregnancy during the interview.
	L2	30	Middle-Class	Illiterate	She had 4 children, and her most recent childbirth occurred within eight months before the interview date.
	L3	22	Middle-Class	Illiterate	She had 3 children and was in the first trimester of her pregnancy during the interview.
	L4	25	High-Class	She finished primary school education	She had 1 child and was in the first trimester of her pregnancy during the interview.
	L5	19	Middle-Class	Illiterate	She was in the second trimester of her pregnancy during the interview and had no previous experience of pregnancy
	L6	20	Low-Class	Illiterate	She had 1 child and was in the second trimester of her pregnancy during the interview.
	L7	27	Low-Class	Illiterate	She had 3 children, and her most recent childbirth occurred within five months before the interview date.
	L8	20	Middle-Class	Illiterate	She had 2 children and was in the second trimester of her pregnancy during the interview.
	L9	21	High-class	She finished primary school education	Had no previous experience of pregnancy and her most recent childbirth occurred within two months before the interview date.
	L10	23	Low-Class	Illiterate	She had 2 children, and her most recent childbirth occurred within nine months before the interview date.
	L11	19	High-Class	She finished primary school education	She had no prior pregnancy experience was in the second trimester of her pregnancy during the interview.
	L12	26	Low-Class	Illiterate	She had 3 children and was in the third trimester of her pregnancy during the interview.

Table 3: Participant profiles with pseudonyms from Nazirpur Union

Place of residence	Participant	Age	Household economic Status	Educational Level	Pregnancy diversity
Nazirpur Union	N1	20	Middle-Class	Illiterate	She had 1 child and was in the second trimester of her pregnancy during the interview.
	N2	20	Middle-Class	Illiterate	Had no previous experience of pregnancy and her most recent childbirth occurred within four months before the interview date.
	N3	22	High-Class	She finished primary school education	She had 1 child and was in the third trimester of her pregnancy during the interview.
	N4	20	Middle-Class	Illiterate	Had no previous experience of pregnancy and her most recent childbirth occurred within three months before the interview date.
	N5	24	Low-Class	Illiterate	She had 2 children, and her most recent childbirth occurred within six months before the interview date.
	N6	20	Low-Class	Illiterate	She had no prior pregnancy experience was in the third trimester of her pregnancy during the interview.
	N7	21	Low-Class	Illiterate	She had 1 child and was in the second trimester of her pregnancy during the interview.
	N8	23	Low-Class	Illiterate	She had 2 child and was in the second trimester of her pregnancy during the interview.
	N9	20	Low-Class	Illiterate	She had 1 child, and her most recent childbirth occurred within seven months before the interview date.
	N10	21	Low-Class	Illiterate	Had no previous experience of pregnancy and her most recent childbirth occurred within three months before the interview date.
	N11	20	Middle-Class	She finished primary school education	She had 1 child, and her most recent childbirth occurred within six months before the interview date.
	N12	24	High-Class	She finished primary school education	She had 1 child and was in the third trimester of her pregnancy during the interview.

Figure 1 presents ten themes identified through open-coding, categorized into three main groups and analyzed using an intersectional approach. These three categories are: ‘institutional racism,’ ‘personally mediated racism,’ and ‘women's agency.’

The first two categories correspond to Jones’s institutionalized and personally mediated racism levels. When discussing about their experience of institutional and personally mediated

racism in this study, the Garo women recurrently pointed out the issue of exploitation. Their interactions with hospital staff consistently revealed instances of exploitation, a shared concern within the group. While experiences were consistent among participants, the author recognizes individual nuances, emphasizing the need to address and improve the overall maternity care experiences of Garo Indigenous women.

The third category, 'women's agency,' emerged as a response to the first two categories. This category is unique in this study. Jones's theory suggests that institutional and personally mediated racism leads to the internalization of racism (22,45). On the contrary, in this study evidence demonstrates that these two levels of racism strengthen Garo Indigenous women's agency of cultural resistance practices against the encountered institutional and personally mediated racism.

Figure 1: Institutional racism, personally mediated racism, and women agency in maternal healthcare

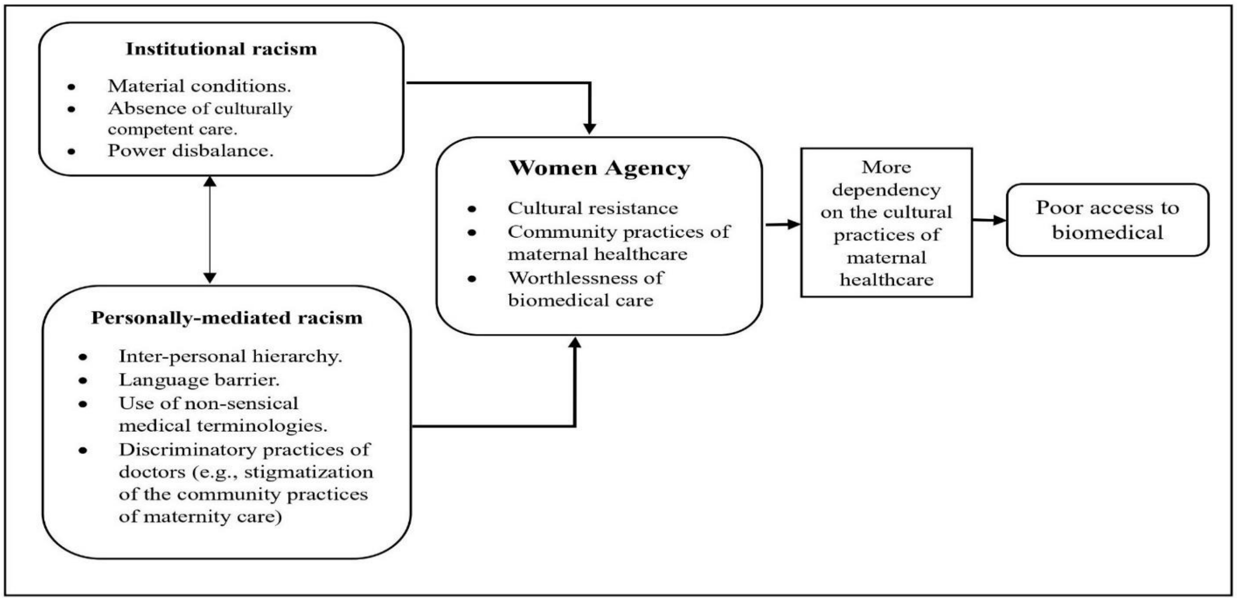

Institutional racism

Institutional racism is evident in material conditions, culturally inappropriate care, and resulting power imbalances. Regardless of economic class and education level, participants faced challenges accessing maternal healthcare due to clinicians' claims of insufficient resources and power imbalances. For example, one participant reported clinicians' using the excuse of unavailability of medical facilities and limited supplies for discriminatory care at the hospital. As she typically discussed:

> During my last pregnancy, I saw a big difference in how doctors treated non-Garo women compared to me at the hospital. They got good care with all they needed, but the doctor said they didn't have enough for me. I was surprised when a non-Garo woman got proper care for the same problem. In our community, it's hard to access good medical facilities, so getting the care we need during pregnancy is tough. Doctors often use shortages of machines and staffs as excuses, making things even harder for us (N4).

This Participant described discrimination in care compared to non-Garo women at the hospital, suggesting healthcare service disparities based on Indigenous identity. Non-Garo women received proper care, while the participants faced excuses about insufficient resources, raising concerns about institutional racism. Reflecting similar views with other participants, another participant felt ignored by clinicians when seeking biomedical care due to their focus on non-Garo patients. As she reported: "due to the gynecologists' preference for non-Garo women, pregnancy care from hospital becomes impossible for us. But we don't expect this (L8)". When asked if experiencing discrimination at the hospital prevented Garo Indigenous women from seeking maternal healthcare, another woman typically responded: "Yes... Honestly, I can't recall a time

when I went to a doctor’s office for maternal care after facing discrimination during my first visit (L3)” As this Participant highlights, lacking access to equitable healthcare treatment often translates to limited access to medical professionals. Consequently, the women interviewed commonly pointed out inadequate services, discriminatory treatment, and clinicians' preference for local women.

Furthermore, all the participants consistently voiced concerns regarding the hospital’s lack of culturally appropriate care. This was particularly troubling for them as receiving biomedical care conflicting with their community's maternal healthcare practices was not acceptable. As one of them typically shared:

> I felt deeply troubled by the lack of alignment between the available healthcare at the hospital and our community’s pregnancy healthcare practices. Following our cultural traditions is essential to us, and it was challenging for me to comprehend and accept healthcare practices that didn't respect our customs. It left me feeling confused and uneasy. (L6).

This participant’s troubled, confused, and uneasy feelings indicate a dissonance between their expectations and the care provided. Another participant also mentioned that culturally inappropriate care includes not being able to meet the birth attendant before delivering in a hospital, which goes against their cultural norms and expectations:

> I don’t understand why the doctors won’t let us meet the midwife before giving birth. It’s essential for us, and they don't seem to care about our cultural needs. They mistreat us, saying they can’t do anything because of the hospital's rules (N6).

In addition, another woman with a common experience similar to other participants expressed about the birthing dress code: “we can’t go inside the delivery room without wearing the hospital's

dress, given by the doctors. It’s unfair and against our norms related to birth delivery because many women already wear their clothes during delivery (L1)”.

While discussing the reasons behind the absence of culturally appropriate care, the participants generally pointed out issues such as clinicians’ inadequate cultural training, lack of community engagement, and limited knowledge about traditional healing practices. As one of the participants generally discussed:

> I think the doctors don't understand our ways and beliefs. They don't involve our community in decisions. Our traditions are ignored, making us feel disconnected. They don't know much about our healing practices, so they prefer their own ways (L12).

This participant discussed her frustration and dissatisfaction with the healthcare system’s lack of cultural understanding and involvement of their community in decision-making. She also expresses disconnection and alienation due to disregarding their traditions and healing practices. Interestingly, she and other low-income women also stressed the importance of female clinicians. In contrast, many other participants linked the absence of Garo female doctors to culturally inappropriate care, highlighting the gender sensitivity of maternal health issues:

> I am deeply concerned about absence Garo female doctors. The non-Garo doctors does not have any care and respect for us and seeking care from non-Garo male doctor is against out our culture because we can’t share our health problem to a male doctor (L5).

In addition, unlike the participants from the high-class household income group, the Garo women from the middle-class and low-class household income statuses highlight the hidden costs of seeking medical treatment, such as transportation expenses and expensive hospital food. The disparity between the claimed cost and hospital charges points to potential financial exploitation.

The lack of control over travel and treatment costs further adds to the burden on these women. The participant's frustration with the hospital's dismissive attitude toward their concerns reflects a systemic issue of healthcare institutions not adequately addressing the needs and challenges faced by Indigenous communities. As one of the participants shared:

> Distance cost isn't all. Hospitals want money. Despite their claim, they don't charge 5 Taka (Bangladeshi currency). They demand more. Traveling makes you hungry, but food is expensive. We must spend all we earn if we go. We don't control travel and treatment costs. They ignore our issue. They said 'you have to follow the hospital rules' whenever we told them our problem. What else can we do but avoid it? (L2).

This participant's discussion highlights Indigenous women's financial obstacles when seeking healthcare, including hidden costs and potential hospital exploitation. It serves as an example of financial cost-driven discrimination in biomedical maternity care, highlighting how institutionalized ethnocentrism impacts access to healthcare for Garo women.

Overall, the participants typically blamed insufficient human resources, culturally inappropriate care, and the dominant power of the clinicians for poor access. Also, they present the obligation of following the institutional guidelines defined by the clinicians as a reason for not accessing these services. This illustrates a link to personally-mediated racism, as presented in the following sub-section.

Personally-mediated racism

Garo participants faced personally-mediated racism from clinicians, evident in the inter-personal hierarchy, use of non-sensical medical terms, and discriminatory practices like stigmatizing community maternity care practices. Language-related racism was also present, with clinicians

viewing Garo participants' use of the Mandi language as problematic, leading to miscommunication and discrimination against indigenous language speakers for not using mainstream language:

> They (the clinicians) don't understand what we're saying. We don't want to be treated by him because he's aggressive and doesn't know how. Our Garo women went there with health issues like jak jachak dalya (swelling of feet and hands), wakkalna ha'sika (vomiting), matha betha (dazziness), Hapani (shortness of breath), and bikma chikki sadika (abdominal pain), but the doctor didn't listen. They give us medicine even though they don't understand (N5).

This participant shared troubling experiences with clinicians lacking cultural understanding and empathy, expressing frustration with their aggressive and dismissive behavior, leaving her feeling unheard and misunderstood. Other participants reported similar experiences, even those from high-class households with primary education. For instance, bilingual Garo women faced discrimination at the hospital due to difficulty pronouncing Bengali words. Using Bengali in the hospital was emotionally and practically harder than in daily life, and clinicians' unfamiliar medical terminology added to their challenges. As one of the participants shared:

> Though I can't pronounce Bengali words well, I can explain my health issues to doctors. But, I'm hesitant to go again because the doctors laughed at me when I talked about my health issues a few months ago. The doctors replied with medical jargon. He laughed again when I asked him to explain these. I felt insulted (L7).

As this participant discussed, she encounters challenges as an Indigenous woman seeking healthcare. Despite her efforts to communicate in Bengali, the clinicians' dismissive attitude and use of medical jargon created a barrier to understanding. Their laughter made her feel demeaned

and disrespected. This reflects a broader cultural issue where language barriers and lack of empathy hinder effective communication and care. Additionally, participants highlighted the issue of power and domination over the Garo women's bodies. They mentioned clinicians using force, touching private organs without permission, and not allowing the family to perform cultural rituals with the placenta, which were significant barriers to respectful maternal healthcare. One mother typically reported: "Our culture forbids delivering babies by cutting the belly. They touch our private organ during mid-pregnancy visits. Permission was never sought. They forcefully violate our culture (N2)."

The participant's quote indicates that clinicians exerted power to ignore the Garo women's cultural norms for maternity care and pressured them to adopt biomedical care as a symbol of modernity. The women's accounts also revealed that clinicians' perception of community maternal healthcare practices stigmatized them, creating obstacles in accessing the hospital:

> The doctors don't listen to our cultural needs, instead blaming us for our customs, such as not cutting the belly and reciting the delivery mantra. They said we are responsible for our health complications, especially birthing complications (N5).

Participants generally shared experiences of clinicians' discriminatory practices, stigmatizing them for adhering to community maternity healthcare. Many feared hospital visits due to concerns of forced C-sections or vaginal incisions, violating cultural norms and reproductive health. Clinicians' ethnocentrism blamed severe maternal health complications on community practices, disregarding Indigenous culture and pushing for biomedical control. Thus, the doctor-Garo mother hierarchy is revealed:

> Whenever to visit the doctor, they stigmatize us for our belief in our community norms related to pregnancy health care. Disrespecting our community practices of not cutting the belly for delivery, they forced us to cut the belly. In fact, the doctor decides how to deliver, not we (N9).

The data shows that clinicians' preference for C-sections for Garo women reflects institutionalized racism, disregarding the cultural belief in the safety of normal birthing. This interplay between institutional and personally-mediated racism shapes internalized racism among the Garo Indigenous women, as discussed in the following sub-section.

Women Agency

The sub-themes that emerged this category include, cultural resistance, community practices of maternal healthcare, and perceived worthlessness of biomedical care.

Concerning agency, while discussing the effect of the previous experience of being stigmatized, a mother typically reported her agency in deciding to avoid seeking biomedical care:

> Now, I do things my way when it comes to taking care of myself during pregnancy. I don't go to the hospital because they don't understand us. They make me feel small and don't listen to what I say. (N5).

This participant's discussion reflects empowerment through embracing traditional practices rooted in her cultural heritage. Exercising agency means she actively made choices based on her cultural beliefs, not just accepting the mainstream healthcare approach. She shows resilience in navigating a healthcare system that often fails to understand her unique needs. Her decision to avoid biomedical care is a response to the stigma and lack of respect she experienced in that setting. When asked, in addition to clinicians' stigmatization and discrimination, what else helps facilitate

such agency to decide to avoid seeking biomedical care, she replied about her community bonding and support as another source for that:

> No, it’s not just because of doctors’ blaming and discrimination. Also, because our community women who are supportive to each other. Our elders know so much, and I trust them. All these gave me courage to decide to avoid going hospital (N5).

Interestingly, the interviewed women linked their perceived agency to avoid going hospital with the worthlessness of biomedical care compared to community practices of maternity care. Institutionalized racism and clinicians’ personally-mediated racism, shown in power hierarchies and stigmatization, influenced their decision to rely more on community practices. One participant shared:

> Hospital-level care is value-less to me because they disrespect us. When I need help with my health, I go to our traditional healers. They know our beliefs and take care of us with respect. I want to keep our traditions alive and stay connected to who we are (L7).

This participant’s experience of institutionalized and personally-mediated racism shows how it becomes a source of culturally empowered agency to internalize the comparative benefits of community practices in maternal healthcare. For this participant, being sure to be culturally humiliated at the hospital was something she saw as a sign of strength to decide on an alternative source of healthcare. When asked what would have happened if she had not been able to get cured of possible maternal health complications, she responded:

> I knew from my neighbor women and mother, who had their previous pregnancy experiences that if I follow our cultural practices, then there will be no pregnancy health illness. So, why should I visit hospital to be humiliated and encounter discrimination? (L7).

When asked about the types of their community practices of maternal healthcare, along with this participant, another woman typically shared:

> We have several norms for a safe pregnancy, including avoiding houses where someone has died, not using mouth to fan fire smoke while cooking, eating locally sourced and culturally specific foods, spending time outdoors, participating in traditional activities, and seeking solace in natural environments. We also follow rituals and ceremonies related to pregnancy and rely on medicinal herbs and plants for managing pregnancy discomforts, promoting healthy labor, and aiding postpartum recovery. Additionally, we seek care from traditional healers and birth attendants within our own community (L8).

This participant reported the importance and her dependency on the community practices of maternal healthcare as the manifestation of her resistance practices against the clinicians' discriminatory practices as a means to offset the clinician's intention of stigmatizing her. Similar to other respondents, another participant felt that as she encountered discrimination at the hospital, she would not have been able to resist such racism against her without increasing her dependency on traditional healers whom she found far better:

> I was able to avoid getting stigmatized by the doctors when I started following our community norms of pregnancy healthcare, I had good health care without making further visit to the hospital. ….I went to traditional healer to all my prenatal visits…I had it good (N11).

Thus, the participants' experiences indicate that the internalization of the intersectional mix of institutional and personally-mediated racism by clinicians led to feelings of disempowerment and self-devaluation at hospitals. They perceived biomedical care as less valuable than community practices of maternal healthcare, which they found non-discriminatory. As a result, they sought

cultural empowerment and resistance through community-based maternity care to avoid discrimination and stigmatization at hospitals.

Discussions

This qualitative descriptive study investigates the impact of racism on Garo Indigenous women's maternal healthcare experiences, employing Jones's 'Three Levels of Racism' theory (22) and an intersectionality approach (19). Nevertheless, the study's results, as outlined in the previous section, reveal a significant deviation from Jones's theory regarding the three levels of racism within the specific context of Garo Indigenous women's experiences. Jones's theory usually focuses on how racism is internalized due to institutional and personally-mediated factors. On the contrary, this study uncovers a unique theme: 'women agency.' This theme emerges as a robust response to institutional and personally-mediated racism within the Indigenous women studied, highlighting their cultural resistance and resilience. The findings suggest that the complex relationship between these two forms of racism contributes to the strengthening of agency among Garo Indigenous women. This, in turn, shapes their preference for community-based maternal healthcare, which differs from a previous study (45).

Additionally, the current study uncovers the persistent and multifaceted discrimination faced by Garo Indigenous women in accessing maternity care, driven by institutional racism (22). Participants generally highlight issues like lacking culturally competent care and power imbalances due to Indigenous identity-based racism at the institutional level. The exclusionary practices in healthcare, justified by resource constraints, reinforce institutional racism. This finding adds to a prior study that examined how Indigenous identity-based racial discrimination is ignored by institutions providing biomedical care services (33). However, it contrasts with prior research

in Ethiopia, where actual unavailability reduced Indigenous maternal healthcare access, indicating context-specific variations (41,42).

The author's use of intersectionality (19) within Jones's framework of institutional racism (22) to analyze participant accounts suggests the connected and overlapping aspects of discrimination driven by these dimensions of institutional racism that specifically affect participants' access to biomedical healthcare. Intersectionality demonstrates how these women's social background, Indigenous identity, and gender interact (19). Race and socioeconomic position interact in the Upazila hospital's care and resource distribution, as the participants highlighted. Gender and Indigenous identity intersect to discriminate against these women in healthcare as seen by the lack of Garo female doctors and the preference for non-Garo male doctors. Hidden costs and financial exploitation are experienced by Garo women from economically underprivileged communities, demonstrating racial and financial bias. As the participants discussed, institutional racism results from the clinicians' dominating posture in forcing them to follow hospital policies. This position shows a power imbalance between men and women and is based on race. These findings are well supported by previous studies highlighting the intersectionality among race, gender, and socio-economic status of Indigenous women (17–19).

The participants' stories make clear the ingrained stereotypes and discriminatory practices that Garo Indigenous women must deal with within the healthcare system. Jones's theses of personally-mediated racism (22) which takes into account inter-personal hierarchy and asymmetric power relations based on 'othering' (56), fits issues like inter-personal hierarchy, language obstacles, and discriminatory practices of clinicians as encounters by the Garo women. The power relations between biomedical institutions and Indigenous women regarding maternity care have been the subject of prior studies conducted in several nations (27,31,43). In this study, also, this

tendency is also evident. As a result of Bangladesh's health policy, which perpetuates colonial stereotypes by classifying Indigenous people as a separate tribe, clinicians are unable to fully comprehend the imbalances in power that exist with Garo women (58,59), It prevents clinicians from understanding historical and contemporary contexts and identifying unequal power relations with Garo women. Thus, participants' discussion suggests that clinicians following service guidelines reproduce colonial prejudices and stereotypes in an institutionalized context. It means the levels of racism against Indigenous Garo women often reinforce each other.

Accordingly, an intersectional analysis of the participants' discussion shows that their experiences, similar to institutional racism, are influenced not only by their Indigenous identity but also by their gender, culture, and socioeconomic level (19). The difficulties and prejudices people confront in the healthcare system are exacerbated by these interconnected identity characteristics. The participants' discussions shed light on how personally-mediated racism intertwines with an inter-personal power hierarchy, resulting in ongoing discrimination. Nonsensical medical jargon and discriminatory attitudes toward the Garo language exacerbate their marginalization.

Furthermore, the clinicians' racist approach and lack of consideration for cultural norms during pregnancy and childbirth underscore the impact of race and culture on healthcare access. Other studies have shown how segregationist colonialism and assimilationist policies have influenced relations between public institutions and Indigenous communities (32,44), creating a racialized cultural logic of intercultural connections (31). This study adds to these findings, demonstrating that racism intersects with other forms of discrimination (19). For instance, the dominance of Western medical perspectives and stigmatization of Garo women for following their community's maternity healthcare practices highlight the intersection of race, cultural norms, and

colonial legacies, leading to gendered power hierarchies and clinicians' attitudes toward Garo women. Moreover, the fear of forced medical procedures and clinicians' decisions further emphasize the intersectionality of race, gender, and power hierarchies, perpetuating vulnerabilities among Indigenous women within the healthcare system. These findings contribute to previous research (28), deepening the understanding of discrimination faced by Indigenous women.

The participants' discussion on 'agency' reveals its profound impact on their healthcare. Themes like cultural resistance, community practices, and perceived worthlessness of biomedical care show how it shapes their decisions and cultural preservation. They resist biomedical care due to stigmatization, embracing community-based practices to strengthen their cultural identity. This contrasts with a study from Mexico where women abandoned Indigenous practices for biomedical care (31). The intersectional analysis highlights that the participants' agency is not solely derived due to individual attitudes but also institutional practices, policies, and personally-mediated racism.

To sum up, this study uncovers the multifaceted impact of racism on Garo Indigenous women's healthcare access. Through the lens of intersectionality, it becomes evident that race, gender, culture, and socioeconomic status intersect to compound the barriers they face. This intersectional perspective deepens our understanding of institutional and personally-mediated racism, shedding light on how it shapes the agency of these women. The study contributes to the previous research on racism and healthcare (3,31,45,68), emphasizing the need to address both individual prejudices and systemic inequities in healthcare systems.

The study shows based on the evidence of the reports of Garo women: there is strong racism at different levels, and it strongly compromises biomedical healthcare access. Garo women

count on a strong traditional medicine system to attend maternal healthcare. Garo women agency derives in their decision to avoid hospitals were they and their culture is mis-respected. Therefore, to increase biomedical healthcare access for Garo women racism needs to be present in the institutional and relational level.

Therefore, the current study suggests for implementing intercultural competency training program (CICTP) for the clinicians. This program should incorporate 'cultural humility' approaches, which recognize and respect intersecting identities of race, gender, cultural identity, and socioeconomic status (23). This approach aims to address power imbalances at personal and structural levels of racism (23). Implementing programs designed with cultural humility principles (23) can create an inclusive and empowering environment for Garo Indigenous women, acknowledging their agency in healthcare decisions. Furthermore, it would help initiate constructive dialogues with between the clinicians and the Garo Indigenous women to address the latter group's shared experience of exploitation.

Conclusion

Racism is a vital maternity health concern for Garo Indigenous women, with institutional racism and personally-mediated racism manifesting in discrimination and stigmatization due to language and community cultural norms related to maternal health care. This study presents the multi-dimensional nature of racism, as typically discussed by the participants, and how it impacts their maternal healthcare access. The findings emphasize the significance of intersectionality, showcasing how institutionalized racism and personally-mediated racism generates Garo Indigenous women's agency and thus, shape the experiences of maternal healthcare. Furthermore, it underscores the importance of recognizing and respecting intersecting identities of race, gender,

cultural identity, and socioeconomic status to address power disbalances at both personal and structural levels of racism. It highlights the need for clinicians' intercultural competency training programs incorporating 'cultural humility' approaches to create an inclusive and empowering healthcare environment for Garo Indigenous women that ensure equity in healthcare access.

References

1. Shiraev EB, Levy DA. Cross-cultural psychology: Critical thinking and contemporary applications. Routledge; 2020.

2. Creanga AA, Bateman BT, Kuklina E V, Callaghan WM. Racial and ethnic disparities in severe maternal morbidity: a multistate analysis, 2008-2010. Am J Obstet Gynecol. 2014;210(5):435-e1.

3. Nuru-Jeter A, Dominguez TP, Hammond WP, Leu J, Skaff M, Egerter S, et al. "It's the skin you're in": African-American women talk about their experiences of racism. An exploratory study to develop measures of racism for birth outcome studies. Matern Child Health J. 2009;13:29–39.

4. Petersen EE, Davis NL, Goodman D, Cox S, Syverson C, Seed K, et al. Racial/ethnic disparities in pregnancy-related deaths—United States, 2007–2016. Morbidity and Mortality Weekly Report. 2019;68(35):762.

5. Mateo CM, Williams DR. Racism: a fundamental driver of racial disparities in health-care quality. Nat Rev Dis Primers. 2021;7(1):20.

6. Feagin J, Bennefield Z. Systemic racism and US health care. Soc Sci Med. 2014;103:7–14.

7. Davis DA. Reproductive injustice: Racism, pregnancy, and premature birth. Oxford University Press; 2020.

8. Jordal M, Wahlberg A. Challenges in providing quality care for women with female genital cutting in Sweden–A literature review. Sexual & reproductive healthcare. 2018;17:91–6.

9. Poetry S. Giving Birth in South Florida : A Phenomenological Study of the Pregnancy and Birthing Experiences among Black and Afro-Latina Women. 2020.

10. Petersen EE, Davis NL, Goodman D, Cox S, Syverson C, Seed K, et al. Racial/ethnic disparities in pregnancy-related deaths—United States, 2007–2016. Morbidity and Mortality Weekly Report. 2019;68(35):762.

11. Dossetor PJ, Freeman JM, Thorburn K, Oscar J, Carter M, Jeffery HE, et al. Health services for aboriginal and Torres Strait Islander children in remote Australia: A scoping review. PLOS Global Public Health. 2023;3(2):e0001140.

12. Thapa R, Teijlingen E Van, Regmi PR, Heaslip V. Caste Exclusion and Health Discrimination in South Asia : A Systematic Review. 2021;

13. Kozhimannil KB, Interrante JD, Tofte AN, Admon LK. Severe maternal morbidity and mortality among indigenous women in the United States. Obstetrics and gynecology. 2020;135(2):294.

14. MacDonald C, Steenbeek A. The impact of colonization and western assimilation on health and wellbeing of Canadian aboriginal people. International Journal of Regional and Local History. 2015;10(1):32–46.

15. Adhikari T, Sahu D, Nair S, Saha KB, Sharma RK, Pandey A. Factors associated with utilization of antenatal care services among tribal women: A study of selected States. Indian J Med Res. 2016;144(1):58.

16. Küey L. Clarifying Definitions of „Race ", Racism, and Ethnocentrism. European Psychiatry. 2022;65(S1):S35–S35.

17. Varcoe C, Brown H, Calam B, Harvey T, Tallio M. Help bring back the celebration of life: A community-based participatory study of rural Aboriginal women's maternity experiences and outcomes. BMC Pregnancy Childbirth. 2013;13(1):1–10.

18. Anne Van Herk K, Smith D, Andrew C. Identity matters: Aboriginal mothers' experiences of accessing health care. Contemp Nurse. 2011;37(1):57–68.

19. Crenshaw K. Demarginalizing the intersection of race and sex: A black feminist critique of antidiscrimination doctrine, feminist theory and antiracist politics. u Chi Legal f. 1989;139.

20. Figueroa JF, Frakt AB, Jha AK. Addressing social determinants of health: time for a polysocial risk score. JAMA. 2020;323(16):1553–4.

21. Paradies Y. A systematic review of empirical research on self-reported racism and health. Int J Epidemiol. 2006;35(4):888–901.

22. Jones CP. Levels of racism: a theoretic framework and a gardener's tale. Am J Public Health. 2000;90(8):1212.

23. Tervalon M, Murray-Garcia J. Cultural humility versus cultural competence: A critical distinction in defining physician training outcomes in multicultural education. J Health Care Poor Underserved. 1998;9(2):117–25.

24. Reddy S, Patel N, Saxon M, Amin N, Biviji R. Innovations in US health care delivery to reduce disparities in maternal mortality among African American and American Indian/Alaskan Native women. J Patient Cent Res Rev. 2021;8(2):140.

25. Allen L, Wodtke L, Hayward A, Read C, Cyr M, Cidro J. Pregnant and early parenting Indigenous women who use substances in Canada: A scoping review of health and social issues, supports, and strategies. J Ethn Subst Abuse. 2022;1–31.

26. Lawford KM, Giles AR, Bourgeault IL. Canada's evacuation policy for pregnant First Nations women: resignation, resilience, and resistance. Women and Birth. 2018;31(6):479–88.

27. Hayward A, Cidro J. Indigenous birth as ceremony and a human right. Health Hum Rights. 2021;23(1):213.

28. Akinade T, Kheyfets A, Piverger N, Layne TM, Howell EA, Janevic T. The influence of racial-ethnic discrimination on women's health care outcomes: A mixed methods systematic review. Soc Sci Med. 2023;316:114983.

29. Zestcott CA, Blair I V, Stone J. Examining the presence, consequences, and reduction of implicit bias in health care: a narrative review. Group Processes & Intergroup Relations. 2016;19(4):528–42.

30. Hassen N, Lofters A, Michael S, Mall A, Pinto AD, Rackal J. Implementing anti-racism interventions in healthcare settings: a scoping review. Int J Environ Res Public Health. 2021;18(6):2993.

31. Dörr NM. Health professionals' perception of maternal health: an ethnography in the Sierra de Totonacapan, Veracruz, Mexico. Social Medicine. 2020;13(3):155–65.

32. Butler R, Ben J. Centring settler colonialism in rural Australian multicultures: race, place and local identities. J Ethn Migr Stud. 2021;47(9):2179–97.

33. Rice ZS, Liamputtong P. Culture and Global Health: Cultural Determinants of Health and Well-Being. Handbook of Global Health. 2020;1–26.

34. Akter S. Examining access to health services for women of reproductive age from ethnic communities in Chittagong Hill Tracts (CHT) of Bangladesh.

35. Thresia CU, Srinivas PN, Mohindra KS. The Health of Indigenous Populations in South Asia : A Critical Review in a Critical Time. 2022;

36. Akter S, Davies K, Rich JL, Inder KJ. Barriers to accessing maternal health care services in the Chittagong Hill Tracts, Bangladesh: A qualitative descriptive study of Indigenous women's experiences. PLoS One. 2020;15(8):e0237002.

37. Chowdhury SK. Uptake of antepartum care services in a matrilineal-matrilocal society: a study of Garo indigenous women in Bangladesh. BMC Pregnancy Childbirth. 2023;23(1):75.

38. Akter S, Davies K, Rich JL, Inder KJ. Community perspectives of barriers indigenous women face in accessing maternal health care services in the Chittagong Hill Tracts, Bangladesh. Ethn Health. 2022;27(5):1222–40.

39. Dehon E, Weiss N, Jones J, Faulconer W, Hinton E, Sterling S. A systematic review of the impact of physician implicit racial bias on clinical decision making. Academic Emergency Medicine. 2017;24(8):895–904.

40. FitzGerald C, Hurst S. Implicit bias in healthcare professionals: a systematic review. BMC Med Ethics. 2017;18(1):1–18.

41. Khosla R, Zampas C, Vogel JP, Bohren MA, Roseman M, Erdman JN. International human rights and the mistreatment of women during childbirth. Health Hum Rights. 2016;18(2):131.

42. Ibrahima AB. Exploring Maternal Health in Ethiopia Using Indigenous Approaches: Policy and Practice Implications. Res Soc Work Pract. 2021;31(4):343–50.

43. Yeung S. Conceptualizing cultural safety: Definitions and applications of safety in health care for Indigenous mothers in Canada. Journal for Social Thought. 2016;1.

44. Moran A. White Australia, settler nationalism and Aboriginal assimilation. Australian Journal of Politics and History. 2005;51(2):168–93.

45. Janevic T, Sripad P, Bradley E, Dimitrievska V. " There's no kind of respect here" A qualitative study of racism and access to maternal health care among Romani women in the Balkans. Int J Equity Health. 2011;10(1):1–12.

46. Khosla R, Zampas C, Vogel JP, Bohren MA, Roseman M, Erdman JN. International human rights and the mistreatment of women during childbirth. Health Hum Rights. 2016;18(2):131.

47. Tamanna S, Rana MM, Ferdoushi A, Ahmad SAI, Rahman M, Rahman A. Assessment of nutritional status among adolescent Garo in Sherpur District, Bangladesh. Bangladesh Journal of Medical Science. 2013;12(3):269–75.

48. Hasan M, Momtaz P, Hosen I, Das SA, Akhteruzzaman S. Population genetics of 17 Y-chromosomal STRs loci in Garo and Santal tribal populations in Bangladesh. Int J Legal Med. 2015;129(2):251–2.

49. Dey S, Resurreccion BP, Doneys P. Gender and environmental struggles: voices from Adivasi Garo community in Bangladesh. Gender, Place & Culture. 2014;21(8):945–62.

50. Das TK, Islam S. Psycho-social dimensions of ethnicity: The situation of Garo community in Bangladesh. Asian Aff (Lond). 2005;27(3):45–54.

51. Das DrTK. Psycho-Social dimensions of ethnicity : The situation of the Garo community in Bangladesh. Journal of Ethnic Affairs [Internet]. 2005;I(1):5–10. Available from: www.ecdo-bd.org

52. Islam MR, Islam MA, Banowary B. Determinants of exposure to mass media family planning messages among indigenous people in Bangladesh: A study on the Garo. J Biosoc Sci. 2009;41(2):221–9.

53. Rahman A. Fighting Exclusion: Towards Understanding the Predicament of Adivasis in Bangladesh. In: Political Governance and Minority Rights. Routledge India; 2020. p. 104–20.

54. Bal E, Chambugong TC. The Borders that Divide, the Borders that Unite: (Re)interpreting Garo Processes of Identification in India and Bangladesh. J Borderl Stud. 2014;29(1):95–109.

55. Bal E. Becoming the Garos of Bangladesh: Policies of exclusion and the ethnicisation of a 'tribal'minority. South Asia: Journal of South Asian Studies. 2007;30(3):439–55.

56. Spivak G. In Other Worlds: Essays in Cultural Politics. New York: Methuen. other worlds: essays in cultural politics. 1987;

57. Le Roux J, Oyedemi TD. Indelible apartheid: intergenerational post-colonial narratives of colonial-born coloured females about hair, race and identity in South Africa. Social Identities. 2022;28(2):150–65.

58. Gerharz E. Indigenous activism in Bangladesh: Translocal spaces and shifting constellations of belonging. Asian Ethnicity. 2014;15(4):552–70.

59. Akter S. Factors influencing health service utilization among mothers for under-five children: A cross-sectional study in Khulna district of Bangladesh. PLoS One. 2022;17(9):e0274449.

60. Navile TE, Hossain J. BIRTH RITUAL, PRACTICES AND GENDER PREFERENCE FOR EXPECTING CHILD IN A MATRILINEAL INDIGENOUS COMMUNITY: A CASE OF GAROS LIVING IN SAINAMARI VILLAGE, MADHUPUR, BANGLADESH. Journal of the Asiatic Society of Bangladesh (Hum). 2019;64(2):239–50.

61. Bhowmik KR, Das S, Islam MA. Modelling the number of antenatal care visits in Bangladesh to determine the risk factors for reduced antenatal care attendance. PLoS One. 2020;15(1):e0228215.

62. Ame AS, Mozumdar L, Islam MA. Impact of social networks on the choice of place of delivery among ethnic women in Bangladesh. Sexual & Reproductive Healthcare. 2021;28:100588.

63. Akter S. Examining access to health services for women of reproductive age from ethnic communities in Chittagong Hill Tracts (CHT) of Bangladesh.

64. Massey DS. Segregation and stratification: A biosocial perspective. Du Bois Rev. 2004;1(1):7–25.

65. Omi M, Winant H. Racial formation rules. Racial formation in the twenty-first century. 2012;302.

66. Bonilla-Silva E. Racism without racists: Color-blind racism and the persistence of racial inequality in the United States. Rowman & Littlefield Publishers; 2006.

67. Jones CP, Truman BI, Elam-Evans LD, Jones CA, Jones CY, Jiles R, et al. Using "socially assigned race" to probe white advantages in health status. Ethn Dis. 2008;18(4):496–504.

68. Janevic T, Piverger N, Afzal O, Howell EA. “Just because you have ears doesn’t mean you can hear”—perception of racial-ethnic discrimination during childbirth. Ethn Dis. 2020;30(4):533.

69. Calabrese SK, Meyer IH, Overstreet NM, Haile R, Hansen NB. Exploring discrimination and mental health disparities faced by Black sexual minority women using a minority stress framework. Psychol Women Q. 2015;39(3):287–304.

70. Batist J. An intersectional analysis of maternal mortality in Sub-Saharan Africa: a human rights issue. J Glob Health. 2019;9(1).

71. Hoang TMH, Wong A. Exploring the Application of Intersectionality as a Path toward Equity in Perinatal Health: A Scoping Review. Int J Environ Res Public Health. 2022;20(1):685.

72. Grollman EA. JOURNAL of HEALTH and SOCIAL BEHAVIOR. J Health Soc Behav. 2014;55(1).

73. Karim MA, Kabir MM, Siddiqui MA, Laskar MSI, Saha A, Naher S. Epidemiology of Imported Malaria in Netrokona District of Bangladesh 2013-2018: Analysis of Surveillance Data. Malar Res Treat. 2019;2019.

74. Rahman SMB, Uddin MB, Hussain I. Anthropometric study on children of Garo and non-Garo families in Netrakona district of Bangladesh. Journal of the Bangladesh Agricultural University. 2011;9(2):267–72.

75. Rahman M. Paradoxes of participation of the women leaders in the union parishad in Bangladesh: A phenomenological analysis. Jurnalul Practicilor Comunitare Pozitive. 2019;19(2):58–73.

76. Liamputtong P. Performing qualitative cross-cultural research. Cambridge University Press; 2010.

77. Sandelowski M. Whatever happened to qualitative description? Res Nurs Health. 2000;23(4):334–40.

78. Sandelowski M. Whatever happened to qualitative description? Res Nurs Health. 2000;23(4):334–40.

79. Mayan MJ. Essentials of qualitative inquiry. Routledge; 2016.

80. Sandelowski M. Whatever happened to qualitative description? Res Nurs Health. 2000;23(4):334–40.

81. Caelli K, Ray L, Mill J. ‘Clear as mud’: toward greater clarity in generic qualitative research. Int J Qual Methods. 2003;2(2):1–13.

82. Akter S, Davies K, Rich JL, Inder KJ. Barriers to accessing maternal health care services in the Chittagong Hill Tracts, Bangladesh: A qualitative descriptive study of Indigenous women’s experiences. PLoS One. 2020;15(8):e0237002.

83. Merz S, Jaehn P, Pischon T, Fischer B, Wirkner K, Rach S. Investigating people ' s attitudes towards participating in longitudinal health research : an intersectionality - informed perspective. 2023;1–18.

84. Klar S, Leeper TJ. Identities and intersectionality: a case for Purposive sampling in Survey-Experimental research. Experimental methods in survey research: Techniques that combine random sampling with random assignment. 2019;419–33.

85. Etikan I, Alkassim R, Abubakar S. Comparision of snowball sampling and sequential sampling technique. Biom Biostat Int J. 2016;3(1):55.

86. Atkinson R, Flint J. Accessing hidden and hard-to-reach populations: Snowball research strategies. Social research update. 2001;33(1):1–4.

87. Cochran PAL, Marshall CA, Garcia-Downing C, Kendall E, Cook D, McCubbin L, et al. Indigenous ways of knowing: Implications for participatory research and community. Am J Public Health. 2008;98(1):22–7.

88. Milne J, Oberle K. Enhancing rigor in qualitative description. Journal of Wound Ostomy & Continence Nursing. 2005;32(6):413–20.

89. Braun V, Clarke V. Using thematic analysis in psychology. Qual Res Psychol. 2006;3(2):77–101.

90. Chowdhury SK. Uptake of antepartum care services in a matrilineal-matrilocal society: a study of Garo indigenous women in Bangladesh. BMC Pregnancy Childbirth. 2023;23(1):75.

Click here to access/download
Supporting Information
Ethics Approval Letter.pdf

Click here to access/download
Other
Data Set.pdf

Racism and Access to Maternal Health care Among Garo Indigenous Women in Bangladesh: A Qualitative Descriptive Study

Author's contributions

The author's interest in Indigenous women's maternal health led to this research. He reviewed relevant literature, designed research instruments, and served as the principal researcher, writing both the initial and final manuscript drafts.

Acknowledgement

The author would like to express gratitude to the anonymous reviewers for their valuable input. This article is based on the author's MSc thesis work, which was supervised by Dr. Joyee S. Chatterjee, Program Chair of Gender and Development Studies at AIT. The author extends heartfelt thanks to Dr. Chatterjee. Special recognition is given to Shila Gagra for her assistance in developing culturally relevant interview questions and recruiting four female research assistants: Manisha, Namita, Malita, and Tomita. The author sincerely appreciates their active supports throughout the research. Additionally, the author acknowledges Dr. Eisuke Saito, Dr. Anne Keary (Faculty at Monash University, Australia), and Dr. Melissa Barnes (La Trobe University, Australia) for their guidance on using inductive and deductive approaches in research.

Racism and Access to Maternal Health care Among Garo Indigenous Women in Bangladesh: A Qualitative Descriptive Study

Abstract

Racism as social determinant of health significantly affects Indigenous women's maternal healthcare access. This study uses Jones's 'Three Levels of Racism' theory and an intersectional lens to explore how racism shapes the experience of maternal health care access among Garo Indigenous women in Bangladesh. Semi-structured interviews were conducted with 24 women of diverse backgrounds and pregnancy statuses using snowball sampling. Thematic analysis, incorporating inductive and deductive approaches, was employed for data analysis. The findings reveal a significant deviation from Jones's theory regarding the level of internalized racism within the specific context of Garo Indigenous women's experiences. Jones's theory usually focuses on how racism is internalized due to institutional and personally-mediated factors. On the contrary, this study uncovers a unique theme: 'women agency.' This theme emerges as a robust response among the Garo Indigenous women to their encounters with institutional and personally-mediated racism, highlighting their cultural resistance and resilience. The findings suggest that the complex relationship between these two forms of racism contributes to the strengthening of agency among Garo Indigenous women. Their agency stems from avoiding hospitals that disrespect their culture, manifesting their cultural resistance practice against the encountered racism at the institutional and relational levels. To increase biomedical healthcare access among Garo Indigenous women, it is recommended to address racism through intercultural competency training with a 'cultural humility' approach. This approach would foster inclusivity and empowerment, recognizing the agency of Garo women in healthcare decisions. Additionally, it would facilitate constructive

dialogues between clinicians and Garo Indigenous women, acknowledging the shared experiences of exploitation within the latter group.

Introduction

Racism, defined as unfair treatment based on ethnic or cultural origin (1), significantly affects the maternal health outcomes of Indigenous women (2–5). While well-documented (3,5–7), its diverse impact on Indigenous women's access to maternal healthcare remains underexplored (8). Research from various countries indicates higher rates of maternal morbidity and mortality associated with racism (9–11). Instances of racial discrimination against Indigenous women by healthcare providers are evident in both India and the United States (12–15). Biomedical maternity care often exhibits racial bias and cultural insensitivity, resulting in trauma, mistrust, and reduced motivation among Indigenous women to seek healthcare (6,16). The intersection of gender, economic class, and other identities with racism further complicates Indigenous women's healthcare experiences (17–19), highlighting racism as a significant social determinant influencing their access to maternal healthcare (5,20). Racism as a system of privilege and oppression operates across institutional, personally-mediated, and internalized levels, resulting in an unequal distribution of power (21). This systemic racism perpetuates stereotypes, bias, and unequal access to resources (21,22), highlighting the absence of cultural humility approaches at the institutional and relational levels (23). Consequently, racism-driven discriminations have a substantial impact on maternal healthcare access (22,24–28). The patient-perceived discrimination scale is a valuable tool for investigating experiences within Indigenous communities (28). Additionally, understanding racially biased provider attitudes, particularly implicit bias, enhances awareness of interpersonal discrimination (29–31).

Racism against Indigenous identity is gaining recognition (32) but remains overlooked as a health issue in South Asian countries, including Bangladesh (33). Research reveals that racism significantly impacts Indigenous South Asian women's maternity care access, leading to health disparities and inadequate support (34–36). In Bangladesh, limited research focuses on Indigenous women's experiences with racism and maternity care (37,38), in contrast to global research in countries like the United States, Mexico, and India (9,31). Most research on healthcare access and racism centers on patients' perceptions of provider bias or implicit discrimination (39–44), with few exploring its direct health effects (39,45). These studies often lack a context-specific understanding of intersectionality and overlook the perspectives of Indigenous women who face compounded prejudice and implicit bias due to various aspects of their identity such as gender and economic class. To comprehensively grasp Indigenous women's maternity care experiences, it is imperative to consider their diverse identities within distinct social contexts (46).

This study examines how racism impacts Garo Indigenous women's experience of accessing biomedical maternal healthcare in Bangladesh. The Garo community is one of the largest Indigenous communities in Bangladesh (47), and one of the few close-knit communities worldwide (48). They follow a unique 'matrilineal-matrilocal' family structure (48). In this arrangement, family lineage and property are traced through the mother's line (49). Newly married couples reside with or near the wife's family, which grants women higher status and authority within the household and community (50). Since 2011, however, the government's postcolonial policies labeled the Garo community as 'tribal' instead of 'Indigenous' based on their culture and language, resulting in hierarchical distinctions, restricted access to rights and resources, and the reinforcement of cultural inferiority and colonial exclusionary practices (51–59). Despite their limited access to biomedical maternity care, racial biases in healthcare remain understudied (60–

62). Additionally, the scarcity of disaggregated data on indigenous women's healthcare access (63) underscores the necessity of this study.

The study employs Jones's 'three levels of racism' framework (22) along with Crenshaw's intersectional lens (19) to frame and analyze the themes emerged from participants' discussion. Jones's model categorizes racism into three levels: institutional, personally-mediated, and internalized (22). This breakdown helps providing a deeper understanding of the complex impact of racism on maternal healthcare access. Unlike models that primarily focus on the social construction of race or ethnicity and the structural factors perpetuating racial inequalities (64–66), Jones's comprehensive approach offers a holistic understanding. It suggests that institutional and personally-mediated racism contribute to the internalization of racism within the context of maternal healthcare access (22,28). Given its strong theoretical foundation, Jones's model is particularly relevant for our study.

Studies such as Janevic's research on Romani women (45) and similar work with Indigenous women (3,31,67,68), provide significant insights into the influence of racism on maternal healthcare access. However, these studies often neglect a crucial aspect: the intersection of racism with various identities, including gender and economic class. Despite its importance, the impact of this intersectionality on maternal healthcare access is frequently underemphasized (19,69). Maternal health research (70,71) underscores the significance of intersectionality, as it explores how social identities intersect to shape experiences, exacerbate healthcare disparities, and reveal interconnected systems of oppression (19). Hence, in alignment with Jones's 'Three Levels of Racism' theory (22), the current study utilized intersectionality (19) as an analytical framework. It facilitates a clear understanding of how racism intersects with gender and economic class, leading to diverse experiences of discrimination that influence maternal healthcare access

(19,69,72). This approach enhances the comprehension of healthcare inequalities and provides valuable insights for addressing the impact of racism on Garo Indigenous women's access to maternal healthcare.

Materials and methods

Settings

The study was conducted in Kalmakanda, a sub-district in northern Bangladesh. It primarily focused on the Garo people living in the unions of Lengura and Nazirpur (See Map 1) (73). Garo Indigenous women in these unions face challenges as they have limited access to maternal healthcare facilities, lacking Union sub-centers and community clinics (74,75). While the Upazila hospital has medical staff, including non-Garo male gynecologists, it is noteworthy that few Garo women seek care there. This situation prompts questions about the potential influence of racism in this context.

Map 1: Study area

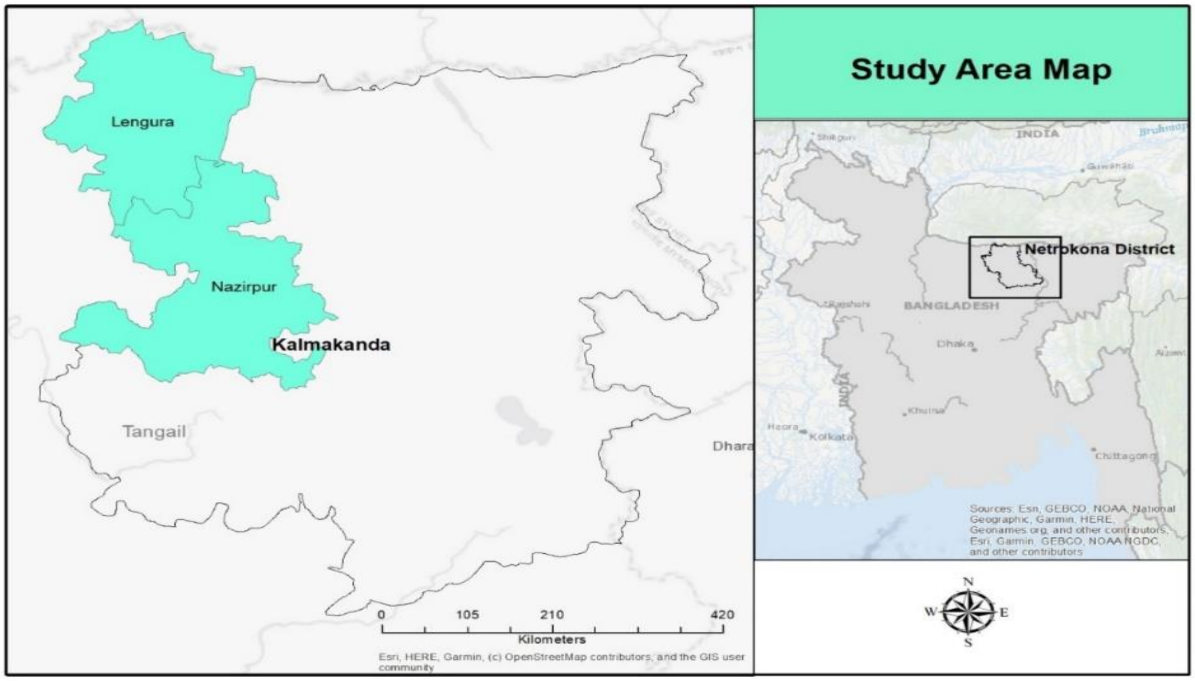

The author of the study is a male and holds the role of a researcher and primary investigator. He is from the mainstream non-Garo community in Bangladesh living outside of the study area. This makes him stranger when it comes to the place where the research was done and the participants who were part of it. The research site involves interactions with Garo Indigenous women seeking maternal health care. Being an outsider to the Garo community and coming from a different culture, the author emphasized the significance of culturally relevant approaches when engaging with the research subjects-Garo Indigenous women. To achieve this, the author hired female Garo research assistants on a paid basis to collect data since they have a cultural affinity with the participants, helping to establish cultural understanding and trust. This decision was based on the knowledge that cultural insiders could better connect with the participants and conduct the research responsibly (76). The author conducted three training sessions covering roles, rapport-building, and ethical considerations. Practical training included two demo interviews, enabling research assistants to gain the necessary skills for conducting interviews aligned with research objectives.

Study Procedures

This research was guided by the qualitative description approach (77). Qualitative description is widely used methodological approach, especially within practice disciplines (78). This approach was selected for this study as the technique of inquiry because it is typically applied to research driven by exploratory questions (79). The lack of in-depth research on maternal healthcare access among Garo indigenous women led to a need for an in-depth exploratory study. Qualitative description is notable for employing everyday language to create detailed summaries of findings (80). It facilitates detailed summarization of experiences or phenomena (80), assisting knowledge development in emerging fields (81). Given the nascent state of research on Indigenous women's

maternal healthcare access in Bangladesh (61,82), the outcomes of this qualitative description form a crucial basis for future research aimed at a broader theoretical understanding of the experience of racism among Indigenous women accessing maternal healthcare in Bangladesh.

Sampling and recruitment

The study used intersectionality-informed methods (83) to ensure a diverse sample by considering participants' overlapping identities and experiences. This approach captured the complexities of their lives and how social factors shape their perspectives (83). Thus, beyond traditional categories, the selection process considered identities as multidimensional, leading to a comprehensive understanding of participants' perspectives (19). Purposive and snowball sampling techniques, as the intersectionality-informed methods (84), were used to enroll 24 Garo Indigenous women (n=12 from Lengura Union and n=12 from Nazirpur Union) (85,86). It helps to address the challenges in identifying pregnant and new mothers (85). By combining purposive and snowball sampling methods and considering factors such as age, literacy, economic class, and pregnancy conditions (see Table 1 for inclusion criteria), the study ensured diversity. Also, it helps to represented the diverse perspectives of the participants. The author recognized historical challenges faced by Indigenous members in proving their Indigenous membership and did not require enrollment proof (87).

Table 1: Research area, number of IDI participants, and their inclusion criteria.

IDI respondents							
Area			*Number*	*Selection criteria*			
Netrokona District	Kalmakanda Upazila	Lengura Union	n=12	Age range	Household economic status	Educational level	Pregnancy diversity
				19-30	-Low-class<br>-Middle-Class<br>-High-class	Illiterate and Literate	The Garo women who were pregnant or had childbirth within 1 year before the date of taking the interview date
		Nazirpur Union	n=12				

Development of the Interview Guide

The author collaborated with Shila Gagra, a Garo school teacher in the study area during the study. Shila voluntarily provided valuable input in developing interview questions, ensuring cultural relevance. She also assisted in recruiting four Garo female research assistants and disseminating study findings.

Data Collection Procedure

Data were collected from February to March 2021 through semi-structured interviews and open-ended questionnaires, exploring maternal healthcare experiences and practices. Interviews lasted at least 50 minutes, were audio-recorded, and field notes were taken. The comprehensive training of the research assistants and their efforts in the structured data collection process ensured the acquisition of detailed and valuable insights into the respondents' experiences with maternal healthcare access. To ensure rigorous data collection, research assistants followed strategies of encouraging participants for open expression (88). They performed member-checking with willing participants, offering to provide a summary of findings for their review and feedback. This strengthened trust and credibility between the research assistants and participants.

Data Analysis

Virginia Braun and Victoria Clarke's six-step framework was used to guide the thematic analysis in this research, incorporating both inductive and deductive approaches (89). The rationale for employing both inductive and deductive methods lie in their complementary nature, as they allow for the use of theory to understand the data while also permitting the data to generate key themes/concepts that speak to the theory. Integration of both methods allowed an evaluation of

Jones's 'Three Levels of Racism' theory in the context of Garo Indigenous women, leading to novel insights that contribute to existing knowledge.

To enhance both the reliability and coherence of the findings, collaborative efforts were made with research assistants throughout the data analysis process. The interviews underwent transcription in Bengali, followed by translation into English, and cross-checking by both the author and research assistants. Immersion within the transcripts aimed to achieve a comprehensive understanding of racial experiences in the context of Jones's 'Three Levels of Racism' theory, with an openness to emerging themes. An inductive approach was employed to generate initial codes within each transcript, forming the basis for a systematic search for themes guided by Jones's theory, validated through collaboration with research assistants. These themes were defined, named, and aligned with Jones's theory, recognizing the emergence of new themes. The themes were presented to the participating Garo Indigenous women to ensure their alignment with the participants' perspective and viewpoint. Finally, the author writes the result section using the identified themes and relevant verbatim quotes from the transcripts. A summary of the results, written in Mandi language through collaborative efforts with research assistants, is shared with participants to gather their feedback for the final draft. Once consent is obtained, the author revisits the study area, invites all participants, and presents the final version of the research findings. Shila Gagra and research assistants assist the author in this process.

Trustworthiness

The triangulation between assistants and author during analysis; re-checking the codes and themes, member-checking of results with participants which has been abstracted thus far demonstrates the author's effort to ensure trustworthy of the research findings.

Ethical Consideration

This study, conducted as part of the author's MSc thesis at the Asian Institute of Technology (AIT), Thailand, received ethics approval from the AIT Ethics Committee. There was not local ethics committee from which the author required to seek approval. Before conducting interviews, participants and their husbands, as the local guardian, were informed about the research and provided verbal consent, following the approved approach. Research assistants from the Garo community established rapport and addressed concerns directly, leading to higher-quality approval. The prioritization of oral consent enhanced participant comprehension, engagement, and well-being, as reported by the research assistants, ultimately increasing the validity and reliability of the study. Confidentiality and voluntary participation were emphasized throughout the data collection process, ensuring participants were free to withdraw without consequences. Pseudonyms were utilized to protect privacy.

Results

Participants' characteristics

In-depth interviews (IDIs) were conducted with twenty-four Garo women. They typically expressed dissatisfaction with hospital-level healthcare access. Thematic saturation was achieved after nineteen IDIs, but to delve deeper, five more IDIs were conducted. The participants actively engaged and interacted effectively with the research assistants. Their ages ranged from 19 to 30 years, and they had diverse educational backgrounds and income levels. Most of them had at least one child. The author categorized their household income into three classes: high, middle, and low, based on their monthly household income in comparison to the national average of monthly household income, which was 15,988 BDT as per Bangladesh Bureau of Statistics (BBS) data (90).

Table 2: Participant profiles with pseudonyms from Lengura Union

Place of residence	Participant	Age	Household economic Status	Educational Level	Pregnancy diversity
Lengura Union	L1	21	Low-class	Illiterate	She had 2 children and was in the third trimester of her pregnancy during the interview.
	L2	30	Middle-Class	Illiterate	She had 4 children, and her most recent childbirth occurred within eight months before the interview date.
	L3	22	Middle-Class	Illiterate	She had 3 children and was in the first trimester of her pregnancy during the interview.
	L4	25	High-Class	She finished primary school education	She had 1 child and was in the first trimester of her pregnancy during the interview.
	L5	19	Middle-Class	Illiterate	She was in the second trimester of her pregnancy during the interview and had no previous experience of pregnancy
	L6	20	Low-Class	Illiterate	She had 1 child and was in the second trimester of her pregnancy during the interview.
	L7	27	Low-Class	Illiterate	She had 3 children, and her most recent childbirth occurred within five months before the interview date.
	L8	20	Middle-Class	Illiterate	She had 2 children and was in the second trimester of her pregnancy during the interview.
	L9	21	High-class	She finished primary school education	Had no previous experience of pregnancy and her most recent childbirth occurred within two months before the interview date.
	L10	23	Low-Class	Illiterate	She had 2 children, and her most recent childbirth occurred within nine months before the interview date.
	L11	19	High-Class	She finished primary school education	She had no prior pregnancy experience was in the second trimester of her pregnancy during the interview.
	L12	26	Low-Class	Illiterate	She had 3 children and was in the third trimester of her pregnancy during the interview.

Table 3: Participant profiles with pseudonyms from Nazirpur Union

Place of residence	Participant	Age	Household economic Status	Educational Level	Pregnancy diversity
Nazirpur Union	N1	20	Middle-Class	Illiterate	She had 1 child and was in the second trimester of her pregnancy during the interview.
	N2	20	Middle-Class	Illiterate	Had no previous experience of pregnancy and her most recent childbirth occurred within four months before the interview date.
	N3	22	High-Class	She finished primary school education	She had 1 child and was in the third trimester of her pregnancy during the interview.
	N4	20	Middle-Class	Illiterate	Had no previous experience of pregnancy and her most recent childbirth occurred within three months before the interview date.
	N5	24	Low-Class	Illiterate	She had 2 children, and her most recent childbirth occurred within six months before the interview date.
	N6	20	Low-Class	Illiterate	She had no prior pregnancy experience was in the third trimester of her pregnancy during the interview.
	N7	21	Low-Class	Illiterate	She had 1 child and was in the second trimester of her pregnancy during the interview.
	N8	23	Low-Class	Illiterate	She had 2 child and was in the second trimester of her pregnancy during the interview.
	N9	20	Low-Class	Illiterate	She had 1 child, and her most recent childbirth occurred within seven months before the interview date.
	N10	21	Low-Class	Illiterate	Had no previous experience of pregnancy and her most recent childbirth occurred within three months before the interview date.
	N11	20	Middle-Class	She finished primary school education	She had 1 child, and her most recent childbirth occurred within six months before the interview date.
	N12	24	High-Class	She finished primary school education	She had 1 child and was in the third trimester of her pregnancy during the interview.

Figure 1 presents ten themes identified through open-coding, categorized into three main groups and analyzed using an intersectional approach. These three categories are: ‘institutional racism,’ ‘personally mediated racism,’ and ‘women's agency.’

The first two categories correspond to Jones’s institutionalized and personally mediated racism levels. When discussing about their experience of institutional and personally mediated

racism in this study, the Garo women recurrently pointed out the issue of exploitation. Their interactions with hospital staff consistently revealed instances of exploitation, a shared concern within the group. While experiences were consistent among participants, the author recognizes individual nuances, emphasizing the need to address and improve the overall maternity care experiences of Garo Indigenous women.

The third category, ‘women's agency,’ emerged as a response to the first two categories. This category is unique in this study. Jones’s theory suggests that institutional and personally mediated racism leads to the internalization of racism (22,45). On the contrary, in this study evidence demonstrates that these two levels of racism strengthen Garo Indigenous women’s agency of cultural resistance practices against the encountered institutional and personally mediated racism.

Figure 1: Institutional racism, personally mediated racism, and women agency in maternal healthcare

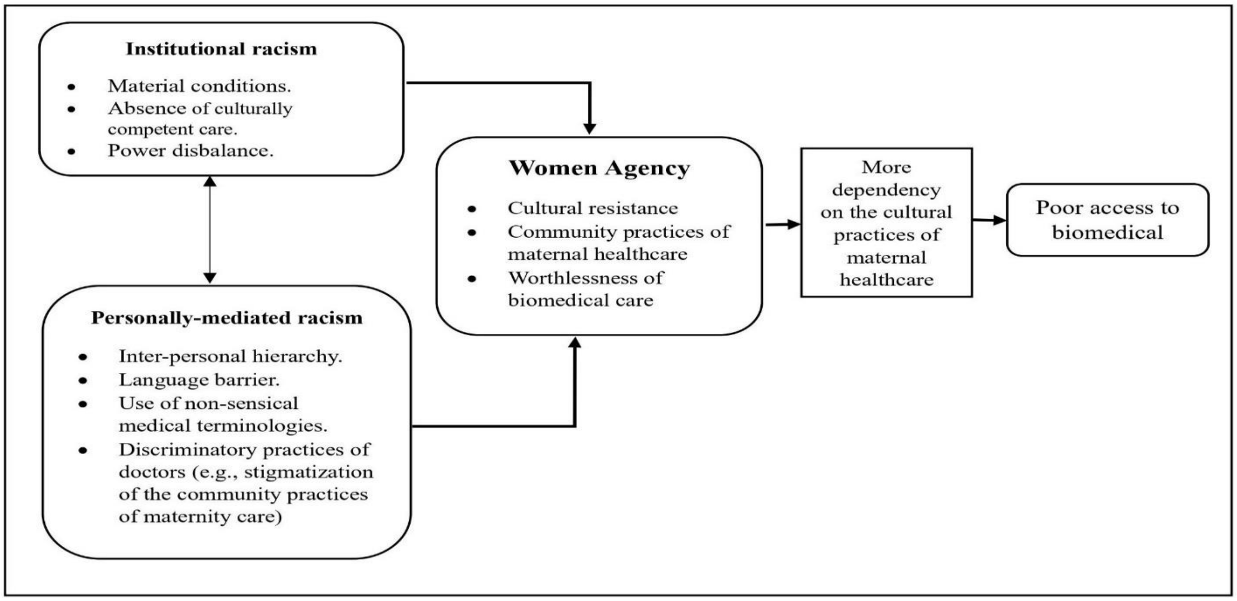

Institutional racism

Institutional racism is evident in material conditions, culturally inappropriate care, and resulting power imbalances. Regardless of economic class and education level, participants faced challenges accessing maternal healthcare due to clinicians' claims of insufficient resources and power imbalances. For example, one participant reported clinicians' using the excuse of unavailability of medical facilities and limited supplies for discriminatory care at the hospital. As she typically discussed:

> During my last pregnancy, I saw a big difference in how doctors treated non-Garo women compared to me at the hospital. They got good care with all they needed, but the doctor said they didn't have enough for me. I was surprised when a non-Garo woman got proper care for the same problem. In our community, it's hard to access good medical facilities, so getting the care we need during pregnancy is tough. Doctors often use shortages of machines and staffs as excuses, making things even harder for us (N4).

This Participant described discrimination in care compared to non-Garo women at the hospital, suggesting healthcare service disparities based on Indigenous identity. Non-Garo women received proper care, while the participants faced excuses about insufficient resources, raising concerns about institutional racism. Reflecting similar views with other participants, another participant felt ignored by clinicians when seeking biomedical care due to their focus on non-Garo patients. As she reported: "due to the gynecologists' preference for non-Garo women, pregnancy care from hospital becomes impossible for us. But we don't expect this (L8)". When asked if experiencing discrimination at the hospital prevented Garo Indigenous women from seeking maternal healthcare, another woman typically responded: "Yes... Honestly, I can't recall a time

when I went to a doctor’s office for maternal care after facing discrimination during my first visit (L3)” As this Participant highlights, lacking access to equitable healthcare treatment often translates to limited access to medical professionals. Consequently, the women interviewed commonly pointed out inadequate services, discriminatory treatment, and clinicians' preference for local women.

Furthermore, all the participants consistently voiced concerns regarding the hospital’s lack of culturally appropriate care. This was particularly troubling for them as receiving biomedical care conflicting with their community's maternal healthcare practices was not acceptable. As one of them typically shared:

> I felt deeply troubled by the lack of alignment between the available healthcare at the hospital and our community’s pregnancy healthcare practices. Following our cultural traditions is essential to us, and it was challenging for me to comprehend and accept healthcare practices that didn't respect our customs. It left me feeling confused and uneasy. (L6).

This participant’s troubled, confused, and uneasy feelings indicate a dissonance between their expectations and the care provided. Another participant also mentioned that culturally inappropriate care includes not being able to meet the birth attendant before delivering in a hospital, which goes against their cultural norms and expectations:

> I don’t understand why the doctors won’t let us meet the midwife before giving birth. It’s essential for us, and they don't seem to care about our cultural needs. They mistreat us, saying they can’t do anything because of the hospital's rules (N6).

In addition, another woman with a common experience similar to other participants expressed about the birthing dress code: “we can’t go inside the delivery room without wearing the hospital's

dress, given by the doctors. It's unfair and against our norms related to birth delivery because many women already wear their clothes during delivery (L1)".

While discussing the reasons behind the absence of culturally appropriate care, the participants generally pointed out issues such as clinicians' inadequate cultural training, lack of community engagement, and limited knowledge about traditional healing practices. As one of the participants generally discussed:

> I think the doctors don't understand our ways and beliefs. They don't involve our community in decisions. Our traditions are ignored, making us feel disconnected. They don't know much about our healing practices, so they prefer their own ways (L12).

This participant discussed her frustration and dissatisfaction with the healthcare system's lack of cultural understanding and involvement of their community in decision-making. She also expresses disconnection and alienation due to disregarding their traditions and healing practices. Interestingly, she and other low-income women also stressed the importance of female clinicians. In contrast, many other participants linked the absence of Garo female doctors to culturally inappropriate care, highlighting the gender sensitivity of maternal health issues:

> I am deeply concerned about absence Garo female doctors. The non-Garo doctors does not have any care and respect for us and seeking care from non-Garo male doctor is against out our culture because we can't share our health problem to a male doctor (L5).

In addition, unlike the participants from the high-class household income group, the Garo women from the middle-class and low-class household income statuses highlight the hidden costs of seeking medical treatment, such as transportation expenses and expensive hospital food. The disparity between the claimed cost and hospital charges points to potential financial exploitation.

The lack of control over travel and treatment costs further adds to the burden on these women. The participant's frustration with the hospital's dismissive attitude toward their concerns reflects a systemic issue of healthcare institutions not adequately addressing the needs and challenges faced by Indigenous communities. As one of the participants shared:

> Distance cost isn't all. Hospitals want money. Despite their claim, they don't charge 5 Taka (Bangladeshi currency). They demand more. Traveling makes you hungry, but food is expensive. We must spend all we earn if we go. We don't control travel and treatment costs. They ignore our issue. They said 'you have to follow the hospital rules' whenever we told them our problem. What else can we do but avoid it? (L2).

This participant's discussion highlights Indigenous women's financial obstacles when seeking healthcare, including hidden costs and potential hospital exploitation. It serves as an example of financial cost-driven discrimination in biomedical maternity care, highlighting how institutionalized ethnocentrism impacts access to healthcare for Garo women.

Overall, the participants typically blamed insufficient human resources, culturally inappropriate care, and the dominant power of the clinicians for poor access. Also, they present the obligation of following the institutional guidelines defined by the clinicians as a reason for not accessing these services. This illustrates a link to personally-mediated racism, as presented in the following sub-section.

Personally-mediated racism

Garo participants faced personally-mediated racism from clinicians, evident in the inter-personal hierarchy, use of non-sensical medical terms, and discriminatory practices like stigmatizing community maternity care practices. Language-related racism was also present, with clinicians

viewing Garo participants' use of the Mandi language as problematic, leading to miscommunication and discrimination against indigenous language speakers for not using mainstream language:

> They (the clinicians) don't understand what we're saying. We don't want to be treated by him because he's aggressive and doesn't know how. Our Garo women went there with health issues like jak jachak dalya (swelling of feet and hands), wakkalna ha'sika (vomiting), matha betha (dazziness), Hapani (shortness of breath), and bikma chikki sadika (abdominal pain), but the doctor didn't listen. They give us medicine even though they don't understand (N5).

This participant shared troubling experiences with clinicians lacking cultural understanding and empathy, expressing frustration with their aggressive and dismissive behavior, leaving her feeling unheard and misunderstood. Other participants reported similar experiences, even those from high-class households with primary education. For instance, bilingual Garo women faced discrimination at the hospital due to difficulty pronouncing Bengali words. Using Bengali in the hospital was emotionally and practically harder than in daily life, and clinicians' unfamiliar medical terminology added to their challenges. As one of the participants shared:

> Though I can't pronounce Bengali words well, I can explain my health issues to doctors. But, I'm hesitant to go again because the doctors laughed at me when I talked about my health issues a few months ago. The doctors replied with medical jargon. He laughed again when I asked him to explain these. I felt insulted (L7).

As this participant discussed, she encounters challenges as an Indigenous woman seeking healthcare. Despite her efforts to communicate in Bengali, the clinicians' dismissive attitude and use of medical jargon created a barrier to understanding. Their laughter made her feel demeaned

and disrespected. This reflects a broader cultural issue where language barriers and lack of empathy hinder effective communication and care. Additionally, participants highlighted the issue of power and domination over the Garo women's bodies. They mentioned clinicians using force, touching private organs without permission, and not allowing the family to perform cultural rituals with the placenta, which were significant barriers to respectful maternal healthcare. One mother typically reported: "Our culture forbids delivering babies by cutting the belly. They touch our private organ during mid-pregnancy visits. Permission was never sought. They forcefully violate our culture (N2)."

The participant's quote indicates that clinicians exerted power to ignore the Garo women's cultural norms for maternity care and pressured them to adopt biomedical care as a symbol of modernity. The women's accounts also revealed that clinicians' perception of community maternal healthcare practices stigmatized them, creating obstacles in accessing the hospital:

> The doctors don't listen to our cultural needs, instead blaming us for our customs, such as not cutting the belly and reciting the delivery mantra. They said we are responsible for our health complications, especially birthing complications (N5).

Participants generally shared experiences of clinicians' discriminatory practices, stigmatizing them for adhering to community maternity healthcare. Many feared hospital visits due to concerns of forced C-sections or vaginal incisions, violating cultural norms and reproductive health. Clinicians' ethnocentrism blamed severe maternal health complications on community practices, disregarding Indigenous culture and pushing for biomedical control. Thus, the doctor-Garo mother hierarchy is revealed:

> Whenever to visit the doctor, they stigmatize us for our belief in our community norms related to pregnancy health care. Disrespecting our community practices of not cutting the belly for delivery, they forced us to cut the belly. In fact, the doctor decides how to deliver, not we (N9).

The data shows that clinicians' preference for C-sections for Garo women reflects institutionalized racism, disregarding the cultural belief in the safety of normal birthing. This interplay between institutional and personally-mediated racism shapes internalized racism among the Garo Indigenous women, as discussed in the following sub-section.

Women Agency

The sub-themes that emerged this category include, cultural resistance, community practices of maternal healthcare, and perceived worthlessness of biomedical care.

Concerning agency, while discussing the effect of the previous experience of being stigmatized, a mother typically reported her agency in deciding to avoid seeking biomedical care:

> Now, I do things my way when it comes to taking care of myself during pregnancy. I don't go to the hospital because they don't understand us. They make me feel small and don't listen to what I say. (N5).

This participant's discussion reflects empowerment through embracing traditional practices rooted in her cultural heritage. Exercising agency means she actively made choices based on her cultural beliefs, not just accepting the mainstream healthcare approach. She shows resilience in navigating a healthcare system that often fails to understand her unique needs. Her decision to avoid biomedical care is a response to the stigma and lack of respect she experienced in that setting. When asked, in addition to clinicians' stigmatization and discrimination, what else helps facilitate

such agency to decide to avoid seeking biomedical care, she replied about her community bonding and support as another source for that:

> No, it's not just because of doctors' blaming and discrimination. Also, because our community women who are supportive to each other. Our elders know so much, and I trust them. All these gave me courage to decide to avoid going hospital (N5).

Interestingly, the interviewed women linked their perceived agency to avoid going hospital with the worthlessness of biomedical care compared to community practices of maternity care. Institutionalized racism and clinicians' personally-mediated racism, shown in power hierarchies and stigmatization, influenced their decision to rely more on community practices. One participant shared:

> Hospital-level care is value-less to me because they disrespect us. When I need help with my health, I go to our traditional healers. They know our beliefs and take care of us with respect. I want to keep our traditions alive and stay connected to who we are (L7).

This participant's experience of institutionalized and personally-mediated racism shows how it becomes a source of culturally empowered agency to internalize the comparative benefits of community practices in maternal healthcare. For this participant, being sure to be culturally humiliated at the hospital was something she saw as a sign of strength to decide on an alternative source of healthcare. When asked what would have happened if she had not been able to get cured of possible maternal health complications, she responded:

> I knew from my neighbor women and mother, who had their previous pregnancy experiences that if I follow our cultural practices, then there will be no pregnancy health illness. So, why should I visit hospital to be humiliated and encounter discrimination? (L7).

When asked about the types of their community practices of maternal healthcare, along with this participant, another woman typically shared:

> We have several norms for a safe pregnancy, including avoiding houses where someone has died, not using mouth to fan fire smoke while cooking, eating locally sourced and culturally specific foods, spending time outdoors, participating in traditional activities, and seeking solace in natural environments. We also follow rituals and ceremonies related to pregnancy and rely on medicinal herbs and plants for managing pregnancy discomforts, promoting healthy labor, and aiding postpartum recovery. Additionally, we seek care from traditional healers and birth attendants within our own community (L8).

This participant reported the importance and her dependency on the community practices of maternal healthcare as the manifestation of her resistance practices against the clinicians' discriminatory practices as a means to offset the clinician's intention of stigmatizing her. Similar to other respondents, another participant felt that as she encountered discrimination at the hospital, she would not have been able to resist such racism against her without increasing her dependency on traditional healers whom she found far better:

> I was able to avoid getting stigmatized by the doctors when I started following our community norms of pregnancy healthcare, I had good health care without making further visit to the hospital. ….I went to traditional healer to all my prenatal visits…I had it good (N11).

Thus, the participants' experiences indicate that the internalization of the intersectional mix of institutional and personally-mediated racism by clinicians led to feelings of disempowerment and self-devaluation at hospitals. They perceived biomedical care as less valuable than community practices of maternal healthcare, which they found non-discriminatory. As a result, they sought

cultural empowerment and resistance through community-based maternity care to avoid discrimination and stigmatization at hospitals.

Discussions

This qualitative descriptive study investigates the impact of racism on Garo Indigenous women's maternal healthcare experiences, employing Jones's 'Three Levels of Racism' theory (22) and an intersectionality approach (19). Nevertheless, the study's results, as outlined in the previous section, reveal a significant deviation from Jones's theory regarding the three levels of racism within the specific context of Garo Indigenous women's experiences. Jones's theory usually focuses on how racism is internalized due to institutional and personally-mediated factors. On the contrary, this study uncovers a unique theme: 'women agency.' This theme emerges as a robust response to institutional and personally-mediated racism within the Indigenous women studied, highlighting their cultural resistance and resilience. The findings suggest that the complex relationship between these two forms of racism contributes to the strengthening of agency among Garo Indigenous women. This, in turn, shapes their preference for community-based maternal healthcare, which differs from a previous study (45).

Additionally, the current study uncovers the persistent and multifaceted discrimination faced by Garo Indigenous women in accessing maternity care, driven by institutional racism (22). Participants generally highlight issues like lacking culturally competent care and power imbalances due to Indigenous identity-based racism at the institutional level. The exclusionary practices in healthcare, justified by resource constraints, reinforce institutional racism. This finding adds to a prior study that examined how Indigenous identity-based racial discrimination is ignored by institutions providing biomedical care services (33). However, it contrasts with prior research

in Ethiopia, where actual unavailability reduced Indigenous maternal healthcare access, indicating context-specific variations (41,42).

The author's use of intersectionality (19) within Jones's framework of institutional racism (22) to analyze participant accounts suggests the connected and overlapping aspects of discrimination driven by these dimensions of institutional racism that specifically affect participants' access to biomedical healthcare. Intersectionality demonstrates how these women's social background, Indigenous identity, and gender interact (19). Race and socioeconomic position interact in the Upazila hospital's care and resource distribution, as the participants highlighted. Gender and Indigenous identity intersect to discriminate against these women in healthcare as seen by the lack of Garo female doctors and the preference for non-Garo male doctors. Hidden costs and financial exploitation are experienced by Garo women from economically underprivileged communities, demonstrating racial and financial bias. As the participants discussed, institutional racism results from the clinicians' dominating posture in forcing them to follow hospital policies. This position shows a power imbalance between men and women and is based on race. These findings are well supported by previous studies highlighting the intersectionality among race, gender, and socio-economic status of Indigenous women (17–19).

The participants' stories make clear the ingrained stereotypes and discriminatory practices that Garo Indigenous women must deal with within the healthcare system. Jones's theses of personally-mediated racism (22) which takes into account inter-personal hierarchy and asymmetric power relations based on 'othering' (56), fits issues like inter-personal hierarchy, language obstacles, and discriminatory practices of clinicians as encounters by the Garo women. The power relations between biomedical institutions and Indigenous women regarding maternity care have been the subject of prior studies conducted in several nations (27,31,43). In this study, also, this

tendency is also evident. As a result of Bangladesh's health policy, which perpetuates colonial stereotypes by classifying Indigenous people as a separate tribe, clinicians are unable to fully comprehend the imbalances in power that exist with Garo women (58,59), It prevents clinicians from understanding historical and contemporary contexts and identifying unequal power relations with Garo women. Thus, participants' discussion suggests that clinicians following service guidelines reproduce colonial prejudices and stereotypes in an institutionalized context. It means the levels of racism against Indigenous Garo women often reinforce each other.

Accordingly, an intersectional analysis of the participants' discussion shows that their experiences, similar to institutional racism, are influenced not only by their Indigenous identity but also by their gender, culture, and socioeconomic level (19). The difficulties and prejudices people confront in the healthcare system are exacerbated by these interconnected identity characteristics. The participants' discussions shed light on how personally-mediated racism intertwines with an inter-personal power hierarchy, resulting in ongoing discrimination. Nonsensical medical jargon and discriminatory attitudes toward the Garo language exacerbate their marginalization.

Furthermore, the clinicians' racist approach and lack of consideration for cultural norms during pregnancy and childbirth underscore the impact of race and culture on healthcare access. Other studies have shown how segregationist colonialism and assimilationist policies have influenced relations between public institutions and Indigenous communities (32,44), creating a racialized cultural logic of intercultural connections (31). This study adds to these findings, demonstrating that racism intersects with other forms of discrimination (19). For instance, the dominance of Western medical perspectives and stigmatization of Garo women for following their community's maternity healthcare practices highlight the intersection of race, cultural norms, and

colonial legacies, leading to gendered power hierarchies and clinicians' attitudes toward Garo women. Moreover, the fear of forced medical procedures and clinicians' decisions further emphasize the intersectionality of race, gender, and power hierarchies, perpetuating vulnerabilities among Indigenous women within the healthcare system. These findings contribute to previous research (28), deepening the understanding of discrimination faced by Indigenous women.

The participants' discussion on 'agency' reveals its profound impact on their healthcare. Themes like cultural resistance, community practices, and perceived worthlessness of biomedical care show how it shapes their decisions and cultural preservation. They resist biomedical care due to stigmatization, embracing community-based practices to strengthen their cultural identity. This contrasts with a study from Mexico where women abandoned Indigenous practices for biomedical care (31). The intersectional analysis highlights that the participants' agency is not solely derived due to individual attitudes but also institutional practices, policies, and personally-mediated racism.

To sum up, this study uncovers the multifaceted impact of racism on Garo Indigenous women's healthcare access. Through the lens of intersectionality, it becomes evident that race, gender, culture, and socioeconomic status intersect to compound the barriers they face. This intersectional perspective deepens our understanding of institutional and personally-mediated racism, shedding light on how it shapes the agency of these women. The study contributes to the previous research on racism and healthcare (3,31,45,68), emphasizing the need to address both individual prejudices and systemic inequities in healthcare systems.

The study shows based on the evidence of the reports of Garo women: there is strong racism at different levels, and it strongly compromises biomedical healthcare access. Garo women

count on a strong traditional medicine system to attend maternal healthcare. Garo women agency derives in their decision to avoid hospitals were they and their culture is mis-respected. Therefore, to increase biomedical healthcare access for Garo women racism needs to be present in the institutional and relational level.

Therefore, the current study suggests for implementing intercultural competency training program (CICTP) for the clinicians. This program should incorporate 'cultural humility' approaches, which recognize and respect intersecting identities of race, gender, cultural identity, and socioeconomic status (23). This approach aims to address power imbalances at personal and structural levels of racism (23). Implementing programs designed with cultural humility principles (23) can create an inclusive and empowering environment for Garo Indigenous women, acknowledging their agency in healthcare decisions. Furthermore, it would help initiate constructive dialogues with between the clinicians and the Garo Indigenous women to address the latter group's shared experience of exploitation.

Conclusion

Racism is a vital maternity health concern for Garo Indigenous women, with institutional racism and personally-mediated racism manifesting in discrimination and stigmatization due to language and community cultural norms related to maternal health care. This study presents the multi-dimensional nature of racism, as typically discussed by the participants, and how it impacts their maternal healthcare access. The findings emphasize the significance of intersectionality, showcasing how institutionalized racism and personally-mediated racism generates Garo Indigenous women's agency and thus, shape the experiences of maternal healthcare. Furthermore, it underscores the importance of recognizing and respecting intersecting identities of race, gender,

cultural identity, and socioeconomic status to address power disbalances at both personal and structural levels of racism. It highlights the need for clinicians' intercultural competency training programs incorporating 'cultural humility' approaches to create an inclusive and empowering healthcare environment for Garo Indigenous women that ensure equity in healthcare access.

References

1. Shiraev EB, Levy DA. Cross-cultural psychology: Critical thinking and contemporary applications. Routledge; 2020.

2. Creanga AA, Bateman BT, Kuklina E V, Callaghan WM. Racial and ethnic disparities in severe maternal morbidity: a multistate analysis, 2008-2010. Am J Obstet Gynecol. 2014;210(5):435-e1.

3. Nuru-Jeter A, Dominguez TP, Hammond WP, Leu J, Skaff M, Egerter S, et al. "It's the skin you're in": African-American women talk about their experiences of racism. An exploratory study to develop measures of racism for birth outcome studies. Matern Child Health J. 2009;13:29–39.

4. Petersen EE, Davis NL, Goodman D, Cox S, Syverson C, Seed K, et al. Racial/ethnic disparities in pregnancy-related deaths—United States, 2007–2016. Morbidity and Mortality Weekly Report. 2019;68(35):762.

5. Mateo CM, Williams DR. Racism: a fundamental driver of racial disparities in health-care quality. Nat Rev Dis Primers. 2021;7(1):20.

6. Feagin J, Bennefield Z. Systemic racism and US health care. Soc Sci Med. 2014;103:7–14.

7. Davis DA. Reproductive injustice: Racism, pregnancy, and premature birth. Oxford University Press; 2020.

8. Jordal M, Wahlberg A. Challenges in providing quality care for women with female genital cutting in Sweden–A literature review. Sexual & reproductive healthcare. 2018;17:91–6.

9. Poetry S. Giving Birth in South Florida : A Phenomenological Study of the Pregnancy and Birthing Experiences among Black and Afro-Latina Women. 2020.

10. Petersen EE, Davis NL, Goodman D, Cox S, Syverson C, Seed K, et al. Racial/ethnic disparities in pregnancy-related deaths—United States, 2007–2016. Morbidity and Mortality Weekly Report. 2019;68(35):762.

11. Dossetor PJ, Freeman JM, Thorburn K, Oscar J, Carter M, Jeffery HE, et al. Health services for aboriginal and Torres Strait Islander children in remote Australia: A scoping review. PLOS Global Public Health. 2023;3(2):e0001140.

12. Thapa R, Teijlingen E Van, Regmi PR, Heaslip V. Caste Exclusion and Health Discrimination in South Asia : A Systematic Review. 2021;

13. Kozhimannil KB, Interrante JD, Tofte AN, Admon LK. Severe maternal morbidity and mortality among indigenous women in the United States. Obstetrics and gynecology. 2020;135(2):294.

14. MacDonald C, Steenbeek A. The impact of colonization and western assimilation on health and wellbeing of Canadian aboriginal people. International Journal of Regional and Local History. 2015;10(1):32–46.

15. Adhikari T, Sahu D, Nair S, Saha KB, Sharma RK, Pandey A. Factors associated with utilization of antenatal care services among tribal women: A study of selected States. Indian J Med Res. 2016;144(1):58.

16. Küey L. Clarifying Definitions of „Race ", Racism, and Ethnocentrism. European Psychiatry. 2022;65(S1):S35–S35.

17. Varcoe C, Brown H, Calam B, Harvey T, Tallio M. Help bring back the celebration of life: A community-based participatory study of rural Aboriginal women's maternity experiences and outcomes. BMC Pregnancy Childbirth. 2013;13(1):1–10.

18. Anne Van Herk K, Smith D, Andrew C. Identity matters: Aboriginal mothers' experiences of accessing health care. Contemp Nurse. 2011;37(1):57–68.

19. Crenshaw K. Demarginalizing the intersection of race and sex: A black feminist critique of antidiscrimination doctrine, feminist theory and antiracist politics. u Chi Legal f. 1989;139.

20. Figueroa JF, Frakt AB, Jha AK. Addressing social determinants of health: time for a polysocial risk score. JAMA. 2020;323(16):1553–4.

21. Paradies Y. A systematic review of empirical research on self-reported racism and health. Int J Epidemiol. 2006;35(4):888–901.

22. Jones CP. Levels of racism: a theoretic framework and a gardener's tale. Am J Public Health. 2000;90(8):1212.

23. Tervalon M, Murray-Garcia J. Cultural humility versus cultural competence: A critical distinction in defining physician training outcomes in multicultural education. J Health Care Poor Underserved. 1998;9(2):117–25.

24. Reddy S, Patel N, Saxon M, Amin N, Biviji R. Innovations in US health care delivery to reduce disparities in maternal mortality among African American and American Indian/Alaskan Native women. J Patient Cent Res Rev. 2021;8(2):140.

25. Allen L, Wodtke L, Hayward A, Read C, Cyr M, Cidro J. Pregnant and early parenting Indigenous women who use substances in Canada: A scoping review of health and social issues, supports, and strategies. J Ethn Subst Abuse. 2022;1–31.

26. Lawford KM, Giles AR, Bourgeault IL. Canada’s evacuation policy for pregnant First Nations women: resignation, resilience, and resistance. Women and Birth. 2018;31(6):479–88.

27. Hayward A, Cidro J. Indigenous birth as ceremony and a human right. Health Hum Rights. 2021;23(1):213.

28. Akinade T, Kheyfets A, Piverger N, Layne TM, Howell EA, Janevic T. The influence of racial-ethnic discrimination on women’s health care outcomes: A mixed methods systematic review. Soc Sci Med. 2023;316:114983.

29. Zestcott CA, Blair I V, Stone J. Examining the presence, consequences, and reduction of implicit bias in health care: a narrative review. Group Processes & Intergroup Relations. 2016;19(4):528–42.

30. Hassen N, Lofters A, Michael S, Mall A, Pinto AD, Rackal J. Implementing anti-racism interventions in healthcare settings: a scoping review. Int J Environ Res Public Health. 2021;18(6):2993.

31. Dörr NM. Health professionals’ perception of maternal health: an ethnography in the Sierra de Totonacapan, Veracruz, Mexico. Social Medicine. 2020;13(3):155–65.

32. Butler R, Ben J. Centring settler colonialism in rural Australian multicultures: race, place and local identities. J Ethn Migr Stud. 2021;47(9):2179–97.

33. Rice ZS, Liamputtong P. Culture and Global Health: Cultural Determinants of Health and Well-Being. Handbook of Global Health. 2020;1–26.

34. Akter S. Examining access to health services for women of reproductive age from ethnic communities in Chittagong Hill Tracts (CHT) of Bangladesh.

35. Thresia CU, Srinivas PN, Mohindra KS. The Health of Indigenous Populations in South Asia : A Critical Review in a Critical Time. 2022;

36. Akter S, Davies K, Rich JL, Inder KJ. Barriers to accessing maternal health care services in the Chittagong Hill Tracts, Bangladesh: A qualitative descriptive study of Indigenous women’s experiences. PLoS One. 2020;15(8):e0237002.

37. Chowdhury SK. Uptake of antepartum care services in a matrilineal-matrilocal society: a study of Garo indigenous women in Bangladesh. BMC Pregnancy Childbirth. 2023;23(1):75.

38. Akter S, Davies K, Rich JL, Inder KJ. Community perspectives of barriers indigenous women face in accessing maternal health care services in the Chittagong Hill Tracts, Bangladesh. Ethn Health. 2022;27(5):1222–40.

39. Dehon E, Weiss N, Jones J, Faulconer W, Hinton E, Sterling S. A systematic review of the impact of physician implicit racial bias on clinical decision making. Academic Emergency Medicine. 2017;24(8):895–904.

40. FitzGerald C, Hurst S. Implicit bias in healthcare professionals: a systematic review. BMC Med Ethics. 2017;18(1):1–18.

41. Khosla R, Zampas C, Vogel JP, Bohren MA, Roseman M, Erdman JN. International human rights and the mistreatment of women during childbirth. Health Hum Rights. 2016;18(2):131.

42. Ibrahima AB. Exploring Maternal Health in Ethiopia Using Indigenous Approaches: Policy and Practice Implications. Res Soc Work Pract. 2021;31(4):343–50.

43. Yeung S. Conceptualizing cultural safety: Definitions and applications of safety in health care for Indigenous mothers in Canada. Journal for Social Thought. 2016;1.

44. Moran A. White Australia, settler nationalism and Aboriginal assimilation. Australian Journal of Politics and History. 2005;51(2):168–93.

45. Janevic T, Sripad P, Bradley E, Dimitrievska V. " There's no kind of respect here" A qualitative study of racism and access to maternal health care among Romani women in the Balkans. Int J Equity Health. 2011;10(1):1–12.

46. Khosla R, Zampas C, Vogel JP, Bohren MA, Roseman M, Erdman JN. International human rights and the mistreatment of women during childbirth. Health Hum Rights. 2016;18(2):131.

47. Tamanna S, Rana MM, Ferdoushi A, Ahmad SAI, Rahman M, Rahman A. Assessment of nutritional status among adolescent Garo in Sherpur District, Bangladesh. Bangladesh Journal of Medical Science. 2013;12(3):269–75.

48. Hasan M, Momtaz P, Hosen I, Das SA, Akhteruzzaman S. Population genetics of 17 Y-chromosomal STRs loci in Garo and Santal tribal populations in Bangladesh. Int J Legal Med. 2015;129(2):251–2.

49. Dey S, Resurreccion BP, Doneys P. Gender and environmental struggles: voices from Adivasi Garo community in Bangladesh. Gender, Place & Culture. 2014;21(8):945–62.

50. Das TK, Islam S. Psycho-social dimensions of ethnicity: The situation of Garo community in Bangladesh. Asian Aff (Lond). 2005;27(3):45–54.

51. Das DrTK. Psycho-Social dimensions of ethnicity : The situation of the Garo community in Bangladesh. Journal of Ethnic Affairs [Internet]. 2005;I(1):5–10. Available from: www.ecdo-bd.org

52. Islam MR, Islam MA, Banowary B. Determinants of exposure to mass media family planning messages among indigenous people in Bangladesh: A study on the Garo. J Biosoc Sci. 2009;41(2):221–9.

53. Rahman A. Fighting Exclusion: Towards Understanding the Predicament of Adivasis in Bangladesh. In: Political Governance and Minority Rights. Routledge India; 2020. p. 104–20.

54. Bal E, Chambugong TC. The Borders that Divide, the Borders that Unite: (Re)interpreting Garo Processes of Identification in India and Bangladesh. J Borderl Stud. 2014;29(1):95–109.

55. Bal E. Becoming the Garos of Bangladesh: Policies of exclusion and the ethnicisation of a ‘tribal’minority. South Asia: Journal of South Asian Studies. 2007;30(3):439–55.

56. Spivak G. In Other Worlds: Essays in Cultural Politics. New York: Methuen. other worlds: essays in cultural politics. 1987;

57. Le Roux J, Oyedemi TD. Indelible apartheid: intergenerational post-colonial narratives of colonial-born coloured females about hair, race and identity in South Africa. Social Identities. 2022;28(2):150–65.

58. Gerharz E. Indigenous activism in Bangladesh: Translocal spaces and shifting constellations of belonging. Asian Ethnicity. 2014;15(4):552–70.

59. Akter S. Factors influencing health service utilization among mothers for under-five children: A cross-sectional study in Khulna district of Bangladesh. PLoS One. 2022;17(9):e0274449.

60. Navile TE, Hossain J. BIRTH RITUAL, PRACTICES AND GENDER PREFERENCE FOR EXPECTING CHILD IN A MATRILINEAL INDIGENOUS COMMUNITY: A CASE OF GAROS LIVING IN SAINAMARI VILLAGE, MADHUPUR, BANGLADESH. Journal of the Asiatic Society of Bangladesh (Hum). 2019;64(2):239–50.

61. Bhowmik KR, Das S, Islam MA. Modelling the number of antenatal care visits in Bangladesh to determine the risk factors for reduced antenatal care attendance. PLoS One. 2020;15(1):e0228215.

62. Ame AS, Mozumdar L, Islam MA. Impact of social networks on the choice of place of delivery among ethnic women in Bangladesh. Sexual & Reproductive Healthcare. 2021;28:100588.

63. Akter S. Examining access to health services for women of reproductive age from ethnic communities in Chittagong Hill Tracts (CHT) of Bangladesh.

64. Massey DS. Segregation and stratification: A biosocial perspective. Du Bois Rev. 2004;1(1):7–25.

65. Omi M, Winant H. Racial formation rules. Racial formation in the twenty-first century. 2012;302.

66. Bonilla-Silva E. Racism without racists: Color-blind racism and the persistence of racial inequality in the United States. Rowman & Littlefield Publishers; 2006.

67. Jones CP, Truman BI, Elam-Evans LD, Jones CA, Jones CY, Jiles R, et al. Using “socially assigned race” to probe white advantages in health status. Ethn Dis. 2008;18(4):496–504.

68. Janevic T, Piverger N, Afzal O, Howell EA. "Just because you have ears doesn't mean you can hear"—perception of racial-ethnic discrimination during childbirth. Ethn Dis. 2020;30(4):533.

69. Calabrese SK, Meyer IH, Overstreet NM, Haile R, Hansen NB. Exploring discrimination and mental health disparities faced by Black sexual minority women using a minority stress framework. Psychol Women Q. 2015;39(3):287–304.

70. Batist J. An intersectional analysis of maternal mortality in Sub-Saharan Africa: a human rights issue. J Glob Health. 2019;9(1).

71. Hoang TMH, Wong A. Exploring the Application of Intersectionality as a Path toward Equity in Perinatal Health: A Scoping Review. Int J Environ Res Public Health. 2022;20(1):685.

72. Grollman EA. JOURNAL of HEALTH and SOCIAL BEHAVIOR. J Health Soc Behav. 2014;55(1).

73. Karim MA, Kabir MM, Siddiqui MA, Laskar MSI, Saha A, Naher S. Epidemiology of Imported Malaria in Netrokona District of Bangladesh 2013-2018: Analysis of Surveillance Data. Malar Res Treat. 2019;2019.

74. Rahman SMB, Uddin MB, Hussain I. Anthropometric study on children of Garo and non-Garo families in Netrakona district of Bangladesh. Journal of the Bangladesh Agricultural University. 2011;9(2):267–72.

75. Rahman M. Paradoxes of participation of the women leaders in the union parishad in Bangladesh: A phenomenological analysis. Jurnalul Practicilor Comunitare Pozitive. 2019;19(2):58–73.

76. Liamputtong P. Performing qualitative cross-cultural research. Cambridge University Press; 2010.

77. Sandelowski M. Whatever happened to qualitative description? Res Nurs Health. 2000;23(4):334–40.

78. Sandelowski M. Whatever happened to qualitative description? Res Nurs Health. 2000;23(4):334–40.

79. Mayan MJ. Essentials of qualitative inquiry. Routledge; 2016.

80. Sandelowski M. Whatever happened to qualitative description? Res Nurs Health. 2000;23(4):334–40.

81. Caelli K, Ray L, Mill J. 'Clear as mud': toward greater clarity in generic qualitative research. Int J Qual Methods. 2003;2(2):1–13.

82. Akter S, Davies K, Rich JL, Inder KJ. Barriers to accessing maternal health care services in the Chittagong Hill Tracts, Bangladesh: A qualitative descriptive study of Indigenous women's experiences. PLoS One. 2020;15(8):e0237002.

83. Merz S, Jaehn P, Pischon T, Fischer B, Wirkner K, Rach S. Investigating people ' s attitudes towards participating in longitudinal health research : an intersectionality - informed perspective. 2023;1–18.

84. Klar S, Leeper TJ. Identities and intersectionality: a case for Purposive sampling in Survey-Experimental research. Experimental methods in survey research: Techniques that combine random sampling with random assignment. 2019;419–33.

85. Etikan I, Alkassim R, Abubakar S. Comparision of snowball sampling and sequential sampling technique. Biom Biostat Int J. 2016;3(1):55.

86. Atkinson R, Flint J. Accessing hidden and hard-to-reach populations: Snowball research strategies. Social research update. 2001;33(1):1–4.

87. Cochran PAL, Marshall CA, Garcia-Downing C, Kendall E, Cook D, McCubbin L, et al. Indigenous ways of knowing: Implications for participatory research and community. Am J Public Health. 2008;98(1):22–7.

88. Milne J, Oberle K. Enhancing rigor in qualitative description. Journal of Wound Ostomy & Continence Nursing. 2005;32(6):413–20.

89. Braun V, Clarke V. Using thematic analysis in psychology. Qual Res Psychol. 2006;3(2):77–101.

90. Chowdhury SK. Uptake of antepartum care services in a matrilineal-matrilocal society: a study of Garo indigenous women in Bangladesh. BMC Pregnancy Childbirth. 2023;23(1):75.

Dear esteemed reviewer,

I would like to express my sincere gratitude for your invaluable feedback on my manuscript. Your insights and recommendations have been immensely helpful in refining the quality and clarity of the research.

I am pleased to inform you that I have diligently addressed each of your suggestions, and the changes have been incorporated into the revised manuscript. To provide a clear overview of the modifications, I have compiled them in the table below:

Response to the Reviewers Recommendations

**Acknowledgement**<br>I strongly suggest you include at least in this section the acknowledgment of the Garo research assistants, whom not only participated in data collection, but also in the analysis and member checking strategy. Do the same with the teacher.	Dear respected reviewer, thank you for your thoughtful suggestion. In response to your recommendation, I have included acknowledgments for both the Garo research assistants and the teacher in the appropriate section of the manuscript. Their significant contributions to data collection, analysis, and member checking have been duly noted. Your feedback is greatly appreciated.
**Study procedure**<br>• Review this phrase: "This study utilized a qualitative descriptive approach, commonly employed in semi-structured interviews". Do you mean "qualitative descriptive approach, recollecting data with semi-structured interviews"?<br>• Author should expand the description of the method`s scope and utility, explained by Sandelowski: it aims to explore and describe a situation, generally understudied, as it is lived and reported by the people whom experience it. Remember that this approach has a specific scope. It is exploratory and it generally does not have evidence deep enough to support recommendations (as policies). Researchers discuss and conclude based on the data, However, they may point central phenomena to take into consideration in further studies or policy making.<br>• **Sampling:** review the sentence " The participants were categorized into these three groups based on… " ¿which groups?.<br>• **Sampling. Table 1.**<br>This table in qualitative descriptive studies is important as it shows the variation of the sampling, highly valued	1. Dear respected reviewer, thank you very much for your valuable comment. I revised the sentences as: This research was guided by the qualitative description approach. Qualitative description is widely used methodological approach, especially within practice disciplines.<br>2. Dear respected reviewer, I addressed this comment following your guidelines. [please see the 'procedures' section of the revised manuscript.<br>3. Dear respected reviewer, thank you for your feedback. The sentence you mentioned has been revised as follows: "The author categorized their household income into three classes: high, middle, and low, based on their monthly household income in comparison to the national average of monthly household income, which was 15,988 BDT as per Bangladesh Bureau of Statistics (BBS) data."<br>I believe this revision provides a clearer explanation of how the participants were categorized based on their income levels. [please see the result section, page 11]<br>4. Dear respected reviewer, thank you very much for your valuable comment. I have addressed your valuable comments by making the following revisions:

in this kind of research. Variation in the participant´s group assures an expanded exploration of an understudied phenomena. Participant table could be helpful to indicate possible areas or populations that require further research. For this, we find this table in the results section.<br>• Include in this section (sampling) only table 2, adding all the inclusion criteria.<br>• As author have made significant improvements in results and discussions highlighting intersectionality relevance, table 1 is more useful in the first part of the results. This table must be clarified as follows.<br><br>-Please revise precise data of every participant, as in the current table we have mixed information.<br>-Briefly explain how Household economic status is determined in your context.<br>-Clarify what "class five" means in your context ¿basic education or high school?<br>-Briefly explain what does "Self-educated" mean in Garo women context.<br>-Pregnancy diversity can be better understood if it is shared how many children have been born and if they are or not currently pregnant.	• I have relocated Table 1 to the first part of the results section, as suggested by the reviewer. This adjustment helps emphasize the variation in participant groups and the potential areas for further research, aligning with the goals of qualitative descriptive studies.<br>• In response to your recommendation, I have included Table 2 in the sampling section, incorporating all the inclusion criteria. This ensures that the sampling section comprehensively presents participant characteristics.<br><br>- I have revised and provided precise data for each participant, ensuring that the information in the table is clear and well-organized.<br><br>-I have included a brief explanation of how household economic status is determined in the context of the study.<br><br>-I clarified that "class five" in the context refers to primary level of education.<br><br>-I have replaced the term "Self-educated" to illiterate as it will be easier to understand for the reader.<br><br>-I have included information about the number of children each participant has and their pregnancy status to enhance the understanding of pregnancy diversity.
**Analysis**<br>1. In this new version author reports doing content analysis. Even though this approach is highly used in qualitative descriptive analysis, results are presented in themes. There is a common confusion between content and thematic analysis in qualitative descriptive analysis and needs to be avoided. Vaismoradi, Turunen and Bonda clarify how to address this risk in their paper. Please adjust analysis, briefly highlining the process that makes it content analysis or thematic analysis, to	**Analysis**<br>1. Dear respected reviewer, I appreciate your valuable feedback, and I have made the necessary revisions to the analysis section to ensure methodological coherence and transparency. In the revised version, I have explicitly mentioned that I used 'thematic analysis' as the method for analyzing the data. This adjustment helps clarify the approach taken in the study, aligning it with the methodological choice and addressing any potential confusion between content and thematic analysis.

address methodological coherence and transparency in the report.<br>2. Also, clarify if it is a deductive or inductive process. Apparently, the author did a deductive analysis, based on Jones' racism framework, combined with an inductive analysis, as there seem to have emergent categories (not addressed in Jones´ racism theory): Agency; Cultural resistance practice; Community practices of maternal healthcare (traditional medicine practices of maternal health?).	Thank you for highlighting this important point, and I hope the clarification improves the clarity of the report.<br>2. Dear respected reviewer,<br>Thank you for your insightful comments. I have addressed the issue of deductive and inductive analysis in the revised 'Analysis' section of the manuscript. I explicitly mention that I employed both inductive and deductive approaches in a complementary manner.<br>I clarified the rationale behind this choice, explaining that while I initially used a deductive approach based on Jones' racism framework, I also allowed for the emergence of additional categories that were not addressed in Jones' theory. This combination of deductive and inductive analysis enriches the understanding of the data and ensures that both the pre-defined and emergent themes are thoroughly explored.<br>I hope these clarifications provide a more comprehensive view of the analysis process in the study. Thank you for your valuable feedback, which has contributed to improving the rigor and transparency of the research.
**Trustworthiness:**<br>consider to include this section in the procedure as you develop multiple strategies to enhance rigor in your context: triangulation between assistants and researcher during analysis; member-checking of results with participants. Also include here positionality of author and the strategies used to enhance cultural sensitivity with the support of Garo women assistants and the community teacher.	Dear respected reviewer,<br>Thank you for your suggestion regarding the inclusion of a 'Trustworthiness' section in the procedure. I appreciate your thoughtful input. I want to inform you that I have indeed included a separate section titled 'Trustworthiness' immediately after the 'Analysis' section in the revised manuscript. In this section, I have addressed multiple strategies employed to enhance the rigor of the study.<br>I also merged the section on 'Author's Positionality' with the section titled 'Research Site' to provide a more comprehensive understanding of the context and the strategies used to enhance cultural sensitivity with the support of Garo women assistants and the community teacher.<br><br>I believe these revisions have strengthened the manuscript, and I hope they align with your expectations regarding the presentation of trustworthiness measures. Your feedback has been instrumental in refining the study, and I am grateful for your guidance.

**Results**<br><br>Author improved significantly the results report. Fragments included about traditional medicine practices, and about women agency and trust in their culture are valuable. However, because of the way results are presented, there is a major theoretical incoherence that could have a simple solution:<br>1. How does agency, Cultural resistance practice. and Community practices of maternal healthcare constitute internalized racism? It is exactly the opposite of internalized racism. If women had internalized racism (as in the Mexican study you cite on discussion), they would prefer to go the hospitals instead of their midwifes, they would not be proud of their culture, they would even believe they deserve bad treatment.<br>2. This emergent theme is crucial to understand and support Garo Culture and Garo women strategies to face racism at bio-medical centers. Protecting Garo Culture and women agency is as necessary as transforming the biomedical healthcare institution, from the theoretical framework reported. I strongly suggest to create an additional theme grouping these sub-themes, related to cultural strength or resistance towards racism.<br>3. Even though the author clearly has the intention to support Garo Women and Garo culture from racism, the model continues to suggest that more dependency on the cultural practices of maternal healthcare is a negative effect of institutional racism and internalized racism. therefore, it suggests cultural strength cause poor access to biomedical maternity care. In this regard consider that:<br>-there is no sufficient data evidence that describe how more dependency on traditional medicine leads to poor access to biomedical maternity care.<br>-Presenting a model suggesting these relations may contribute to enhance institutional racism, if the reader misunderstand that traditional medicine and cultural practices (and not racism directly) are the reason Garo women do not seek bio-medical maternal health care.	Dear respected reviewer,<br><br>I sincerely appreciate your careful examination of the results and your thoughtful suggestions. I have taken your feedback seriously and made the following revisions to address the issues you raised:<br><br>• **Clarification of Internalized Racism:** I understand the concern you raised regarding the terminology and its alignment with the emergent themes. To address this, I have revised the discussion of the emergent theme related to cultural strength and resistance towards racism to ensure that it is consistent with the model and does not suggest that cultural strength causes poor access to biomedical maternity care. Instead, I have emphasized that this cultural strength represents a resistance strategy against institutional and personally-mediated racism. This revision aligns with the Garo women's experiences and perspectives as reported in the study.<br>• **Adjustment of the Model:** I have revised the model to accurately reflect the relations reported by Garo women. The revised model now highlights that poor access to biomedical maternal healthcare is rooted in institutional and personally-mediated racism. It also acknowledges the emergent broad theme “women Agency” as a resistance strategy against these forms of racism. Internalized racism has been removed from the model, as there was no sufficient data evidence to support its inclusion.<br>4. Dear respected reviewer,<br>Thank you for your valuable feedback regarding the language used in describing the hospital's role in the study. I would like to clarify that the issue of exploitation was commonly shared among all the Garo women participants in the study. Their collective experiences highlighted a systemic concern that needed to be addressed.<br>However, I also understand your suggestion to strike a balanced tone when discussing the hospital's role. While the

-The solution would be to adjust the model, identifying the relations reported by Garo women only: a) Poor access to biomedical maternal healthcare is rooted in institutional and interpersonal; b) there does not seem to be reports of internalized racism. If so, remove it from the Figure. c). the emergent theme (related to strong Cultural traditional-medicine practices / women agency) may work as a resistance towards both types of racism.<br>4. Author may want to attenuate the affirmation of the hospital exploiting Garo women, taking into account a) ¿is it an experience of all the women in the study? (if so, author should report the abuse, if not, author could report the experience of some of the women with some of the staff) b) does the author want the hospital to be open to this study suggestions, and not defensive towards the claims?<br>• P. 16: Adjust this sentence: "This illustrates a link to personally-mediated ethnocentrism, as presented in the following sub-section". (Racism?)	experiences were consistent among participants, it is essential to ensure that the language used remains constructive and encourages collaboration and dialogue with healthcare institutions. I have adjusted the text to reflect this approach, acknowledging the shared experiences of exploitation while maintaining a respectful and open stance toward potential changes within the healthcare system.<br><br>These revisions aim to provide a more accurate representation of the study findings and the experiences of Garo women while fostering a constructive dialogue with healthcare institutions.<br><br>I appreciate your feedback, which has contributed to enhancing the clarity and sensitivity of the study's language and approach.<br>• Dear respected reviewer, I have revised the sentence as per your suggestion, replacing "ethnocentrism" with "Racism" in the revised manuscript. Thank you for pointing out this correction.
**Discussion and conclusion:**<br>1. Results do not suggest how to solve the problem (implementing intercultural training programs) and it is not the scope of this kind of study. How ever, author can attenuate the affirmation, focusing on concluding facts that the study shows based on the evidence of the reports of Garo women: there is strong racism at different levels, and it strongly compromises biomedical healthcare access. Garo women count on a strong traditional medicine system to attend maternal healthcare. Garo women agency derives in their decision to avoid hospitals were they and their culture is mis-respected. Therefore, to increase biomedical healthcare access for Garo women racism needs to be prevent in the institutional and relational level. Author then can suggests the models to explore for future policy-making.<br>2. In discussion and abstract the author refers to Inclusive approaches like	1. Dear respected reviewer,<br>I want to express my sincere gratitude for your insightful comments and recommendations. Your feedback has been instrumental in refining the manuscript, and I truly appreciate your time and expertise. I have incorporated your suggestions into the discussion section to provide a more nuanced and evidence-based conclusion.<br>In light of your input, I have adjusted the discussion as follows:<br>Based on the evidence from the reports of Garo women, it is evident that racism exists at different levels and significantly compromises their access to biomedical healthcare. Garo women have a strong reliance on traditional medicine for maternal healthcare. Their agency is rooted in their decision to avoid hospitals where they and their culture are not respected. Therefore, to enhance biomedical healthcare access for Garo women, it is imperative to address racism

‘Community Controlled Health Services’ and ‘Garo liaison officers’. These programs have not been previously presented, so the relation with result is unclear. Consider to present as examples, or expand it in the introduction.	at both the institutional and relational levels. “then I suggest for implementing intercultural competency training program (CICTP) for the clinicians”.<br><br>2. Dear respected reviewer,<br>Thank you for your comment regarding the mention of 'Community Controlled Health Services' and 'Garo liaison officers' in the discussion and abstract sections of the manuscript. I appreciate your feedback and have made the necessary revisions based on your suggestion.<br>I have removed the policy recommendations related to these inclusive approaches from the discussion section as they were not adequately introduced or connected to the study's findings. Instead, I have focused on highlighting the broader implications of the study's results and the need for addressing racism at different levels within healthcare institutions, as per your earlier recommendations. This change was made to ensure clarity and relevance in the discussion section.<br>Once again, I want to express my gratitude for your valuable input, which has contributed to improving the coherence and effectiveness of the manuscript.
**Other minor revisions:**<br>Here are some fragments that need writing revisions, but a final review of the writing is required.<br>• Revise last phrase on p.4 “It is argues that a consideration of the women's varied social contexts and diverse identities is essential in comprehending their maternity care experiences (44).”<br>• Revise redaction of Jones’s ‘three levels of racism’ new paragraph in introduction.<br>• P. 5. Revise this redaction: “Given such strength, Jones’s theory, evident in studies like Janevic’s on Romani women(43) and others on Indigenous women (3,39,64), highlights racism's impact on maternal healthcare…” and “The Upazila hospital has medical staff, including non-Garo male	• These studies often lack a context-specific understanding of intersectionality and overlook the perspectives of Indigenous women who face compounded prejudice and implicit bias due to various aspects of their identity such as gender and economic class. To comprehensively grasp Indigenous women’s maternity care experiences, it is imperative to consider their diverse identities within distinct social contexts.<br>• The study employs Jones’s ‘three levels of racism' framework (22) along with Crenshaw’s intersectional lens (19) to frame and analyze the themes emerged from participants’ discussion. Jones’s model categorizes racism into three levels: institutional, personally-mediated, and internalized (22). This breakdown helps

gynecologist, with most of the pregnant patients seeking care there are not Garo women".<br>• Positionality: Consider including this section with no title, but merged with the setting or in the suggested section of trustworthiness. In this phrase "The author has a distinct positionality concerning the research site and the participants" what do you mean by distinct positionality?<br>• p.9 This process enhanced trust and credibility between research assistants and participants during data collection, with the research assistants reporting to the author.<br>• P. 14. "As another participant shared, the issue of culturally inappropriate care extends to the midwifery meeting". Do you mean "to the participation of the midwife "?<br>• P14. This explanation may be removed as it is contains in previous paragraph: "This participant expresses concern about the absence of Garo female doctors, as seeking care from a non-Garo male doctor goes against their cultural norms of not sharing personal health problems with male doctors".<br>• P.16. Differentiate researchers' voice from participants: "Distance cost isn't all. Hospitals want money. Despite their claim, they don't charge 5 Taka (Bangladeshi currency). They demand more. Traveling makes you hungry, but food is expensive. We must spend all we earn if we go. She also adds, we don't control travel and treatment costs. They ignore our issue. They said 'you have to follow the hospital rules' whenever we told them our problem. What else can we do but avoid it? (L2, Middle-class, Age-30).<br>• P.21. Consider removing this part of the fragment that does not add much to what is being shown: "I don't know. Probably would have sent me to a free hospital maybe" (L7, Low-class, Age-27).	providing a deeper understanding of the complex impact of racism on maternal healthcare access. Unlike models that primarily focus on the social construction of race or ethnicity and the structural factors perpetuating racial inequalities (64–66), Jones's comprehensive approach offers a holistic understanding. It suggests that institutional and personally-mediated racism contribute to the internalization of racism within the context of maternal healthcare access (22,28). Given its strong theoretical foundation, Jones's model is particularly relevant for our study.<br>• Studies such as Janevic's research on Romani women (45) and similar work with Indigenous women (3,31,67,68), provide significant insights into the influence of racism on maternal healthcare access. However, these studies often neglect a crucial aspect: the intersection of racism with various identities, including gender and economic class. Despite its importance, the impact of this intersectionality on maternal healthcare access is frequently underemphasized (19,69). Maternal health research (70,71) underscores the significance of intersectionality, as it explores how social identities intersect to shape experiences, exacerbate healthcare disparities, and reveal interconnected systems of oppression (19). Hence, in alignment with Jones's 'Three Levels of Racism' theory (22), the current study utilized intersectionality (19) as an analytical framework. It facilitates a clear understanding of how racism intersects with gender and economic class, leading to diverse experiences of discrimination that influence maternal healthcare access (19,69,72). This approach enhances the comprehension of healthcare inequalities and provides valuable insights for addressing the impact of racism on Garo Indigenous women's access to maternal healthcare.<br>• I merge the 'positionality section' with setting.<br>• This strengthened trust and credibility between the research assistants and participants.

	• Dear respected reviewer, I do not mean "to the participation of the midwife ". I revised the sentences as follow to ensure clarity: Another participant also mentioned that culturally inappropriate care includes not being able to meet the birth attendant before delivering in a hospital, which goes against their cultural norms and expectations.<br>• Dear respected reviewer, I removed this sentence: "This participant expresses concern about the absence of Garo female doctors, as seeking care from a non-Garo male doctor goes against their cultural norms of not sharing personal health problems with male doctors".<br>• Dear respected reviewer, in the revised manuscript I tried by best to differentiate author's voice from the participants.<br>• Dear respected reviewer, I removed this fragmented quote: "I don't know. Probably would have sent me to a free hospital maybe" (L7, Low-class, Age-27).

I trust that these alterations have enhanced the overall quality and coherence of the manuscript, aligning it more closely with the standards of the journal.

Once again, thank you for your time, expertise, and commitment to improving the research. Your feedback has been instrumental in strengthening this work.

Kind Regards.

The Author
