# Supplementary material for: Racism and access to maternal health care among garo indigenous women in Bangladesh: A qualitative descriptive study
Source: PLoS One. 2023 Nov 30;18(11):e0294710. doi: 10.1371/journal.pone.0294710 (PMC10688635; doi:10.1371/journal.pone.0294710)
Supplement: Attachment — Submitted filename: Response to reviewers.docx [file pone.0294710.s003.docx]

**Dear Respected Reviewer(s),**

Thank you very much for your kind and valuable comments and guidelines to revise the manuscript. The table below provides the list of your comments and how I addressed this.

| **Abstract**   a. clarify if you used grounded theory or inductive thematic analysis. It looks to me that you used the latter. b. You did not even mention the three levels of ethnocentrism in the results section of your abstract. c. The conclusions in the abstract refer to two levels of ethnocentrism and mention a policy document that you did not mention before. Please, rewrite this section. d. In academic English, the use of long sentences is highly discouraged. Long sentences reduce the readability of texts. Please, revise the English accordingly. | a. The revised one indicates the use of Janevic’s conceptual framework to create analytical categories.[BACKGROUND SECTION, Lines in red color] b. I revised the result section mentioning the three levels of ethnocentrism and their dimensions derived from the data. c. I rewrite the conclusion section. d. I tried my best to avoid using long sentences in the revised manuscript. |
| --- | --- |
| **Background**   a. You say your objective was to study disparities. However, you did not present any comparison with other groups. It looks like your study aimed to address the experience of Garo women in their access to maternal care. Please, revise. b. Revise the paragraph with the definition of ethnocentrism. It is not clear. c. The last paragraph is unnecessary. Please delete | a. Revised the objective of this manuscript to “exploing the how ethnocentrism influence Garo women’s experience of accessing biomedical maternity care” [lines in red color, paraphaph 3 of the BACKGROUND section] [also revised it accordingly in the abstract section, line in red color of the background part] b. I revised the meaning of ethnocentrism with proper citation, first at the begining of the BACKGROUND section [page 3, first line of paragraph 1, (highlighted in red color] and then in the last paragraph of the same section [page 5, lines in red color] c. I have deleted the last paragraph of the ‘Background section’ that presentd the structure of this manuscript. |
| **Methods**   a. What is the 'intersectionality-informed method' you used? Please, explain it briefly when you mention it. b. Table 1 belongs to the results section. In the results, you should start by describing the participants. Also, Tables 1 and 2 could be integrated into one table. c. better explain what are ‘Lengura and Nazirpur Unions of Kalmakanda Upazila.’ Remember, we have an international audience not familiar with Bangladesh. d. You indicated Janevic's conceptual framework (methods) and then Anderson’s framework (in the Discussion) were used to create analytical categories. However, you described an inductive analysis. Please, be more concrete and specific in describing what you did. | A. I explained the meaning of ‘intersectionality informed method’ in the METHOD section of the manuscript with proper citation. Also, mentioned why I used this approach. [lines in red color of the METHOD SECTION, [sub-heading: Sampling and recruitment. Page-7, lines in red color] B. I put the table in the result section. As I removed data on KII respondents considering the word limit and focused solely on the women’s perspective, I deleted table 2. C. I provide some informaion about the Lengura Union and Nazirpur Union [lines in red color, page-5] and also mention where these two Unions are located. I also, include a Map for clear geographical locational indication. [page 6]. D. I mentioned about the use of Janevic’s conceptual framework to create analytical categories, in the revised manuscript. |
| Results   a. You mention that Figure 1 has five domains ‘for more transparent comprehension.’ However, in Figure 1, it seems like you used three categories to summarise results. I. Overall, you present interesting quotes, but they have a lot of repetition. Please, consider making some quotes shorter to facilitate readability. II. What does Ethnocultural mean? Perhaps use cultural. III. What does Womanization mean? Please, consider not using this word if you could explain the idea with more accessible language. IV. Page 11, last paragraph. You said ‘Garo women's acceptance of the health practitioners' ethnic and occupational superiority may be the outcome of internalizing discriminatory attitudes encountered by the former group.’ This is an assumption. Please delete. V. Last sentence on page 13. Interestingly, while showing the ethnocentric aspects of maternal care, the author reproduces these views, for example, when describing mothers and mothers-in-law as ignorant people who cannot provide proper advice to pregnant women. Is this assumption supported by data? It is unacceptable that you describe Garo mothers as ignorant and discuss the cultural competency of their care simultaneously. Furthermore, this topic should be part of the health literacy part. VI. Clarify the first sentence of Health System Factors. Also, it is unclear if you are addressing health system-level issues here because the content is mostly about institutional care. VII. Please, avoid repetition. For example, the information in the last paragraph of page 15 was already mentioned on page 9 as part of a different category. VIII. The last paragraph of the results belongs to the discussion. Please delete. | a. In the revised manuscript, I revised the Figure 1 with some new dimensions. Also, revised the part second paragraph of the RESULT section that illustrates the Figure 1. [lines in red color] I. I tried my best to revised the result section following the revised Figure 1 and remored all the repetitions. II. I replaced the ‘ethno-cultural’ to culture. III. In the revised manuscript I did not use the work ‘womanization’.      IV. I deleted this paragraph. V. I deteled this paragraph. VI. I deleted this sentence. VII. I deleted the discussion on health system factor and focused on institutional, personally-mediated, and internalized ethnocentrism in the revised manuscript. VIII. I deleted the last paragraph. |
| **Discussion**   a. Why did you mention Anderson’s framework? This seems very unnecessary. Perhaps it is better to delete it. b. How did you jump from three categories of ethnocentrism to five types of discrimination? Please, avoid this or explain the methods and present the results. c. **References 39-41 have nothing to do with the Upazila. They do not support your statement.** d. You should write a shorter discussion section which addresses the following topics (do not use these as subheadings): a statement of the principal findings; strengths and weaknesses of the study; strengths and weaknesses in relation to other studies, discussing important differences in results; the meaning of the study: possible explanations and implications for clinicians and policymakers; and unanswered questions and future research. | a. I deleted it. b. In the revised manuscript I kept my focus on the three categories of ethnocentrism and the themes emerged from the data, avoiding external issues not conforming to the Figure 1. c. I deleted this references in the revised DISCUSSION Section of the manuscript. d. In the revised discussion, I tried my best to answer these questions. |

Kind Regards.

The Author.
