# Supplementary material for: Racism and access to maternal health care among garo indigenous women in Bangladesh: A qualitative descriptive study
Source: PLoS One. 2023 Nov 30;18(11):e0294710. doi: 10.1371/journal.pone.0294710 (PMC10688635; doi:10.1371/journal.pone.0294710)
Supplement: Attachment — Submitted filename: PLOS 1.docx [file pone.0294710.s004.docx]

**REVIEW REPORT FOR PLOS ONE JOURNAL**

**NAME OF JOURNAL**: PLOS ONE

**ARTICLE TYPE**: RESEARCH ARTICLE

**MANUSCRIPT NUMBER**: PONE-D-22-34953R1

**ARTICLE TITLE**: **They don’t even listen to our voices”: Ethnocentrism and Biomedical Maternity Care Access Among Garo Indigenous Women in Bangladesh.**

**Date: 28.05.2023**

GENERAL COMMENTS

I want to thank you for allowing me to review this manuscript.

*I don’t recommend this paper for publication in its current form. The data is good and can be presented in another form, with a different title. Something like “GARO WOMEN CALLS FOR HELP WITH MATERNAL HEALTH SERVICES’…*

*The manuscript did have line numbering, so it was difficult referring the specific lines in each paragraph. I made comments on the manuscript with highlights.*

Generally, the manuscript has gaps though it was presented in simple language.

1. TOPIC: The topic is very interesting and contains complex issues. The topic reveals the Garo women’s maternal health issues that looks like the story is half full. As a reviewer, I think there is more to it than has been presented. There is more of a power struggle than has been presented in the manuscript. From the details of the manuscript, it is not just the healthcare workers not listening to the women’s voices, but … the Garo culture for instance is in the middle of all these issues. It is complicated and racism has a major role in there. It looks like the Garo women are a minority group that needs protection from the very top (government, civil society, NGO’s etc). Having been familiar with Gender issues, I do not want to be associated with sparking gender-related conflicts, tribal and racism.
2. **TITLE**: The title is **not** appropriate, though catchy. However, considering the whole manuscript, some documentation is unhealthy. For example, a situation where the only Gynaecologist was labelled to be a non-Garo man, and this was given as a reason he did not attend to Garo women. In qualitative, we don’t the issue of confidentiality is non-negotiable. Non-exposure of people (either the participants or those they are dealing with).
3. **ABSTRACT:** The abstract is well presented
4. The aim/purpose/research objectives are clearly stated.
5. Yes, pertinent previous work has been cited.
6. Discussion to be redone for clarity.
7. Clinical implications were missing.
8. The quality of the science could be better.
9. Table: not well structured.

**SPECIFIC COMMENTS**

BACKGROUND

The background provided relevant and adequate information on the subject matter.

METHODS

No research design is described under the methods, though a sentence about the design is mentioned in the abstract.

No detailed description of the data analysis was documented.

In short, the research method needs to be redone for clarity.

Details of confidentiality not addressed.

RESULTS

1. Results not well sufficiently presented. The three main groupings of the results should be sufficiently presented with distinctions and different dimensions of the quotes from the participants.
2. The presentation of the results was mixed up, the quotes of the participants should march the narratives. For example, on page 10, a participant quote reads … ‘

“The hospital has no services, making pregnancy care impossible. We have trouble getting care because the only available doctor always found busy with non-Garo women. The man gaini doctor (gynecologist) always serves non-Garo women, ignoring us. What can I do there? I don't go to hospitals like my neighbors” (L8, 26).

This other one;

Distance cost isn't all. Hospitals want money. Despite their claim, they don't charge 5 BDT. They demand more. Traveling makes you hungry, but food is expensive. We must spend all we earn if we go. She also adds, we don't control travel and treatment costs. They ignore our issue. They said "you 11 have to follow the hospital rules" whenever we told them our problem. What else can we do but avoid it? (L2, age 30).

“You (author) need to read through the quotes very well and do a proper presentation of data. There is service, but not for Garo women”.

3. It looks like there is a brief discussion and conclusion in the presentation of the results.

**DISCUSSION**

Just after the introductory paragraph, the methodological limitations of the study were indicated. The discussion should follow the pattern of the result presentation and be chronological.

**CONCLUSION**

The conclusion suggested training with unclear statements as follows;

“The study suggests that CICTP training programs and institutional initiatives related to increasing the number of hospital staffing and 19 providing culturally competent care are strikingly necessary. Such training does not substitute any existing training program organized for the clinicians, but to inject a strong sense of ethnicrelations to ensuring the delivery of culturally appropriate care acknowledging the ethnic diversity.”

I think all that is needed is inclusivity, equity and well as diversity and be treated as a matter of agency. It should be seen as everybody’s business.

**IMPLICATION OF THE STUDY**

No implications of the study.

Thank you very much.
