# Supplementary material for: Racism and access to maternal health care among garo indigenous women in Bangladesh: A qualitative descriptive study
Source: PLoS One. 2023 Nov 30;18(11):e0294710. doi: 10.1371/journal.pone.0294710 (PMC10688635; doi:10.1371/journal.pone.0294710)
Supplement: Attachment — Submitted filename: PONE-D-22-34953R2_Review.docx [file pone.0294710.s007.docx]

The author made significant effort improving the report of this pertinent study. There is still work to do, particularly in results and discussion. Considering the importance of sharing findings related to maternity healthcare scenario of Garo women as a risk for them and their culture, I encourage the author to consider the following recommendations.

**Acknowledgement**

I strongly suggest you include at least in this section the acknowledgment of the Garo research assistants, whom not only participated in data collection, but also in the analysis and member checking strategy. Do the same with the teacher.

**Study procedure**

1. Review this phrase: “This study utilized a qualitative descriptive approach, commonly employed in semi-structured interviews”. Do you mean “qualitative descriptive approach, recollecting data with semi-structured interviews”?
2. Author should expand the description of the method`s scope and utility, explained by Sandelowski: it aims to explore and describe a situation, generally understudied, as it is lived and reported by the people whom experience it. Remember that this approach has a specific scope. It is exploratory and it generally does not have evidence deep enough to support recommendations (as policies). Researchers discuss and conclude based on the data, However, they may point central phenomena to take into consideration in further studies or policy making.
3. **Sampling**: review the sentence “ The participants were categorized into these three groups based on… “ ¿which groups?.
4. **Sampling. Table 1.**

This table in qualitative descriptive studies is important as it shows the variation of the sampling, highly valued in this kind of research. Variation in the participant´s group assures an expanded exploration of an understudied phenomena. Participant table could be helpful to indicate possible areas or populations that require further research. For this, we find this table in the results section.

1. Include in this section (sampling) only table 2, adding all the inclusion criteria.
2. As author have made significant improvements in results and discussions highlighting intersectionality relevance, table 1 is more useful in the first part of the results. This table must be clarified as follows.

| Place of residence | Participant | Age range  (particular individual age, or avoid the column) | Household economic Status | Educational Level | Pregnancy diversity  (pregnancy status?) |
| --- | --- | --- | --- | --- | --- |
| Lengura Union | L1 |  |  |  |  |
|  | L2.. |  |  |  |  |
| Nazirpur U | N1 |  |  |  |  |
|  | N2 |  |  |  |  |

- Please revise precise data of every participant, as in the current table we have mixed information.
- Briefly explain how Household economic status is determined in your context.
- Clarify what “class five” means in your context ¿basic education or high school?
- Briefly explain what does “Self-educated” mean in Garo women context.
- Pregnancy diversity can be better understood if it is shared how many children have been born and if they are or not currently pregnant.

**Analysis**

1. In this new version author reports doing content analysis. Even though this approach is highly used in qualitative descriptive analysis, results are presented in themes. There is a common confusion between content and thematic analysis in qualitative descriptive analysis and needs to be avoided. [Vaismoradi, Turunen and Bonda](https://pubmed.ncbi.nlm.nih.gov/23480423/) clarify how to address this risk in their paper. Please adjust analysis, briefly highlining the process that makes it content analysis or thematic analysis, to address methodological coherence and transparency in the report.
2. Also, clarify if it is a deductive or inductive process. Apparently, the author did a deductive analysis, based on Jones’ racism framework, combined with an inductive analysis, as there seem to have emergent categories (not addressed in Jones´ racism theory): Agency; Cultural resistance practice; Community practices of maternal healthcare (traditional medicine practices of maternal health?).

**Trustworthiness**:

consider to include this section in the procedure as you develop multiple strategies to enhance rigor in your context: triangulation between assistants and researcher during analysis; member-checking of results with participants. Also include here positionality of author and the strategies used to enhance cultural sensitivity with the support of Garo women assistants and the community teacher.

**Results**

Author improved significantly the results report. Fragments included about traditional medicine practices, and about women agency and trust in their culture are valuable. However, because of the way results are presented, there is a major theoretical incoherence that could have a simple solution:

1. How does agency, Cultural resistance practice. and Community practices of maternal healthcare constitute internalized racism? It is exactly the opposite of internalized racism. If women had internalized racism (as in the Mexican study you cite on discussion), they would prefer to go the hospitals instead of their midwifes, they would not be proud of their culture, they would even believe they deserve bad treatment.
2. This emergent theme is crucial to understand and support Garo Culture and Garo women strategies to face racism at bio-medical centers. Protecting Garo Culture and women agency is as necessary as transforming the biomedical healthcare institution, from the theoretical framework reported. I strongly suggest to create an additional theme grouping these sub-themes, related to cultural strength or resistance towards racism.
3. Even though the author clearly has the intention to support Garo Women and Garo culture from racism, the model continues to suggest that more dependency on the cultural practices of maternal healthcare is a negative effect of institutional racism and internalized racism. therefore, it suggests cultural strength cause poor access to biomedical maternity care. In this regard consider that:

- there is no sufficient data evidence that describe how more dependency on traditional medicine leads to poor access to biomedical maternity care.
- Presenting a model suggesting these relations may contribute to enhance institutional racism, if the reader misunderstand that traditional medicine and cultural practices (and not racism directly) are the reason Garo women do not seek bio-medical maternal health care.
- The solution would be to adjust the model, identifying the relations reported by Garo women only: a) Poor access to biomedical maternal healthcare is rooted in institutional and interpersonal; b) there does not seem to be reports of internalized racism. If so, remove it from the Figure. c). the emergent theme (related to strong Cultural traditional-medicine practices / women agency) may work as a resistance towards both types of racism.

4. Author may want to attenuate the affirmation of the hospital exploiting Garo women, taking into account a) ¿is it an experience of all the women in the study? (if so, author should report the abuse, if not, author could report the experience of some of the women with some of the staff) b) does the author want the hospital to be open to this study suggestions, and not defensive towards the claims?

P. 16: Adjust this sentence: “This illustrates a link to personally-mediated ethnocentrism, as presented in the following sub-section”. (Racism?)

**Discussion and conclusion:**

1. Results do not suggest how to solve the problem (implementing intercultural training programs) and it is not the scope of this kind of study. How ever, author can attenuate the affirmation, focusing on concluding facts that the study shows based on the evidence of the reports of Garo women: there is strong racism at different levels, and it strongly compromises biomedical healthcare access. Garo women count on a strong traditional medicine system to attend maternal healthcare. Garo women agency derives in their decision to avoid hospitals were they and their culture is mis-respected. Therefore, to increase biomedical healthcare access for Garo women racism needs to be prevent in the institutional and relational level. Author then can suggests the models to explore for future policy-making.
2. In discussion and abstract the author refers to Inclusive approaches like ‘Community Controlled Health Services’ and ‘Garo liaison officers’. These programs have not been previously presented, so the relation with result is unclear. Consider to present as examples, or expand it in the introduction.

**Other minor revisions:**

Here are some fragments that need writing revisions, but a final review of the writing is required.

- Revise las phrase on p.4 “It is argues that a consideration of the women's varied social contexts and diverse identities is essential in comprehending their maternity care experiences (44).”
- Revise redaction of Jones’s ‘three levels of racism’ new paragraph in introduction.
- P. 5. Revise this redaction: “Given such strength, Jones’s theory, evident in studies like Janevic’s on Romani women(43) and others on Indigenous women (3,39,64), highlights racism's impact on maternal healthcare…” and “The Upazila hospital has medical staff, including non-Garo male gynecologist, with most of the pregnant patients seeking care there are not Garo women”.
- Positionality: Consider including this section with no title, but merged with the setting or in the suggested section of trustworthiness. In this phrase “The author has a distinct positionality concerning the research site and the participants” what do you mean by distinct positionality?
- p.9 This process enhanced trust and credibility between research assistants and participants during data collection, with the research assistants reporting to the author.
- P. 14. “As another participant shared, the issue of culturally inappropriate care extends to the midwifery meeting”. Do you mean “to the participation of the midwife ”?
- P14. This explanation may be removed as it is contains in previous paragraph: “This participant expresses concern about the absence of Garo female doctors, as seeking care from a non-Garo male doctor goes against their cultural norms of not sharing personal health problems with male doctors”.
- P.16. Differentiate researchers’ voice from participants: “Distance cost isn’t all. Hospitals want money. Despite their claim, they don’t charge 5 Taka (Bangladeshi currency). They demand more. Traveling makes you hungry, but food is expensive. We must spend all we earn if we go. She also adds, we don't control travel and treatment costs. They ignore our issue. They said ‘you have to follow the hospital rules’ whenever we told them our problem. What else can we do but avoid it? (L2, Middle-class, Age-30).
- P.21. Consider removing this part of the fragment that does not add much to what is being shown: “I don’t know. Probably would have sent me to a free hospital maybe” (L7, Low-class, Age-27).
