# Supplementary material for: Racism and access to maternal health care among garo indigenous women in Bangladesh: A qualitative descriptive study
Source: PLoS One. 2023 Nov 30;18(11):e0294710. doi: 10.1371/journal.pone.0294710 (PMC10688635; doi:10.1371/journal.pone.0294710)
Supplement: Attachment — Submitted filename: Response to Reviewer Comments.pdf [file pone.0294710.s010.pdf]

Dear Esteemed Reviewer,

I wish to convey my heartfelt appreciation for your invaluable feedback regarding my manuscript. Your insights and recommendations have played a pivotal role in enhancing the quality and clarity of the research. I am delighted to inform you that I have diligently addressed each of your suggestions, and the changes have been seamlessly incorporated into the revised manuscript (please see the lines in red color in the manuscript). Additionally, I have also addressed the revisions you suggested in the manuscript (please see the revision highlighted in green color in the manuscript). To offer a clear and organized overview of the modifications, I have compiled them in the table below:

Response to Reviewer Comments

• Pay attention in the methodological section that the verb ending is uniform in the past tense.	Dear respected Reviewer,<br>I want to express my gratitude for your thorough review and attention to detail. Ensuring consistency in the verb endings in the past tense throughout the methodological section is an essential aspect of maintaining the quality of the paper. I carefully reviewed and revised the section to address this concern, and I appreciate your valuable input in maintaining the linguistic consistency and precision of the content.
• On page 18, the methodological design is qualitative descriptive approach. No qualitative description.	Dear respected Reviewer,<br>Thank you for your meticulous review of the manuscript. I appreciate your keen eye for detail. I will make the necessary correction on page 18, ensuring that the methodological design is referred to as a "qualitative descriptive approach" rather than "qualitative description." Your feedback is essential in maintaining precision and clarity in the content, and I'm grateful for your contribution to the refinement of the paper.
• Trustworthiness (p.10) Author can redact this section, in a simpler way: The strategies to enhance trustworthiness in this study where…	Dear respected Reviewer,<br>I want to express my gratitude for your thoughtful review and suggestions. I agree that the section on trustworthiness (on page 10) can be revised to enhance clarity and simplicity. I made the necessary revisions to present the strategies for ensuring trustworthiness in a more straightforward and reader-friendly manner. Your feedback is highly valuable, and I appreciate your contribution to improving the manuscript. [please see the revised section 'trustworthiness'.
• Ethical considerations (P.22). Replace "privacy" with "anonymity".	Dear respected Reviewer,<br>I want to express my gratitude for your careful review of the manuscript. Your suggestion to replace "privacy" with "anonymity" in the section on ethical considerations (on page 22) is well-noted and appreciated. I agree that the term "anonymity" is more accurate and aligns better with the ethical considerations discussed in that section. I made the recommended change to enhance the precision of the content and ensure it accurately reflects the research ethics.<br>Thank you for your valuable input, which contributes to the overall quality of the paper.

Results: • (P. 27). Correct the capital “P” in Participants.<br>• The last quote of the participants (p.23) is significantly relevant: not only it shows women agency, but also indicates the perceived efficacy of the traditional medicine in women healthcare.<br>• (p. 25) Please briefly clarify the discussion with the Ethiopia study and the other study about identity based racial discrimination at the institutional level. It still not clear how does this study contrast with the study in Ethiopia.	• Dear respected Reviewer,<br>Thank you for your careful review and for bringing to my attention the capitalization issue with "Participants." I apologize for the oversight, and I made the necessary correction to ensure consistency in capitalization throughout the document. Your attention to detail is greatly appreciated, and it helped improve the overall quality of the manuscript.<br>• Dear respected Reviewer,<br>Thank you for your kind and valuable observation. I appreciate your observation and recognition of the significance of the last quote from the participants on page 23.<br>• Dear respected Reviewer,<br>Thank you for your valuable comment. I appreciate your interest in further clarification regarding the contrast between this study and the research in Ethiopia. To provide a more convincing explanation, it’s important to emphasize that in this study conducted in Bangladesh, the primary obstacle for Garo Indigenous women’s maternal healthcare access is institutional racism within healthcare institutions, which stems from Indigenous identity-based discrimination. In contrast, in Ethiopia, the challenges faced by Indigenous women were more closely related to the actual unavailability of healthcare resources, along with cultural norms and a shortage of female healthcare professionals. By underlining these differences, I aim to illustrate how various factors contribute to racial discrimination in distinct regional contexts. **[please see page 25, ‘discussion section’, lines in red color]**<br>Thank you for your understanding and guidance.

Your diligent and thoughtful review has been instrumental in improving the manuscript, and I am deeply grateful for your contribution.

Sincerely,
The Author
