# Supplementary material for: Racism and access to maternal health care among garo indigenous women in Bangladesh: A qualitative descriptive study
Source: PLoS One. 2023 Nov 30;18(11):e0294710. doi: 10.1371/journal.pone.0294710 (PMC10688635; doi:10.1371/journal.pone.0294710)
Supplement: Attachment — Submitted filename: Response to the academic editors and Reviewer Comments.pdf [file pone.0294710.s011.pdf]

Dear Esteemed Academic Editor and Reviewer,
I wish to convey my heartfelt appreciation for your invaluable feedback regarding my manuscript. Both of your insights and recommendations have played a pivotal role in enhancing the quality and clarity of the research. Dear respected academic editor, I am delighted to inform you that I have diligently addressed each of your suggestions, and the changes have been seamlessly incorporated into the revised manuscript (please see the lines in green color in the revised manuscript). Accordingly, dear respected reviewer, I also made my best effort to respond to your valuable comments by making necessary revisions in the manuscript, as indicated by the red-colored lines in the revised document.

To provide a clear and organized overview of the modifications, I have compiled them in the tables below. Table 1 represents the author's response to the academic editor's comments, and Table 2 represents the author's response to the reviewer's comments:

Table 1 Response to the Academic Editor's Comments

Academic Editor's comments	Author's response
Jones'?	Dear respected academic editor, I would like to express my sincere gratitude for your valuable feedback. In light of your insightful comment, I have made the adjustment of using "Jones' " instead of "Jones's." Your input has been immensely helpful, and I truly appreciate your guidance in enhancing the quality of my work.
Reference 8 does not support the claim.	Dear respected academic editor, I would like to extend my heartfelt appreciation for your invaluable comment. In response to your comment, I have incorporated a more pertinent reference (citation 6 in the revised manuscript) in place of the previously cited reference 8. Your guidance has been instrumental in improving the quality of my manuscript, and I am truly thankful for your assistance.
"Virginia Braun and Victoria Clarke's" Use only the last name of the authors	Dear respected academic editor, I want to thank you for your insightful guidance. In accordance with your suggestion, I have revised "Virginia Braun and Victoria Clarke's" to "Braun and Clarke's". Your input has been immensely helpful, and I genuinely value your assistance in refining my manuscript.
"Finally, the author writes the result section using the identified themes and relevant verbatim quotes from the transcripts. A summary of the results, written in Mandi language through collaborative efforts with research assistants, is shared with participants to gather their feedback for the final draft. Once consent is obtained, the author revisits the study area, invites all participants, and presents the final version of the research findings.	Dear respected academic editor, I would like to express my profound gratitude for your invaluable comment. Your feedback has been of immense value. In accordance with your guidance, I have carefully revised the English in the manuscript as follows: Finally, the author wrote the results section using the identified themes and relevant

Shila Gagra and research assistants assist the author in this process. “Revise English.<br>It is important to maintain consistency in the usage of verb tenses.”	verbatim quotes from the transcripts. A summary of the results, written in Mandi language through collaborative efforts with research assistants, was shared with Participants to gather their feedback for the final draft. Once consent was obtained, the author revisited the study area, invited all Participants, and presented the final version of the research findings. Shila Gagra and research assistants assisted the author in this process.
Trustworthiness: The triangulation between assistants and author during analysis; re-checking the codes and themes, member-checking of results with participants which has been abstracted thus far demonstrates the author’s effort to ensure trustworthy of the research findings. “Revise English”	Dear respected academic editor, I wish to convey my deep appreciation for your valuable comment. Your feedback is highly valuable to me. In accordance with your guidance, I have made the necessary revisions to the English in the manuscript as follows: The triangulation involving the collaboration between the author and research assistants during the analysis, the subsequent review of codes and themes, and the member-checking of results with participants, demonstrates the author’s commitment to ensuring the trustworthiness of the research findings.
There was not local ethics. “Revise English”.	Dear respected academic editor I extend my heartfelt appreciation for your valuable comment. Your feedback is of great importance to me. In accordance with your guidance, I have diligently revised the English in the manuscript as follows: There was no local ethics committee from which the author required to seek approval.
Thematic saturation “In the methods you need to explain how did you define thematic saturation.”	Dear respected academic editor, I want to express my deep appreciation for your valuable comment. Your feedback is incredibly important to me. Following your guidance, in the sub-section discussing the participants' characteristics, I have taken the opportunity to provide a clear clarification on how I defined ‘thematic saturation: Thematic saturation achieved after nineteen IDIs, signifying the point where no new themes or insights emerged. To deepen understanding, an additional five IDIs were conducted.
You need to explain what do you mean by exploitation.	Dear respected academic editor, I would like to express my sincere gratitude for your valuable comment. Your feedback is highly

	appreciated. In accordance with your guidance, in the revised manuscript, I have removed the word 'exploitation' and rephrased the sentence as follows: These women consistently mentioned feeling disrespected and unfairly treated by hospital staff, highlighting their sense of mistreatment.
This category is unique in this study. “Please delete this. The discussion of results belongs to another section.”	Dear respected academic editor, I want to express my deep appreciation for your valuable comment. Your feedback is of great importance to me. Following your guidance, I have taken the step of removing the mentioned sentence from the revised manuscript. I have also addressed this topic in the discussion section, as suggested.
Potential financial exploitation. “The use of exploitation in this context is unclear. What do you mean?”	I would like to extend my heartfelt gratitude for your valuable comment. Your feedback holds significant importance to me. In response to your guidance, I have replaced the term 'financial exploitation' with 'financial burden' in the revised manuscript.
Hospital exploitation. “Revise English”.	Dear respected academic editor, I wish to express my deep appreciation for your valuable comment. Your feedback is highly significant to me. In accordance with your guidance, I have made the necessary revisions to the English in the manuscript as follows: This participant’s discussion serves as an illustration of the systemic challenges encountered by Indigenous women in their pursuit of affordable and equitable healthcare.
institutionalized ethnocentrism “You included this concept without an explanation of its meaning. You need to make the connection with it in the methods when you explain the concepts of racism. Another option is not to use it but be consistent with racism.”	Dear respected academic editor, I want to convey my sincere gratitude for your valuable guidance. Your feedback is greatly appreciated. In line with your recommendations, I have replaced the term 'ethnocentrism' with 'racism' in the manuscript.
Overall, the participants typically blamed insufficient human resources, culturally inappropriate care, and the dominant power of the clinicians for poor access. Also, they present the obligation of following the institutional guidelines defined by the clinicians as a reason for not accessing these services. This illustrates a link to personally-mediated racism, as presented in the following sub-section. “This paragraph is unnecessarily repetitive. Please delete it.”	Dear respected academic editor, I wish to express my heartfelt gratitude for your valuable comment. Your feedback is of utmost importance to me. In accordance with your guidance, I have removed the mentioned paragraph from the revised manuscript.

touching private organs. “Revise English”	Dear respected academic editor, I would like to extend my sincere appreciation for your valuable comment. Your feedback is highly regarded. In accordance with your guidance, I have refined the phrasing of 'touching private organs' in the manuscript as follows: They described situations where clinicians used force, performed unconsented vaginal checkups, and prevented families from following their cultural rituals involving placenta. As, they unanimously shared, these actions significantly hindered the delivery of respectful maternal healthcare.
The data shows that clinicians’ preference for C-sections for Garo women reflects institutionalized racism, disregarding the cultural belief in the safety of normal birthing. This interplay between institutional and personally-mediated racism shapes internalized racism among the Garo Indigenous women, as discussed in the following sub-section. “This paragraph is unnecessarily repetitive. Please delete it.”	Dear respected academic editor, I want to express my profound gratitude for your valuable comment. Your feedback is highly appreciated. In line with your guidance, I have taken the step of removing the mentioned paragraph from the revised manuscript.
The sub-themes that emerged this category include, “Revise English”.	Dear respected academic editor, I would like to express my deep gratitude for your valuable comment. Your feedback is of great importance to me. In accordance with your guidance, I have made the necessary revisions to the English in the manuscript as follows: The sub-themes that came up within this category consist of cultural resistance, community practices of maternal healthcare, and perceived worthlessness of biomedical care.
Typically. “Please, do not use typically every time you will introduce a quote.”	Dear respected academic editor, I wish to convey my sincere gratitude for your valuable comment. Your feedback is greatly appreciated. In line with your guidance, I have made the necessary adjustments to avoid the repetitive use of the word 'typically' in the manuscript.
Helps. “Contributes?”	Dear respected academic editor, I want to extend my deep appreciation for your valuable comment. Your feedback is highly regarded. In accordance with your guidance, I have replaced the word 'helps' with 'contributes' in the revised manuscript.
care is value-less. “Revise English”.	Dear respected academic editor, I wish to express my profound gratitude for your valuable comment. Your feedback is highly appreciated. In accordance with your

	guidance, I have made the necessary revisions to the English in the manuscript as follows: Hospital-level care has no value for me because they disrespect us.
decide on an alternative. “Traditional?”	Dear respected academic editor, I would like to convey my deep appreciation for your valuable comment. Your feedback is greatly valued. In accordance with your guidance, I have replaced the term 'alternative' with 'traditional' in the revised manuscript.
the types of their community practices. “Revise English”.	Dear respected academic editor, I want to express my sincere gratitude for your valuable comment. Your feedback is of great importance to me. In accordance with your guidance, I have made the necessary revisions to the English in the manuscript as follows: When asked about the maternal healthcare practices within their community, along with this participant, another woman typically shared:
birth attendants. “Please, use traditional midwives” “Revise English”	Dear respected academic editor, I wish to extend my heartfelt gratitude for your valuable comment. Your feedback is highly appreciated. In line with your guidance, I have employed the term 'traditional midwives' instead of 'birth attendants' in the revised manuscript.
Thus, the participants’ experiences indicate that the internalization of the intersectional mix of institutional and personally-mediated racism by clinicians led to feelings of disempowerment and self-devaluation at hospitals. They perceived biomedical care as less valuable than community practices of maternal healthcare, which they found non-discriminatory. As a result, they sought cultural empowerment and resistance through community-based maternity care to avoid discrimination and stigmatization at hospitals. “Please, do not duplicate the reporting of results. Delete this paragraph.”	Dear respected academic editor, I want to express my profound gratitude for your valuable comment. Your feedback is greatly valued. In accordance with your guidance, I have taken the step of removing the mentioned paragraph from the revised manuscript.<br><br>Your insights have been instrumental in shaping the content, and I genuinely appreciate your assistance.
healthcare, which differs from a previous study. “Revise English. Do you mean this result differs from the results of another study? How is it different?”	Dear respected academic editor, I would like to convey my sincere appreciation for your valuable comment. Your feedback is highly regarded. In the revised manuscript, I have rephrased as follows:<br>The findings indicate that the interplay between two forms of racism contributes to the strengthening of among the Garo women, ultimately shaping their preference for community-based maternal healthcare. This represents a departure from previous

	research conducted by Jenevic et al. [45] on Roma minority women in the Balkans. In their study, Jenevic et al. found that institutional and personally-mediated racism had different effects, leading to the internalization of racism among Roma women. This internalization was linked to the emergence of psychological factors, particularly a decline in self-esteem and self-efficacy.
Citation 33 "This does not seem to be the correct reference"	(citation 3 and 21)<br>• Varcoe C, Brown H, Calam B, Harvey T, Tallio M. Help bring back the celebration of life: A community-based participatory study of rural Aboriginal women's maternity experiences and outcomes. BMC Pregnancy Childbirth. 2013;13(1):1–10. Available from: https://doi.org/10.1186/1471-2393-13-26<br>• Bowser D, Hill K. Exploring evidence for disrespect and abuse in facility-based childbirth: report of a landscape analysis. USAID-Traction project. 2010 Sept 20. Available from: https://www.hsph.harvard.edu/wp-content/uploads/sites/2413/2014/05/Exploring-Evidence-RMC_Bowser_rep_2010.pdf
in Ethiopia, where actual unavailability reduced Indigenous maternal healthcare access, indicating context-specific variations. "This is not clear. Please rephrase."	Dear respected academic editor, I wish to express my deep appreciation for your valuable comment. Your feedback is greatly appreciated. In the revised manuscript, I have rephrased as follows: In contrast, this study diverges from previous research conducted in Ethiopia, where Indigenous women faced difficulties in accessing maternal healthcare primarily due to the actual scarcity of healthcare resources [42]. However, the study involving Garo Indigenous women in Bangladesh underscores that the main obstacle they encounter is institutional racism within healthcare facilities, rooted in their Indigenous identity. This variation underscores how local circumstances play a

	significant role in shaping discrimination across different regions [41].
Financial exploitation. “Please, revise if this is the right concept.”	Dear respected academic editor, I would like to extend my sincere appreciation for your valuable comment. Your feedback is highly regarded. In the revised manuscript, I have incorporated the 'financial burden' concept instead of 'financial exploitation' and have rephrased the sentence as follows: Hidden costs and financial burden are experienced by Garo women from economically underprivileged communities, demonstrating racial and financial bias.
The participants’ stories make clear the ingrained stereotypes and discriminatory practices that Garo Indigenous women must deal with within the healthcare system. “This is not clear.”	Dear respected academic editor, I want to convey my deep appreciation for your valuable comment. Your feedback is of great importance to me. In the revised manuscript, I have rephrased as follows: The stories told by the participants clearly show the long-standing biases and unfair treatment that Garo Indigenous women often experience within the healthcare system.
tendency is also evident. “Revise English”.	Dear respected academic editor, I would like to express my sincere gratitude for your valuable comment. Your feedback is highly appreciated. In response to your guidance, I have revised the English in the revised manuscript.<br><br>Your insights have played a significant role in improving the quality of my work, and I genuinely value your assistance.
Garo women often reinforce each other. “You can relate this with the concept of cultural safety of medical practice. See https://journalhosting.ucalgary.ca/index.php/cmej/article/view/72675”	Dear respected academic editor, I want to extend my sincere gratitude for your valuable suggestion. Your feedback is greatly appreciated. In the revised manuscript, I have established a connection between the experiences of Garo Indigenous women and the concept of cultural safety.<br><br>Your insights have been instrumental in enhancing the content, and I genuinely value your assistance.
Accordingly, an intersectional analysis of the participants’ discussion shows that their experiences, similar to institutional racism, are influenced not only by their Indigenous identity but also by their gender, culture, and socioeconomic level (19). The difficulties and prejudices people confront in the healthcare system are exacerbated by these interconnected identity	Dear respected academic editor, I wish to express my deep appreciation for your valuable comment. Your feedback is highly valued. In accordance with your guidance, I have taken the step of removing the mentioned paragraph from the revised manuscript.

characteristics. The participants' discussions shed light on how personally-mediated racism intertwines with an inter-personal power hierarchy, resulting in ongoing discrimination. Nonsensical medical jargon and discriminatory attitudes toward the Garo language exacerbate their marginalization. "This paragraph does not discuss findings. Consider deleting it."	Your insights have been instrumental in shaping the content, and I genuinely appreciate your assistance.
Moreover, the fear of forced medical procedures and clinicians' decisions further emphasize the intersectionality of race, gender, and power hierarchies, perpetuating vulnerabilities among Indigenous women within the healthcare system. "Revise another study reporting similar results about women's fear of unnecessary medical procedures.<br><br>de Jesús-García A, Paredes-Solís S, Valtierra-Gil G, Los Santos FRS, Sánchez-Gervacio BM, Ledogar RJ, Andersson N, Cockcroft A. Associations with perineal trauma during childbirth at home and in health facilities in indigenous municipalities in southern Mexico: a cross-sectional cluster survey. BMC Pregnancy Childbirth. 2018 May 31;18(1):198. doi: 10.1186/s12884-018-1836-8. PMID: 29855266; PMCID: PMC5984371."	Dear respected academic editor, I would like to convey my sincere gratitude for your valuable comment. Your feedback is greatly appreciated. In the revised manuscript, I have cited the study conducted by de Jesús-García A et al. and mentioned that another study reported similar results regarding women's fear of unnecessary medical procedures.<br><br>Your insights have been crucial in enhancing the content, and I genuinely value your assistance.
To sum up, this study uncovers the multifaceted impact of racism on Garo Indigenous women's healthcare access. Through the lens of intersectionality, it becomes evident that race, gender, culture, and socioeconomic status intersect to compound the barriers they face. This intersectional perspective deepens our understanding of institutional and personally-mediated racism, shedding light on how it shapes the agency of these women. The study contributes to the previous research on racism and healthcare (3,31,45,68), emphasizing the need to address both individual prejudices and systemic inequities in healthcare systems. The study shows based on the evidence of the reports of Garo women: there is strong racism at different levels, and it strongly compromises biomedical healthcare access. Garo women count on a strong traditional medicine system to attend maternal healthcare. Garo women agency derives in their decision to avoid hospitals were they and their culture is mis-respected. Therefore, to increase biomedical healthcare access for Garo women racism needs to be present in the institutional and relational level. "This paragraph does not add to the paper. Please consider deleting it."	Dear respected academic editor, I want to express my profound gratitude for your valuable comment. Your feedback is greatly valued. In accordance with your guidance, I have removed the mentioned paragraph from the revised manuscript.<br><br>Your insights have played a significant role in refining the content, and I genuinely appreciate your assistance.
Therefore, the current study suggests for implementing intercultural competency training program (CICTP) for the clinicians. This program should incorporate 'cultural humility' approaches, which recognize and respect intersecting identities of race, gender, cultural identity, and socioeconomic status (23). "Revise English".	Dear respected academic editor, I wish to convey my deep appreciation for your valuable comment. Your feedback is highly regarded. In response to your guidance, I have made the necessary revisions to the English in the revised manuscript.

	Your insights have significantly contributed to the improvement of my work, and I genuinely value your assistance.
Please, add a short section of no more than one paragraph with strength and limitations of the study before the concussion.	Dear respected academic editor, I want to express my sincere gratitude for your valuable comment. Your feedback is of great importance to me. In accordance with your guidance, I have added a new section titled "Strengths and Limitations of the Study" before the Conclusion section in the revised manuscript.<br><br>Your insights have been instrumental in enhancing the organization of the manuscript, and I genuinely value your assistance.

Table 2: Response to the Reviewer's Comments

• Pay attention in the methodological section that the verb ending is uniform in the past tense.	Dear respected Reviewer,<br>I want to express my gratitude for your thorough review and attention to detail. Ensuring consistency in the verb endings in the past tense throughout the methodological section is an essential aspect of maintaining the quality of the paper. I carefully reviewed and revised the section to address this concern, and I appreciate your valuable input in maintaining the linguistic consistency and precision of the content.
• On page 18, the methodological design is qualitative descriptive approach. No qualitative description.	Dear respected Reviewer,<br>Thank you for your meticulous review of the manuscript. I appreciate your keen eye for detail. I will make the necessary correction on page 18, ensuring that the methodological design is referred to as a "qualitative descriptive approach" rather than "qualitative description." Your feedback is essential in maintaining precision and clarity in the content, and I'm grateful for your contribution to the refinement of the paper.
• Trustworthiness (p.10) Author can redact this section, in a simpler way: The strategies to enhance trustworthiness in this study where…	Dear respected Reviewer,<br>I want to express my gratitude for your thoughtful review and suggestions. I agree that the section on trustworthiness (on page 10) can be revised to enhance clarity and simplicity. I made the necessary revisions to present the strategies for ensuring trustworthiness in a more straightforward and reader-friendly manner. Your feedback is highly valuable, and I appreciate your contribution to improving the manuscript. [please see the revised section 'trustworthiness'.
• Ethical considerations (P.22). Replace "privacy" with "anonymity".	Dear respected Reviewer,<br>I want to express my gratitude for your careful review of the manuscript. Your suggestion to replace "privacy" with "anonymity" in the section on ethical considerations (on page 22) is well-noted and appreciated. I agree that the term

	"anonymity" is more accurate and aligns better with the ethical considerations discussed in that section. I made the recommended change to enhance the precision of the content and ensure it accurately reflects the research ethics.<br>Thank you for your valuable input, which contributes to the overall quality of the paper.
Results:<br>• (P. 27). Correct the capital "P" in Participants.<br>• The last quote of the participants (p.23) is significantly relevant: not only it shows women agency, but also indicates the perceived efficacy of the traditional medicine in women healthcare.<br>• (p. 25) Please briefly clarify the discussion with the Ethiopia study and the other study about identity based racial discrimination at the institutional level. It still not clear how does this study contrast with the study in Ethiopia.	• Dear respected Reviewer,<br>Thank you for your careful review and for bringing to my attention the capitalization issue with "Participants." I apologize for the oversight, and I made the necessary correction to ensure consistency in capitalization throughout the document. Your attention to detail is greatly appreciated, and it helped improve the overall quality of the manuscript.<br>• Dear respected Reviewer,<br>Thank you for your kind and valuable observation. I appreciate your observation and recognition of the significance of the last quote from the participants on page 23.<br>• Dear respected Reviewer,<br>Thank you for your valuable comment. I appreciate your interest in further clarification regarding the contrast between this study and the research in Ethiopia. To provide a more convincing explanation, it's important to emphasize that in this study conducted in Bangladesh, the primary obstacle for Garo Indigenous women's maternal healthcare access is institutional racism within healthcare institutions, which stems from Indigenous identity-based discrimination. In contrast, in Ethiopia, the challenges faced by Indigenous women were more closely related to the actual unavailability of healthcare resources, along with cultural norms and a shortage of female healthcare professionals. By underlining these differences, I aim to illustrate how various factors contribute to racial discrimination in distinct regional contexts. **[please see page 25, 'discussion section', lines in red color]**<br>Thank you for your understanding and guidance.

Your diligent and thoughtful review has been instrumental in improving the manuscript, and I am deeply grateful for your contribution.

Sincerely,
The Author
